# Cost-Effectiveness of Whole-Genome vs Whole-Exome Sequencing Among Children With Suspected Genetic Disorders

**DOI:** 10.1001/jamanetworkopen.2023.53514

**Published:** 2024-01-26

**Authors:** Mario Cesare Nurchis, Francesca Clementina Radio, Luca Salmasi, Aurora Heidar Alizadeh, Gian Marco Raspolini, Gerardo Altamura, Marco Tartaglia, Bruno Dallapiccola, Elena Pizzo, Maria Michela Gianino, Gianfranco Damiani

**Affiliations:** 1 School of Economics, Università Cattolica del Sacro Cuore, Rome, Italy; 2 Department of Woman and Child Health and Public Health, Fondazione Policlinico Universitario A. Gemelli Istituto di Ricovero e Cura a Carattere Scientifico (IRCCS), Rome, Italy; 3 Molecular Genetics and Functional Genomics, Ospedale Pediatrico Bambino Gesù IRCCS, Rome, Italy; 4 Department of Economics and Finance, Università Cattolica del Sacro Cuore, Rome, Italy; 5 Department of Health Sciences and Public Health, Section of Hygiene, Università Cattolica del Sacro Cuore, Rome, Italy; 6 Department of Applied Health Research, University College London, London, United Kingdom; 7 Department of Public Health Sciences and Paediatrics, Università di Torino, Turin, Italy

## Abstract

**Importance:**

The diagnosis of rare diseases and other genetic conditions can be daunting due to vague or poorly defined clinical features that are not recognized even by experienced clinicians. Next-generation sequencing technologies, such as whole-genome sequencing (WGS) and whole-exome sequencing (WES), have greatly enhanced the diagnosis of genetic diseases by expanding the ability to sequence a large part of the genome, rendering a cost-effectiveness comparison between them necessary.

**Objective:**

To assess the cost-effectiveness of WGS compared with WES and conventional testing in children with suspected genetic disorders.

**Design, Setting, and Participants:**

In this economic evaluation, a bayesian Markov model was implemented from January 1 to June 30, 2023. The model was developed using data from a cohort of 870 pediatric patients with suspected genetic disorders who were enrolled and underwent testing in the Ospedale Pediatrico Bambino Gesù, Rome, Italy, from January 1, 2015, to December 31, 2022. The robustness of the model was assessed through probabilistic sensitivity analysis and value of information analysis.

**Main Outcomes and Measures:**

Overall costs, number of definitive diagnoses, and incremental cost-effectiveness ratios per diagnosis were measured. The cost-effectiveness analyses involved 4 comparisons: first-tier WGS with standard of care; first-tier WGS with first-tier WES; first-tier WGS with second-tier WES; and first-tier WGS with second-tier WGS.

**Results:**

The ages of the 870 participants ranged from 0 to 18 years (539 [62%] girls). The results of the analysis suggested that adopting WGS as a first-tier strategy would be cost-effective compared with all other explored options. For all threshold levels above €29 800 (US $32 408) per diagnosis that were tested up to €50 000 (US $54 375) per diagnosis, first-line WGS vs second-line WES strategy (ie, 54.6%) had the highest probability of being cost-effective, followed by first-line vs second-line WGS (ie, 54.3%), first-line WGS vs the standard of care alternative (ie, 53.2%), and first-line WGS vs first-line WES (ie, 51.1%). Based on sensitivity analyses, these estimates remained robust to assumptions and parameter uncertainty.

**Conclusions and Relevance:**

The findings of this economic evaluation encourage the development of policy changes at various levels (ie, macro, meso, and micro) of international health systems to ensure an efficient adoption of WGS in clinical practice and its equitable access.

## Key Points

**Question:**

Is whole-genome sequencing (WGS) more cost-effective than whole-exome sequencing for children with suspected genetic disorders?

**Findings:**

The results of this economic evaluation of a cohort of 870 pediatric patients suggest that adopting WGS as a first-tier strategy would be cost-effective at a willingness-to-pay threshold of €30 000 to €50 000 (US $32 625-$54 375), specifically for the diagnosis of severely ill infants with suspected genetic disorders.

**Meaning:**

These findings suggest that wider use of WGS may minimize diagnostic delays and facilitate timely implementation of appropriate treatments.

## Teaser

This economic evaluation estimates the cost-effectiveness of whole-genome sequencing compared with whole-exome sequencing and conventional testing in children with suspected genetic disorders over their lifetime.

## Introduction

Pediatric rare diseases, typically resulting from genetic variations,^1^ can emerge from before birth to childhood, posing notable clinical challenges. First, diagnosing rare diseases and other genetic conditions can be daunting due to vague or poorly defined clinical symptoms or lack of knowledge around many disorders as well as around the extension and functional impact of genetic variations.^2^ Conventional diagnostic tests typically involve sequential single-gene analysis,^3^ while for many heterogeneous clinical conditions, multigene panels or genome-wide sequencing methods are necessary to identify the causative variations.^4^ Various factors greatly complicate diagnosing rare diseases in the pediatric population, such as the considerable number of disease-associated genes, the absence of pathognomonic features or signs, and the heterogeneous nature of the underlying pathogenic changes.^5^ The process is often lengthy, requires prompt recognition of suggestive features and timely referral to different specialists,^6^ and may require multiple attempts.

Next-generation sequencing (NGS) technologies have greatly enhanced the diagnosis of genetic diseases by expanding the ability to sequence large parts of the genome.^7^ Whole-exome sequencing (WES) analyzes protein-coding sections of the genome, while whole-genome sequencing (WGS) analyzes both coding and noncoding regions. Both WES and WGS are increasing the diagnostic rate in children with suspected genetic disorders, helping to minimize diagnostic delays and facilitate the timely initiation of appropriate treatments. Moreover, they are more efficient in identifying genetic diseases and offer higher clinical utility.^8,9^ Of note, the increasing number of diagnoses must be balanced against false-positive rates, especially for disorders where genetic testing identifies the risk of developing the disease rather than detecting the disease. Despite known benefits of WES, WGS adoption lags due to complexity and cost, intensifying health burdens and family strains.^10,11^

Diseases contributing to the US health care costs of $4.6 to $17.5 billion represent 12% to 47% of children’s inpatient care expenses. In 2019, rare diseases cost US $966 billion, with 57% nonmedical and 43% direct medical expenses.^12,13^ To date, a limited number of studies^14,15^ have investigated the cost-effectiveness of WGS and WES in pediatric populations with potential genetic disorders, but they have shown encouraging results. Therefore, this study estimates the cost-effectiveness of WGS compared with WES and conventional testing in children with suspected genetic disorders over their lifetime. The main methodological contribution of our report estimates a bayesian Markov model. The main advantage of this method is the possibility of combining it with Markov chain Monte Carlo (MCMC) algorithms to perform the probabilistic sensitivity analysis (PSA). In this way, convergence to the target distribution of interest, that is, the posterior distribution of costs and effects, is guaranteed and can be tested with diagnostic tools.

## Methods

This economic evaluation followed the Consolidated Health Economic Evaluation Reporting Standards (CHEERS) reporting guideline. Ethical review and approval were obtained from the Institutional Review Board of Ospedale Pediatrico Bambino Gesù (OPBG), Rome, Italy, and written informed consent was obtained from participants or guardians. The analysis was performed according to the perspective of the Italian National Health Service (NHS).

### Target Population

In this model, 870 patients (aged 0-18 years) suspected of having a rare genetic disease, but still undiagnosed, were consecutively enrolled and underwent testing in the OPBG’s Undiagnosed Patients Program between January 1, 2015, and December 31, 2022. Clinical suspicion for each patient was based on results of a multidisciplinary evaluation, including craniofacial appearance, anthropometric measurements, and a detailed clinical appraisal on a case-to-case basis. This process guided the selection of WES or WGS analyses based on clinical spectrum and/or results of previously performed testing.

The patients were seeking evaluations for varied clinical presentations, including encephalopathy, epilepsy, and syndromic intellectual disabilities. Those diagnosed prenatally (eg, trisomy 21) or at birth (eg, cystic fibrosis) or needing single-gene tests (eg, neurofibromatosis) were excluded. The clinical pathway was retrieved using the scientific literature. Briefly, after consultation with medical specialists in the pediatric field, if clinical conditions were suggestive, patients were referred to a genetic specialist who performed genetic consultation and chose the genomic test.^16^ Overall, 300 patients received the standard of care (SOC); 480, WES; and 90, WGS.

### Intervention and Comparators

The cost-effectiveness model focused on comparing first-tier WGS, the intervention evaluated in the analysis, with 4 comparators: SOC, first-tier WES, second-tier WES, and second-tier WGS. In this study, SOC referred to the combination of standard genetic tests and diagnostic investigations commonly used in routine clinical practice, such as single-gene panels, multigene panels, chromosomal microarray (CMA), and karyotype. Whole-exome sequencing was not considered part of the standard diagnostic workup. A first-tier test was defined as the first diagnostic test usually used when a patient without a diagnosis visits the hospital.

### Time Horizon, Currency, Discount Rate, and Threshold

Within our Markov simulation, the time horizon was divided into several discrete periods known as Markov cycles. Sixty cycles, each of annual length, were chosen as the period. Based on the guidelines provided by the National Institute for Health and Care Excellence^17^ and children’s clinical features, the selected timeframe could be sufficient for evaluating intervention benefits; in fact, some genetic disorders may cause severe, life-threatening complications that shorten their life expectancy.^18,19,20,21^ All costs are presented in 2022 euros (€) and US dollars (US $), adjusted using historical exchange rates^22^ and the Consumer Price Index.^23^ The conversion factor was 1.0875 (ie, €1 = US $1.0875). A discount rate of 3% per year was applied to both costs and effects.^24,25^ To guide the decision-making process on the endorsed alternative, the Eurozone threshold, which ranges between €30 000 and €50 000 (US $32 625-$54 375), was used.

### Type of Model

**Figure 1.**
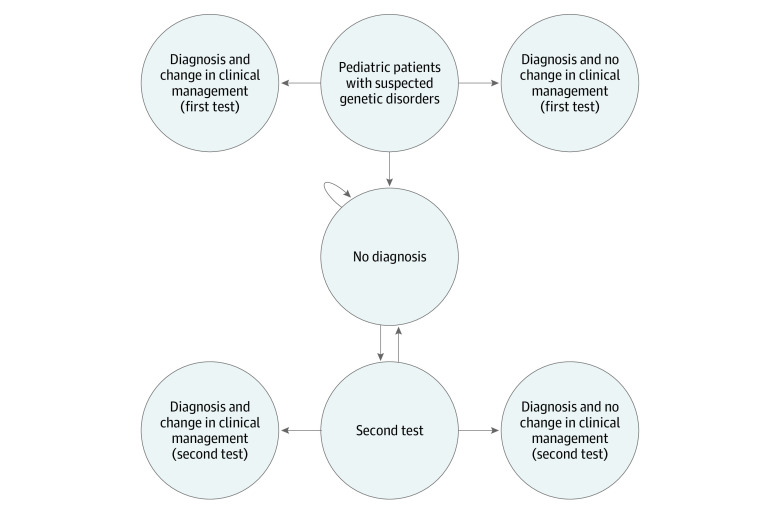
Markov Model Structure The Markov model structure represents the path followed by an individual pediatric patient suspected of having a genetic disorder. After entering this model in the symptomatic health state at the time of the patient’s first contact, access and continuity of care begin, and individuals with suspected genetic disorders undergo a genetic or genomic test (either a standard genetic test or next-generation sequencing [NGS], depending on the adopted strategy). From there, patients may receive a definitive diagnosis and potentially a different clinical management, a definitive diagnosis with no different clinical management, or remain without a definite diagnosis. In case of a multistep strategy (ie, second-line whole-genome sequencing or second-line whole-exome sequencing), undiagnosed patients undergo a second test, namely NGS, which can be diagnostic and may or may not be followed by a change in clinical management or remain undiagnosed.

A bayesian Markov model was set up. Figure 1 displays the model, depicting all the Markov states.

On first contact in a symptomatic health state, individuals with potential genetic disorders undergo genetic tests, either standard or NGS based on the strategy. Subsequently, they might be diagnosed with or without altered clinical management or remain undiagnosed. In multistep strategies including second-line WES or WGS, undiagnosed patients receive another NGS test, potentially leading to a diagnosis with potential management changes or continued uncertainty. The bayesian model presents a comprehensive probability distribution for a dichotomic outcome, θ = (*c*, *e*), consisting of potential combinations of costs and effects. Additional information about the inference is found in the eMethods in Supplement 1.

### Model Inputs

Transition probabilities for diagnosis were sourced from the OPBG’s records. Clinical management change probabilities came from scientific literature. The model incorporated only direct costs. Clinical geneticists retrospectively reviewed all procedures using the hospital’s information system. Diagnostic, management, and therapeutic costs, including initial and continued care, were evaluated. Testing costs followed Italian NHS tariffs. The cost differences associated with each pathway arose from the consumable costs and the capacity to analyze multiple samples concurrently using identical equipment, such as the sequencing chip. In particular, the SOC analysis allowed for the examination of 96 samples with a single chip, the WES analysis accommodated 24 samples, and the WGS analysis was limited to a single sample per chip. Additionally, there were differences in data analysis costs, quantified in economic terms as personnel expenses, representing the human resource hours dedicated to each analysis. Differences also involved the training costs required for the personnel engaged in genomic analysis. Care costs were estimated according to resource use and costs attached to them. Post-WES or post-WGS procedure costs, as well as diagnostic odyssey costs, were drawn from scientific literature. The diagnostic odyssey for patients with a suspected genetic disorder was defined as a complex and often prolonged journey of medical evaluations and testing to identify the underlying genetic cause of their symptoms.

International agencies and scientific evidence^26,27^ suggest the use of the quality-adjusted life-year (QALY)^28,29^ for its standardization, promoting broader comparisons of medical technologies and resource allocation. However, when evaluating genomic technologies, QALY is infrequently used due to its data requirements and shortcomings^30^; it overlooks outcomes such as personal utility and family spillover effects.^31,32,33^ Given patient population and care pathway diversity, QALY impacts were not collected. Instead, diagnostic yield was the primary outcome, being a prevalent measure in WGS studies.^8,9,34^ This choice stemmed from a lack of strong data to determine QALYs from clinical management shifts^35^ since no follow-up was available. The analysis excluded outcomes related to family, although costs of WES and WGS incorporated confirmatory and trio testing with parents. Table 1 depicts the parameters used in the base case analysis for each strategy.^8,27,36,37,38,39^

**Table 1.** List of Parameters Used in the Economic Model

Model parameter	Base estimate^a^	Distribution	Source
Diagnostic yield following testing strategy			
First-line SOC	0.43^b^	Beta	Hospital data
First-line WES	0.58^b^	Beta	Hospital data
First-line WGS	0.64^b^	Beta	Hospital data
Change in clinical management SOC	0.06	Beta	Clark et al,^8^ 2018
Change in clinical management WES	0.17	Beta	Clark et al,^8^ 2018
Change in clinical management WGS	0.27	Beta	Clark et al,^8^ 2018
Costs			
First contact, access, and continuity of care (SOC)	€29 870 (US $32 484)^c^	Log-normal^d^	Hospital data
First contact, access, and continuity of care (WES)	€61 704 (US $67 103)^c^	Log-normal^d^	Hospital data
First contact, access, and continuity of care (WGS)	€79 170 (US $86 097)^c^	Log-normal^d^	Hospital data
SOC testing	€450 (US $489)^e^	Log-normal^d^	Hospital data
WES testing	€1800 (US $1958)^e^	Log-normal^d^	Hospital data
WGS testing	€3700 (US $4024)^e^	Log-normal^d^	Hospital data
Change in clinical management SOC	€4241 (US $4612)	Uniform	Greely et al,^36^ 2011
Change in clinical management WES	€9611 (US $10 452)	Uniform	Stark et al,^27^ 2019
Change in clinical management WGS	€15 785 (US $17 166)	Uniform	Farnaes et al,^37^ 2018
After WES or WGS testing costs with diagnosis	€92 (US $100)	Log-normal^d^	Lavelle et al,^38^ 2022
After WES or WGS testing costs without diagnosis	€162 (US $176)	Log-normal^d^	Lavelle et al,^38^ 2022
Diagnostic odyssey	€2375 (US $2583)	Log-normal^d^	Radio et al,^39^ 2019

Abbreviations: SOC, standard of care; WES, whole-exome sequencing; WGS, whole-genome sequencing.

^a^ These estimates are calculated on a yearly basis.

^b^ The hospital cohorts of 300 pediatric patients associated with SOC, 480 with WES, and 90 with WGS testing strategies were used to estimate diagnostic yield.

^c^ The accounted costs encompass those generated from diagnostic, management, and therapeutic procedures.

^d^ Specification of log-normal distributions is made by the lower and upper limits of the 95% CIs.

^e^ Costs related to the testing strategies also include labor, supplies, bioinformatics, and equipment expenses.

### Model Outcomes

The primary outcomes of the economic model encompassed the number of diagnoses and the expected costs. The projected cost for each Markov cycle was determined by multiplying the cost by the quantity of resources required during the patient’s duration in a specified health state. Comparing the 2 interventions allowed for the calculation of the incremental cost-effectiveness ratio (ICER) and net monetary benefits for the investigated testing strategies.

### Statistical Analysis

To tackle uncertainty related to the calculated outcomes, a PSA was performed. A specific distribution was assigned to each parameter: a beta distribution was applied for diagnostic yields and changes in clinical management, while costs were characterized by a log-normal distribution. In the robustness analysis, costs related to change in clinical management after diagnosis were considered. This information is not widely available in the scientific literature, and the available studies are focused on specific populations.^27,36,37^ Therefore, a uniform distribution, defined over a large interval of values, was used to reflect its uncertainty.

These distributions match prior researchers’ knowledge about the model parameters. The log-normal or the beta distribution are the informative priors, that is, they assign higher probabilities to values close to the mean. The uniform distribution is, instead, a vague prior since it provides equal probabilities to each value in the specified intervals. Table 1 lists an extensive list of model parameters along with the distributions used in the PSA.

In the PSA, the Gibbs algorithm was used to perform 100 000 MCMC simulations, which entailed drawing random sets of parameter values from the associated probability distributions for each model parameter and subsequently calculating incremental costs, incremental effectiveness, and ICERs for each set. Additional information about how findings were presented is provided in the eMethods in Supplement 1.

Data were analyzed from January 1 to June 30, 2023. All analyses were run using R software, version 4.2.3 (R Project for Statistical Computing).

## Results

### Base Case Results

A total of 870 patients were included in the analysis (331 [38%] boys and 539 [62%] girls). As expected, the SOC testing strategy had both the lowest costs and the lowest diagnostic yield for the target population. The base case findings showed that considering the chosen threshold of €30 000 to €50 000 (US $32 625-$54 375) per additional diagnosis, first-line WGS would be a cost-effective strategy compared with SOC (ICER of €24 824 [95% CI, €23 255-€26 615] [US $26 996 (95% CI, $25 290-$28 944)]), first-line WES (ICER of €29 728 [95% CI, €20 609-€53 171] [US $32 329 (95% CI, $22 412-$57 823)]), second-line WES (ICER of €22 127 [95% CI, €18 806-€26 863] [US $24 063 (95% CI, $20 452-$29 214)]), or second-line WGS (ICER of €21 458 [95% CI, €15 075-€37 192] [US $23 336 (95% CI, $16 394-$40 446)]) per added diagnosis. Table 2 lists the mean discounted costs, discounted effectiveness, and the base case results for the simulated cohort.

**Table 2.** Base Case Results Over Lifetime Horizon

Strategy	Costs (95% CI), €	Difference in costs (95% CI), €	Effectiveness (95% CI), No. of diagnoses	Difference in effectiveness (95% CI), No. of diagnoses	ICER (95% CI), €^a^	Net monetary benefit, €^b^
WGS	1 862 645 603 (1 832 439 308-1 864 945 898)	NA	178 367 (171 072-183 662)	NA	1 [Reference]	NA
SOC	431 386 069 (414 457 635-439 481 503)	1 431 259 534 (1 417 981 673-1 439 464 395)	120 710 (118 276-123 144)	57 657 (52 796-60 518)	24 824 (23 255-26 615)	298 450 466
WES	1 437 856 531 (1 399 756 440-1 456 258 621)	424 789 072 (408 687 277-432 682 868)	164 078 (153 987-179 169)	14 289 (4493-17 085)	29 728 (20 609-53 171)	3 880 928
WES after SOC	572 335 469 (531 313 408-594 335 530)	1 290 310 134 (1 270 610 368-1 301 125 900)	120 053 (104 993-138 114)	58 314 (45 548-66 079)	22 127 (18 806-26 863)	459 109 866
WGS after SOC	657 296 506 (622 291 451-687 629 561)	1 205 349 097 (1 177 316 337-1 210 147 857)	122 194 (102 140-162 249)	56 173 (21 413-68 932)	21 458 (15 075-37 192)	479 840 903

Abbreviations: ICER, incremental cost-effectiveness ratio; NA, not applicable; SOC, standard of care; WES, whole-exome sequencing; WGS, whole-genome sequencing.

Conversion factor: To convert euros to US dollars, multiply by 1.0875.

^a^ Computed as the difference between the costs of the intervention (ie, WGS) and the comparators (ie, SOC, WES, second-line WES, and second-line WGS) over the difference between the number of diagnoses of the intervention and the comparators.

^b^ Computed considering the lower bound (ie, €30 000 [US $32 625]) of the Eurozone threshold.

### Sensitivity Analysis

**Figure 2.**
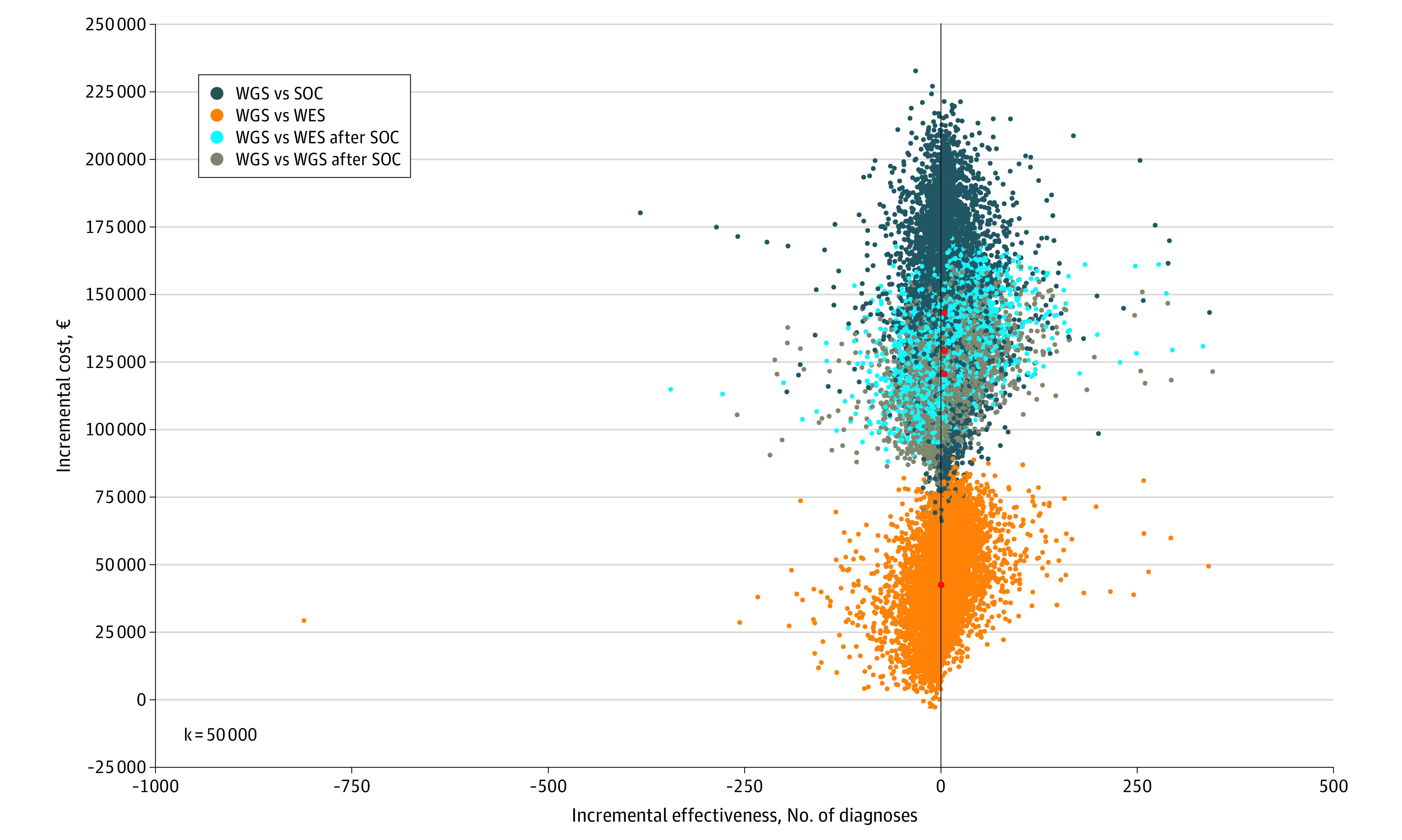
Cost-Effectiveness Plane From Probabilistic Sensitivity Analysis The cost-effectiveness plane represents the combined distribution of the incremental expected costs (y-axis) and the incremental expected effectiveness (x-axis) from the investigated alternatives. Incremental means the difference between the 2 options, for example, the difference between whole-genome sequencing (WGS) and standard of care (SOC). The dark blue, light blue, gray, and orange dots are individual simulations coming from probabilistic sensitivity analysis; the red dots represent the incremental cost-effectiveness ratios. The area to the right of the vertical line represents the cost-effective region. WES indicates whole-exome sequencing.

Figure 2 depicts the cost-effectiveness plan confirming the robustness of the initial findings considering the chosen threshold. Furthermore, contour plots revealed that 63% of the simulated points for SOC, 54% for WES, 64% for second-line WES, and 63% for second-line WGS testing strategies lay in the northeast quadrant of the cost-effectiveness plane, where WGS is characterized by higher effectiveness and higher costs with respect to the other alternatives (eFigures 1-4 in Supplement 1).

eFigures 5 and 6 in Supplement 1 depict the cost-effectiveness acceptability curve and the cost-effectiveness acceptability frontier curve, respectively. Our findings highlight that SOC was the optimal decision for a willingness-to-pay (WTP) threshold lower than €23 300 (US $25 339) per diagnosis, while WES was optimal for a WTP threshold between €23 300 and €29 800 (US $32 408) per diagnosis. For all WTP levels above €29 800 per diagnosis that were tested up to €50 000 (US $54 375) per diagnosis, first-line WGS vs second-line WES (ie, 54.6%) had the highest probability of being cost-effective, followed by first-line WGS vs second-line WGS (ie, 54.3%), first-line WGS vs SOC (ie, 53.2%), and first-line WGS vs first-line WES (ie, 51.1%). As shown in eFigure 7 in Supplement 1, expected incremental benefit values were positive for all testing strategies at a WTP of €23 300 (US $25 339), equaling 145 161 for first-line WGS vs SOC, 28 970 for first-line WGS vs first-line WES, 162 539 for first-line WGS vs second-line WES, and 160 330 for first-line WGS vs second-line WGS.

### Value of Information Analysis

**Figure 3.**
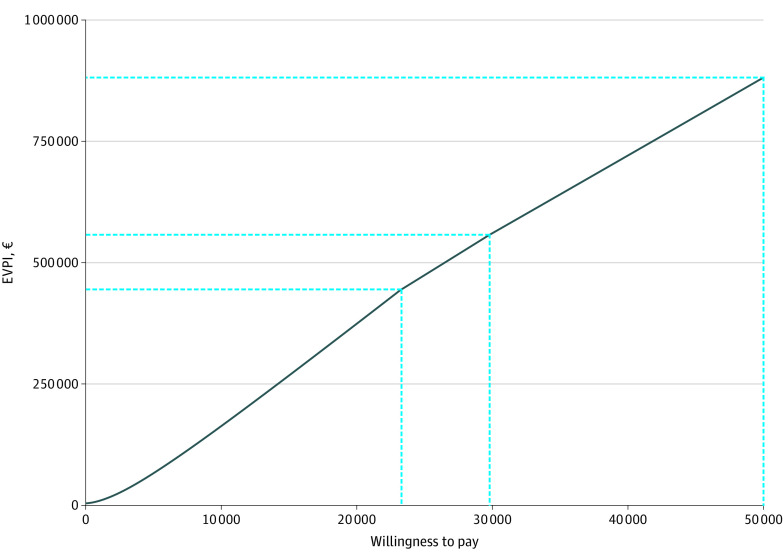
Population Expected Value of Perfect Information (EVPI) Curve The figure depicts the population EVPI (y-axis) over a range of willingness-to-pay or acceptability thresholds (x-axis) in the Italian pediatric population aged 0 to 18 years.

Based on the PSA simulations, the expected value of partially perfect information per patient amounted to €880 982 (US $958 068) on a WTP threshold of €50 000 (US $54 375) per diagnosis (Figure 3). The value decreased to €560 235 (US $609 256) considering a WTP threshold of €30 000 (US $32 625) per diagnosis. Additional findings are provided in the eResults and eFigures 8 to 11 in Supplement 1.

### Robustness Analysis Including Costs of Change in Clinical Management

Robustness analysis findings are provided in the eResults and eTable 1 in Supplement 1. Including the costs of change in management, using first-line WGS would be a cost-effective strategy compared with the other strategies.

### Diagnostic Performance for MCMC Simulation

Overall, the MCMC simulation performed optimally with chains achieving convergence, impressive effective sample sizes, and accurately derived posterior distributions, thereby enhancing trust in the accuracy and consistency of results. Additional details are reported in the eResults, eFigures 12 to 58, and eTable 2 in Supplement 1.

## Discussion

Our data suggest that WGS is cost-effective for diagnosing infants with potential genetic disorders at a WTP threshold of €30 000 to €50 000 (US $32 625-$54 375). Our findings contribute to previous studies exploring the cost-effectiveness of WGS^15^ and WES.^40,41,42^ Runheim et al^15^ discussed the cost-effectiveness of WGS vs CMA as first-line strategies in diagnosing rare diseases (ie, neurodevelopmental disorders) in children and infants. The mean health care cost per patient in the cohort undergoing WGS was US $2339 lower (95% CI, −$12 238 to $7561) compared with the one receiving CMA, respectively, with US $2330 in lower costs for outpatient care (95% CI, −$3992 to −$669), besides showing a higher diagnostic yield (CMA, 20.1%; WGS, 24.7%).^15^ Another study^43^ highlighted WGS’s economic superiority and diagnostic yield over WES (WGS, 54%; WES, 41%) for mendelian disorders. Additional research^44^ echoes our findings, emphasizing the cost-effectiveness of first-line WGS for ill children. Despite a different modeling approach, their cost-effectiveness findings (ICER of US $15 904) corroborate our results.^44^ A 2018 review^34^ showcased health economics of WGS and WES, highlighting children as the primary study participants and revealing 2 distinct cost (from US $555 to $5169 for WES and from US $1906 to $24 810 for WGS) and diagnostic yield (from 3% to 79% for WES and from 17% to 73% for WGS) ranges. The cost-effectiveness of WGS over WES was demonstrated through a pooled assessment of their incremental net benefits,^45^ while Clark et al^8^ demonstrated that the cumulative diagnostic yield of WGS (ie, 41%) surpassed that of WES (ie, 36%) and SOC (ie, 10%).

In a pivotal moment, Italy’s pace in reimbursing genomic sequencing, especially WGS, trails behind. Despite its elevated costs and modest clinical gains, WGS’s adoption lags compared with WES,^34^ contrasting with American College of Medical Genetics and Genomics’ pediatric recommendations.^46^

This study informs nuanced decision-making throughout the health care landscape. At the macro level, defining an ad hoc diagnosis related group tariff could encompass the cases in which WGS has been demonstrated to be cost-effective as a first-line test. Furthermore, genomic policies are needed to properly regulate and guarantee the provision and sustainability of WGS within the health care services; patients with suspected genetic diseases often experience a diagnostic odyssey, with long periods of uncertainty, leading to poor quality of life and clinical outcomes.^47^ Involved infrastructures should ensure cross-communication and collaboration among all levels of assistance (ie, tertiary, secondary, and primary care facilities) to streamline the diagnostic workflow and to ensure access to and continuity of care for pediatric patients with suspected genetic disorders.

At the meso level, local health units should manage procurement and surveillance of WGS implementation to guarantee its efficient adoption and to monitor its impact on health outcomes. At the micro level, advancing genomic awareness of rare diseases among health professionals using WGS is crucial to enhance individual medical knowledge and to ameliorate personalized health care services. Last, at the nano level, promoting awareness about rare diseases among patients and caregivers can enhance understanding of symptoms and available tests, boosting engagement and compliance.

### Limitations

This study has some limitations. Effectiveness was gauged via clinical outcomes, not QALYs, making cost-effectiveness interpretation and comparison challenging. Other studies, focusing on follow-up and longer-term impact of genomic sequencing for rare disease diagnoses, have adopted QALYs as a main outcome measure.^26,27^

Although reanalysis of WES data might improve the diagnostic yield,^48^ the model did not include it, as it is not yet a standard procedure in Italy.^49^ Incorporating this factor could increase WES and WGS costs relative to standard practices, yet it could be cost-effective compared with a single-analysis WES or WGS. The rationale may be due to the reduced cost of reanalysis and its likelihood of making further diagnoses. Another limit was overlooking that WGS or WES might reveal incidental findings, which might enhance their estimated cost-effectiveness. A prior study^50^ has shown that despite increasing costs, reporting incidental findings also improves health benefits. Nevertheless, geneticists debate about whether parents should be informed about incidental findings in their children relating to adulthood-onset diseases.^51^ Other constraints were the unavailability of Italian primary data for some cost parameters, requiring us to use estimates from Canada and the US, and not including mortality as an absorbing health state. Current data on pediatric mortality rates in genetic disorders is limited and heterogeneous, affecting model accuracy. Our analysis overlooks additional efforts required for variant interpretation in noncoding regions with WGS compared with WES and SOC. Despite 85% of pathogenic genetic variants being exonic,^52^ WGS uncovers numerous noncoding, yet unknown, region variants that are daunting to interpret due to the dearth of data to categorize them. Additionally, omitting data storage needs for WGS and WES is another potential limitation given WGS’s resource-intensive demands.^34^ The analysis did not include structural rearrangements analyses costs, which are more often necessary after WES than WGS.^16,53^

## Conclusions

The findings of this economic evaluation suggest that further cost-effectiveness analyses should include modeling societal costs (and not only direct costs) and mortality due to rare diseases in the pediatric population with suspected genetic disorders by adding the related absorbing health state in the model. Moreover, in planning future primary research projects, change in management should be included among the main outcomes to allow estimation of the costs associated with it within the diagnostic workflow. This study champions WGS over WES as a first-tier diagnostic strategy for its cost-effectiveness, especially for children with suspected rare diseases. Italy’s NHS needs policy shifts for efficient WGS adoption that are reinforced by further clinical and economic evidence.

## Notes

Supplement 1. **eMethods.** Type of Model and Model Analysis **eResults.** Information Analysis, Robustness Analysis, and Diagnostic Performance **eFigure 1.** Contour Plot for WGS vs SOC **eFigure 2.** Contour Plot for WGS vs WES **eFigure 3.** Contour Plot for WGS vs Second-Line WES **eFigure 4.** Contour Plot for WGS vs Second-Line WGS **eFigure 5.** Cost-Effectiveness Acceptability Curve (CEAC) **eFigure 6.** Cost-Effectiveness Acceptability Frontier Curve (CEAF) **eFigure 7.** Expected Incremental Benefit (EIB) for Each Testing Strategy **eFigure 8.** Population Expected Value of Partially Perfect Information (EVPPI) Curve for Transition Probabilities **eFigure 9.** Population Expected Value of Partially Perfect Information (EVPPI) Curve for Costs **eFigure 10.** Population Expected Value of Partially Perfect Information (EVPPI) Curve for Effectiveness Measure (ie, Diagnostic Yield) **eFigure 11.** Information-Rank Plot Reporting a Ranking of the Model Parameters in Terms of Their Impact on the Expected Value of Information **eFigure 12.** Convergence Diagnostics for the Output of JAGS Related to the Cost Parameter of the SOC Strategy in the First Health State **eFigure 13.** Convergence Diagnostics for the Output of JAGS Related to the Cost Parameter of the SOC Strategy in the Second Health State **eFigure 14.** Convergence Diagnostics for the Output of JAGS Related to the Cost Parameter of the SOC Strategy in the Third Health State **eFigure 15.** Convergence Diagnostics for the Output of JAGS Related to the Cost Parameter of the SOC Strategy in the Eighth Health State **eFigure 16.** Convergence Diagnostics for the Output of JAGS Related to the Cost Parameter of the Second-Line WES Strategy in the First Health State **eFigure 17.** Convergence Diagnostics for the Output of JAGS Related to the Cost Parameter of the Second-Line WES Strategy in the Second Health State **eFigure 18.** Convergence Diagnostics for the Output of JAGS Related to the Cost Parameter of the Second-Line WES Strategy in the Third Health State **eFigure 19.** Convergence Diagnostics for the Output of JAGS Related to the Cost Parameter of the Second-Line WES Strategy in the Fourth Health State **eFigure 20.** Convergence Diagnostics for the Output of JAGS Related to the Cost Parameter of the Second-Line WES Strategy in the Fifth Health State **eFigure 21.** Convergence Diagnostics for the Output of JAGS Related to the Cost Parameter of the Second-Line WES Strategy in the Eighth Health State **eFigure 22.** Convergence Diagnostics for the Output of JAGS Related to the Cost Parameter of the Second-Line WGS Strategy in the First Health State **eFigure 23.** Convergence Diagnostics for the Output of JAGS Related to the Cost Parameter of the Second-Line WGS Strategy in the Second Health State **eFigure 24.** Convergence Diagnostics for the Output of JAGS Related to the Cost Parameter of the Second-Line WGS Strategy in the Third Health State **eFigure 25.** Convergence Diagnostics for the Output of JAGS Related to the Cost Parameter of the Second-Line WGS Strategy in the Fourth Health State **eFigure 26.** Convergence Diagnostics for the Output of JAGS Related to the Cost Parameter of the Second-Line WGS Strategy in the Fifth Health State **eFigure 27.** Convergence Diagnostics for the Output of JAGS Related to the Cost Parameter of the Second-Line WGS Strategy in the Fifth Health State **eFigure 28.** Convergence Diagnostics for the Output of JAGS Related to the Cost Parameter of the WES Strategy in the First Health State **eFigure 29.** Convergence Diagnostics for the Output of JAGS Related to the Cost Parameter of the WES Strategy in the Second Health State **eFigure 30.** Convergence Diagnostics for the Output of JAGS Related to the Cost Parameter of the WES Strategy in the Third Health State **eFigure 31.** Convergence Diagnostics for the Output of JAGS Related to the Cost Parameter of the WES Strategy in the Eighth Health State **eFigure 32.** Convergence Diagnostics for the Output of JAGS Related to the Cost Parameter of the WGS Strategy in the First Health State **eFigure 33.** Convergence Diagnostics for the Output of JAGS Related to the Cost Parameter of the WGS Strategy in the Second Health State **eFigure 34.** Convergence Diagnostics for the Output of JAGS Related to the Cost Parameter of the WGS Strategy in the Third Health State **eFigure 35.** Convergence Diagnostics for the Output of JAGS Related to the Cost Parameter of the WGS Strategy in the Eighth Health State **eFigure 36.** Convergence Diagnostics for the Output of JAGS Related to the Cost Parameter of the SOC Strategy in the First Health State (Prior) **eFigure 37.** Convergence Diagnostics for the Output of JAGS Related to the Cost Parameter of the SOC Strategy in the Second Health State (Prior) **eFigure 38.** Convergence Diagnostics for the Output of JAGS Related to the Cost Parameter of the SOC Strategy in the Third Health State (Prior) **eFigure 39.** Convergence Diagnostics for the Output of JAGS Related to the Cost Parameter of the SOC Strategy in the Eighth Health State (Prior) **eFigure 40.** Convergence Diagnostics for the Output of JAGS Related to the Cost Parameter of the Second-Line WES Strategy in the First Health State (Prior) **eFigure 41.** Convergence Diagnostics for the Output of JAGS Related to the Cost Parameter of the Second-Line WES Strategy in the Second Health State (Prior) **eFigure 42.** Convergence Diagnostics for the Output of JAGS Related to the Cost Parameter of the Second-Line WES Strategy in the Third Health State (Prior) **eFigure 43.** Convergence Diagnostics for the Output of JAGS Related to the Cost Parameter of the Second-Line WES Strategy in the Fourth Health State (Prior) **eFigure 44.** Convergence Diagnostics for the Output of JAGS Related to the Cost Parameter of the Second-Line WES Strategy in the Fifth Health State (Prior) **eFigure 45.** Convergence Diagnostics for the Output of JAGS Related to the Cost Parameter of the Second-Line WES Strategy in the Eighth Health State (Prior) **eFigure 46.** Convergence Diagnostics for the Output of JAGS Related to the Cost Parameter of the Second-Line WGS Strategy in the First Health State (Prior) **eFigure 47.** Convergence Diagnostics for the Output of JAGS Related to the Cost Parameter of the Second-Line WGS Strategy in the First Health State (Prior) **eFigure 48.** Convergence Diagnostics for the Output of JAGS Related to the Cost Parameter of the Second-Line WGS Strategy in the Third Health State (Prior) **eFigure 49.** Convergence Diagnostics for the Output of JAGS Related to the Cost Parameter of the Second-Line WGS Strategy in the Fourth Health State (Prior) **eFigure 50.** Convergence Diagnostics for the Output of JAGS Related to the Cost Parameter of the Second-Line WGS Strategy in the Fifth Health State (Prior) **eFigure 51.** Convergence Diagnostics for the Output of JAGS Related to the Cost Parameter of the Second-Line WGS Strategy in the Eighth Health State (Prior) **eFigure 52.** Convergence Diagnostics for the Output of JAGS Related to the Cost Parameter of the WES Strategy in the First Health State (Prior) **eFigure 53.** Convergence Diagnostics for the Output of JAGS Related to the Cost Parameter of the WES Strategy in the Second Health State (Prior) **eFigure 54.** Convergence Diagnostics for the Output of JAGS Related to the Cost Parameter of the WES Strategy in the Third Health State (Prior) **eFigure 55.** Convergence Diagnostics for the Output of JAGS Related to the Cost Parameter of the WES Strategy in the Eighth Health State (Prior) **eFigure 56.** Convergence Diagnostics for the Output of JAGS Related to the Cost Parameter of the WGS Strategy in the First Health State (Prior) **eFigure 57.** Convergence Diagnostics for the Output of JAGS Related to the Cost Parameter of the WGS Strategy in the Second Health State (Prior) **eFigure 58.** Convergence Diagnostics for the Output of JAGS Related to the Cost Parameter of the WGS Strategy in the Eighth Health State (Prior) **eTable 1. **Robustness Analysis Results Over Lifetime Horizon **eTable 2.** Summary Statistics of the Marginal Posterior Distribution for Each of the Model Parameters

Supplement 2.
Data Sharing Statement
