# Supplementary material for: Cost-Effectiveness of Whole-Genome vs Whole-Exome Sequencing Among Children With Suspected Genetic Disorders
Source: JAMA Netw Open. 2024 Jan 26;7(1):e2353514. doi: 10.1001/jamanetworkopen.2023.53514 (PMC10818217; doi:10.1001/jamanetworkopen.2023.53514)
Supplement: Supplement 1. — eMethods. Type of Model and Model Analysis eResults. Information Analysis, Robustness Analysis, and Diagnostic Performance eFigure 1. Contour Plot for WGS vs SOC eFigure 2. Contour Plot for WGS vs WES eFigure 3. Contour Plot for WGS vs Second-Line WES eFigure 4. Contour Plot for WGS vs Second-Line WGS eFigure 5. Cost-Effectiveness Acceptability Curve (CEAC) eFigure 6. Cost-Effectiveness Acceptability Frontier Curve (CEAF) eFigure 7. Expected Incremental Benefit (EIB) for Each Testing Strategy eFigure 8. Population Expected Value of Partially Perfect Information (EVPPI) Curve for Transition Probabilities eFigure 9. Population Expected Value of Partially Perfect Information (EVPPI) Curve for Costs eFigure 10. Population Expected Value of Partially Perfect Information (EVPPI) Curve for Effectiveness Measure (ie, Diagnostic Yield) eFigure 11. Information-Rank Plot Reporting a Ranking of the Model Parameters in Terms of Their Impact on the Expected Value of Information eFigure 12. Convergence Diagnostics for the Output of JAGS Related to the Cost Parameter of the SOC Strategy in the First Health State eFigure 13. Convergence Diagnostics for the Output of JAGS Related to the Cost Parameter of the SOC Strategy in the Second Health State eFigure 14. Convergence Diagnostics for the Output of JAGS Related to the Cost Parameter of the SOC Strategy in the Third Health State eFigure 15. Convergence Diagnostics for the Output of JAGS Related to the Cost Parameter of the SOC Strategy in the Eighth Health State eFigure 16. Convergence Diagnostics for the Output of JAGS Related to the Cost Parameter of the Second-Line WES Strategy in the First Health State eFigure 17. Convergence Diagnostics for the Output of JAGS Related to the Cost Parameter of the Second-Line WES Strategy in the Second Health State eFigure 18. Convergence Diagnostics for the Output of JAGS Related to the Cost Parameter of the Second-Line WES Strategy in the Third Health State eFigure 19. Convergence Diagnostics f [file jamanetwopen-e2353514-s001.pdf]

Supplementary Online Content

Nurchis MC, Radio FC, Salmasi L, et al. Cost-effectiveness of whole genome vs whole exome sequencing for suspected genetic disorders. *JAMA Netw Open*. 2024;7(1):e2353514. doi:10.1001/jamanetworkopen.2023.53514

eMethods. Type of Model and Model Analysis
eResults. Information Analysis, Robustness Analysis, and Diagnostic Performance
eFigure 1. Contour Plot for WGS vs SOC
eFigure 2. Contour Plot for WGS vs WES
eFigure 3. Contour Plot for WGS vs Second-Line WES
eFigure 4. Contour Plot for WGS vs Second-Line WGS
eFigure 5. Cost-Effectiveness Acceptability Curve (CEAC)
eFigure 6. Cost-Effectiveness Acceptability Frontier Curve (CEAF)
eFigure 7. Expected Incremental Benefit (EIB) for Each Testing Strategy
eFigure 8. Population Expected Value of Partially Perfect Information (EVPPI) Curve for Transition Probabilities
eFigure 9. Population Expected Value of Partially Perfect Information (EVPPI) Curve for Costs
eFigure 10. Population Expected Value of Partially Perfect Information (EVPPI) Curve for Effectiveness Measure (ie, Diagnostic Yield)
eFigure 11. Information-Rank Plot Reporting a Ranking of the Model Parameters in Terms of Their Impact on the Expected Value of Information
eFigure 12. Convergence Diagnostics for the Output of JAGS Related to the Cost Parameter of the SOC Strategy in the First Health State
eFigure 13. Convergence Diagnostics for the Output of JAGS Related to the Cost Parameter of the SOC Strategy in the Second Health State
eFigure 14. Convergence Diagnostics for the Output of JAGS Related to the Cost Parameter of the SOC Strategy in the Third Health State
eFigure 15. Convergence Diagnostics for the Output of JAGS Related to the Cost Parameter of the SOC Strategy in the Eighth Health State
eFigure 16. Convergence Diagnostics for the Output of JAGS Related to the Cost Parameter of the Second-Line WES Strategy in the First Health State
eFigure 17. Convergence Diagnostics for the Output of JAGS Related to the Cost Parameter of the Second-Line WES Strategy in the Second Health State
eFigure 18. Convergence Diagnostics for the Output of JAGS Related to the Cost Parameter of the Second-Line WES Strategy in the Third Health State
eFigure 19. Convergence Diagnostics for the Output of JAGS Related to the Cost Parameter of the Second-Line WES Strategy in the Fourth Health State
eFigure 20. Convergence Diagnostics for the Output of JAGS Related to the Cost Parameter of the Second-Line WES Strategy in the Fifth Health State

© 2024 Nurchis MC et al. *JAMA Network Open*.

eFigure 21. Convergence Diagnostics for the Output of JAGS Related to the Cost Parameter of the Second-Line WES Strategy in the Eighth Health State
eFigure 22. Convergence Diagnostics for the Output of JAGS Related to the Cost Parameter of the Second-Line WGS Strategy in the First Health State
eFigure 23. Convergence Diagnostics for the Output of JAGS Related to the Cost Parameter of the Second-Line WGS Strategy in the Second Health State
eFigure 24. Convergence Diagnostics for the Output of JAGS Related to the Cost Parameter of the Second-Line WGS Strategy in the Third Health State
eFigure 25. Convergence Diagnostics for the Output of JAGS Related to the Cost Parameter of the Second-Line WGS Strategy in the Fourth Health State
eFigure 26. Convergence Diagnostics for the Output of JAGS Related to the Cost Parameter of the Second-Line WGS Strategy in the Fifth Health State
eFigure 27. Convergence Diagnostics for the Output of JAGS Related to the Cost Parameter of the Second-Line WGS Strategy in the Fifth Health State
eFigure 28. Convergence Diagnostics for the Output of JAGS Related to the Cost Parameter of the WES Strategy in the First Health State
eFigure 29. Convergence Diagnostics for the Output of JAGS Related to the Cost Parameter of the WES Strategy in the Second Health State
eFigure 30. Convergence Diagnostics for the Output of JAGS Related to the Cost Parameter of the WES Strategy in the Third Health State
eFigure 31. Convergence Diagnostics for the Output of JAGS Related to the Cost Parameter of the WES Strategy in the Eighth Health State
eFigure 32. Convergence Diagnostics for the Output of JAGS Related to the Cost Parameter of the WGS Strategy in the First Health State
eFigure 33. Convergence Diagnostics for the Output of JAGS Related to the Cost Parameter of the WGS Strategy in the Second Health State
eFigure 34. Convergence Diagnostics for the Output of JAGS Related to the Cost Parameter of the WGS Strategy in the Third Health State
eFigure 35. Convergence Diagnostics for the Output of JAGS Related to the Cost Parameter of the WGS Strategy in the Eighth Health State
eFigure 36. Convergence Diagnostics for the Output of JAGS Related to the Cost Parameter of the SOC Strategy in the First Health State (Prior)
eFigure 37. Convergence Diagnostics for the Output of JAGS Related to the Cost Parameter of the SOC Strategy in the Second Health State (Prior)
eFigure 38. Convergence Diagnostics for the Output of JAGS Related to the Cost Parameter of the SOC Strategy in the Third Health State (Prior)
eFigure 39. Convergence Diagnostics for the Output of JAGS Related to the Cost Parameter of the SOC Strategy in the Eighth Health State (Prior)
eFigure 40. Convergence Diagnostics for the Output of JAGS Related to the Cost Parameter of the Second-Line WES Strategy in the First Health State (Prior)
eFigure 41. Convergence Diagnostics for the Output of JAGS Related to the Cost Parameter of the Second-Line WES Strategy in the Second Health State (Prior)

© 2024 Nurchis MC et al. *JAMA Network Open*.

eFigure 42. Convergence Diagnostics for the Output of JAGS Related to the Cost Parameter of the Second-Line WES Strategy in the Third Health State (Prior)
eFigure 43. Convergence Diagnostics for the Output of JAGS Related to the Cost Parameter of the Second-Line WES Strategy in the Fourth Health State (Prior)
eFigure 44. Convergence Diagnostics for the Output of JAGS Related to the Cost Parameter of the Second-Line WES Strategy in the Fifth Health State (Prior)
eFigure 45. Convergence Diagnostics for the Output of JAGS Related to the Cost Parameter of the Second-Line WES Strategy in the Eighth Health State (Prior)
eFigure 46. Convergence Diagnostics for the Output of JAGS Related to the Cost Parameter of the Second-Line WGS Strategy in the First Health State (Prior)
eFigure 47. Convergence Diagnostics for the Output of JAGS Related to the Cost Parameter of the Second-Line WGS Strategy in the First Health State (Prior)
eFigure 48. Convergence Diagnostics for the Output of JAGS Related to the Cost Parameter of the Second-Line WGS Strategy in the Third Health State (Prior)
eFigure 49. Convergence Diagnostics for the Output of JAGS Related to the Cost Parameter of the Second-Line WGS Strategy in the Fourth Health State (Prior)
eFigure 50. Convergence Diagnostics for the Output of JAGS Related to the Cost Parameter of the Second-Line WGS Strategy in the Fifth Health State (Prior)
eFigure 51. Convergence Diagnostics for the Output of JAGS Related to the Cost Parameter of the Second-Line WGS Strategy in the Eighth Health State (Prior)
eFigure 52. Convergence Diagnostics for the Output of JAGS Related to the Cost Parameter of the WES Strategy in the First Health State (Prior)
eFigure 53. Convergence Diagnostics for the Output of JAGS Related to the Cost Parameter of the WES Strategy in the Second Health State (Prior)
eFigure 54. Convergence Diagnostics for the Output of JAGS Related to the Cost Parameter of the WES Strategy in the Third Health State (Prior)
eFigure 55. Convergence Diagnostics for the Output of JAGS Related to the Cost Parameter of the WES Strategy in the Eighth Health State (Prior)
eFigure 56. Convergence Diagnostics for the Output of JAGS Related to the Cost Parameter of the WGS Strategy in the First Health State (Prior)
eFigure 57. Convergence Diagnostics for the Output of JAGS Related to the Cost Parameter of the WGS Strategy in the Second Health State (Prior)
eFigure 58. Convergence Diagnostics for the Output of JAGS Related to the Cost Parameter of the WGS Strategy in the Eighth Health State (Prior)
eTable 1. Robustness Analysis Results Over Lifetime Horizon
eTable 2. Summary Statistics of the Marginal Posterior Distribution for Each of the Model Parameters

This supplementary material has been provided by the authors to give readers additional information about their work.

© 2024 Nurchis MC et al. *JAMA Network Open*.

eMethods. Type of Model and Model Analysis

Type of model

Following the Bayes theorem:

$$p(\theta|D) = \frac{p(D|\theta)p(\theta)}{p(D)}$$

$p(\theta|D)$ represents the posterior distribution, showing the plausibility of parameters considering the present data (D); $p(\theta)$ acts as the prior for the parameter θ, indicating the credibility of θ values before the data (D) observation; $p(D|\theta)$ is referred to as the likelihood, representing the probability of the data (D) being produced by a model with a specific parameter value θ; $p(D)$ is known as evidence (or marginal likelihood), and reflects the cumulative probability of the data as per the model, calculated by averaging over all potential parameters values, weighted by the confidence in those parameters.

Typically, scholars have insights about the likelihood and prior distributions, and aim to approximate the posterior distribution, which signifies an alteration of prior assumptions about the parameters' reliability, in the presence of new data. An issue with this method is that determining the posterior distribution of θ necessitates the calculation of the marginal likelihood, which for continuous variables equates to estimate an extremely complex integral, potentially unsolvable analytically. A feasible resolution to this issue is the adoption of the Markov Chain Monte Carlo (MCMC), essentially a class of algorithms designed to sample from an unknown probability distribution. To approximate the desired posterior distribution, the MCMC method needs only knowledge of the prior and the likelihood probability density functions, bypassing the need to evaluate the complex integral present in the denominator of the Bayes' equation.

For the present study, the widely recognized Gibbs algorithm was chosen to derive an approximation of the posterior distribution $p(\theta|D)$, by generating a substantial sample of θ values. Implementing the MCMC method with an infinite number of simulations $S \rightarrow \infty$ ensures that the simulated posterior distribution converges to the actual one with probability

© 2024 Nurchis MC et al. *JAMA Network Open*.

one. In this perspective, diagnostic procedures are required to investigate and evaluate the representativeness, accuracy, and efficacy of the MCMC process. Graphical evidence employing standard diagnostic tools such as the trace plot, the density plot, the Gelman-Rubin statistics, and the chain's autocorrelation functions was presented.

The trace plot and Gelman-Rubin statistics facilitate the analysis of the MCMC's representativeness and convergence. The trace plot displays several superimposed chains, whose overlap would suggest a representation of the same posterior distribution. The Gelman-Rubin statistic provides a quantitative examination of the convergence of multiple chains by comparing intra-chain variability with inter-chain variability; if the chains have converged, the intra-chain variability would closely resemble the inter-chain variability, resulting in a value close to 1. A value exceeding 1 indicates a convergence deficiency, suggesting that the chains have not sufficiently explored the target distribution. MCMC accuracy was assessed through an autocorrelation measure that provides the Effective Sample Size (ESS). The ESS is a measure that takes into consideration the autocorrelation within the MCMC chain and offers an estimate of the effective quantity of independent samples, pointing out the number of uncorrelated samples that would hold the same level of information as the original autocorrelated samples. An ESS reaching 10,000 is generally recommended for stable 95% higher density intervals.

Model analysis

The findings were illustrated using a cost-effectiveness plane (CEP), cost-effectiveness acceptability curve (CEAC), and the cost-effectiveness acceptability frontier (CEAF), as advocated by the International Society for Pharmacoeconomics and Outcomes Research (ISPOR) guideline [36] and the Second Panel on Cost-effectiveness in Health and Medicine [37]. The CEAC shows the likelihood that each intervention would be deemed the optimal choice at various thresholds, while the CEAF depicts the net monetary benefit at each willingness-to-pay threshold and the degree of uncertainty around the optimal choice. Expected incremental

© 2024 Nurchis MC et al. *JAMA Network Open*.

benefits (EIB) were also estimated for WGS compared to the other testing strategies. In addition, calculations for the Expected Value of Perfect Information (EVPI) and the Expected Value of Partial Perfect Information (EVPPI) were performed to assess the value of collecting additional data[38].

The EIB indicates the expected average incremental benefit by computing the average incremental benefit of each simulation. The incremental benefit function can be modelled as a function of the willingness to pay (WTP) k:

$$IB(\theta) = k\Delta_e - \Delta_c$$

The following approximates the INB by adopting the set of posterior samples S:

$$\frac{1}{S}\sum_{s}^{S} IB(\theta_s)$$

in the context of the s-th simulation, θ_s represents the actual configuration of the parameters θ. For the intervention of interest to be deemed cost-effective, the EIB should exceed 0, considering the WTP.

The EVPI is given, for each level of the threshold, by the difference between the expected value with perfect information and the expected value with the existing information.

This method allows to quantify the uncertainty related to the model of the cost-effectiveness analysis into a quantifiable economic measure and it is based on the opportunity loss (OL) concept. The OL is given by:

$$OL = U_* - U_\tau$$

© 2024 Nurchis MC et al. *JAMA Network Open*.

where U_* is the utility level linked to the best intervention in simulation S, while U_τ is the utility level linked to the average preferred intervention in simulation S. For the present study, EVPI was used to compute the value of additional research needed to decrease uncertainty in the cost-effectiveness analysis of WGS against other testing strategies.

In addition, the EVPPI was estimated to establish the value of reducing uncertainty in specific model parameters, and to discern which parameters have the highest uncertainty, thereby steering further research in those areas needing more information.

Lastly, an information-rank plot was also depicted. For each parameter and level of the WTP threshold, a bar chart is plotted to illustrate the ratio of EVPPI to EVPI, indicating the contribution of each parameter in relation to the value of information.

eResults. Information Analysis, Robustness Analysis, and Diagnostic Performance

Value of information analysis

The EVPPI for the transition probabilities, costs and effectiveness accounted for €248,205 or US$269,923, €264,541 or US$287,688, and €483,828 or US$477,225, respectively (eFigures 8-10). In eFigure 11 this parameter demonstrated the highest ratio, indicating the magnitude of the expected value in acquiring additional information.

Robustness analysis including costs of change in clinical management

The robustness analysis included also costs for change in clinical management, for each diagnostic pathway. The findings confirmed that, considering the chosen threshold, using first-line WGS would be a cost-effective strategy either compared to SOC, first-line WES, second-line WES, or second-line WGS, yielding an ICER of €38,730 or US$42,119 (95% CI €36,025–€39,490), €34,600 or US37,628 (95% CI €32,373–€44,045), €36,473 or US$39,664 (95% CI €34,172–€37,893), and €21,232 or US$23,090 (95% CI €20,652–€23,845) per diagnosis,

respectively. eTable 1 lists the overall results of the robustness analysis for the simulated cohort.

Diagnostic performance for MCMC simulation

The MCMC performance was evaluated according to proper diagnostic measures. eTable 2 shows the summary statistics for all the parameters used in the economic analysis. For the costs' subset of parameters, eFigures 12 to 58 illustrates the trace plots, the ESS, the Gelman-Rubin statistics, and the density plots. The trace plots demonstrated consistent and overlapping patterns for the sampled parameters, implying convergence. As indicated by the smooth density plots, most of the investigated parameters had unimodal distributions, suggesting that posterior distributions were orderly and behaved as expected (eFigures 12 to 58). As listed in eTable 1 and shown in eFigures 12 to 58, for the parameters' subset of interest, the Gelman-Rubin statistics revealed that R-hat values were approximating 1 for each parameter, strengthening the evidence for chain convergence.

Additionally, the ESS displayed values larger than 10,000 in relation to the overall number of samples for all the parameters, pointing to proficient sampling and a dependable estimation of the posterior distribution (eTable 1 and eFigures 12 to 58).

© 2024 Nurchis MC et al. *JAMA Network Open*.

eFigure 1. Contour Plot for WGS vs SOC

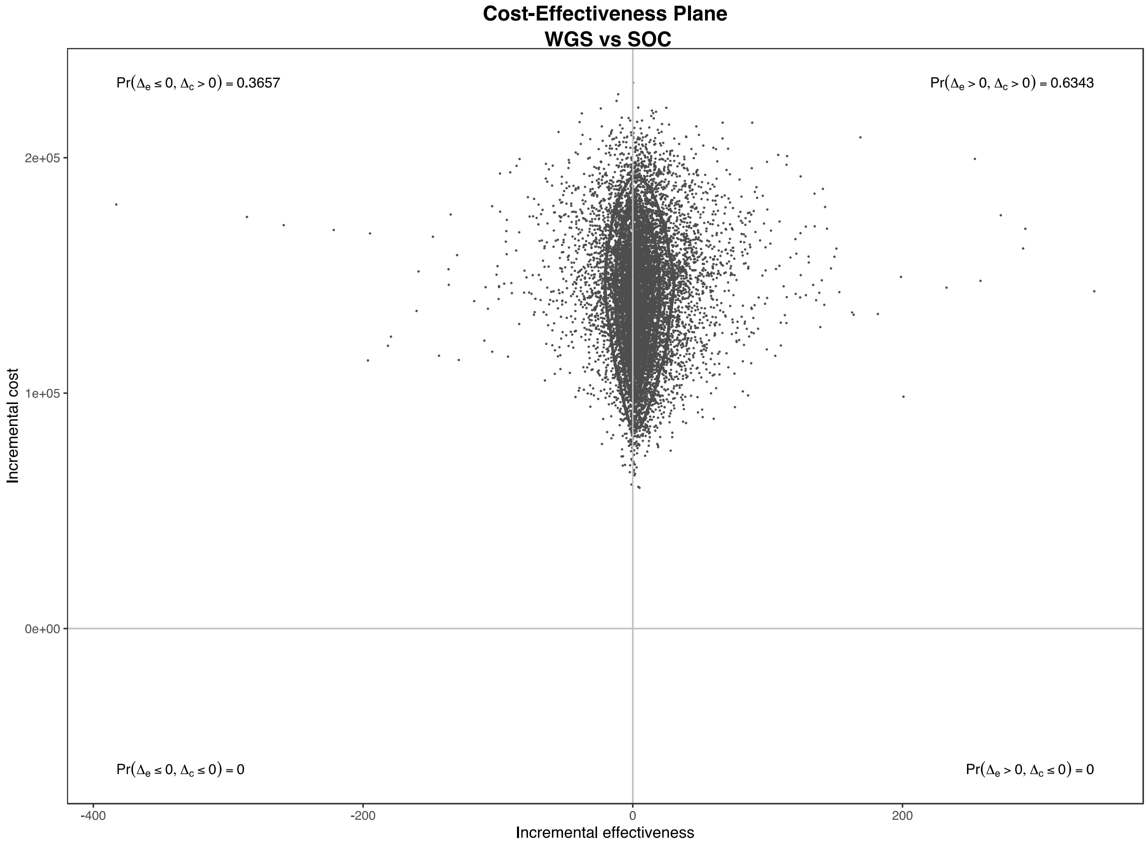

© 2024 Nurchis MC et al. *JAMA Network Open*.

eFigure 2. Contour Plot for WGS vs WES

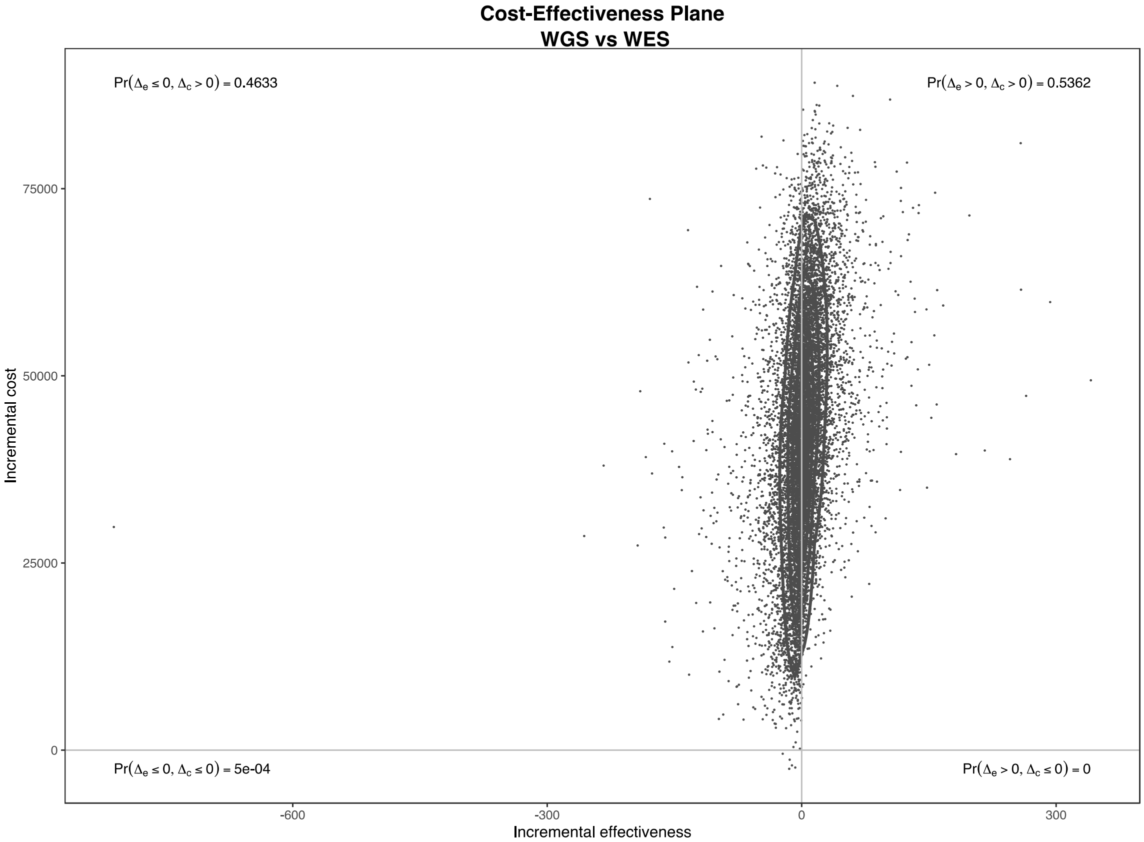

eFigure 3. Contour Plot for WGS vs Second-Line WES

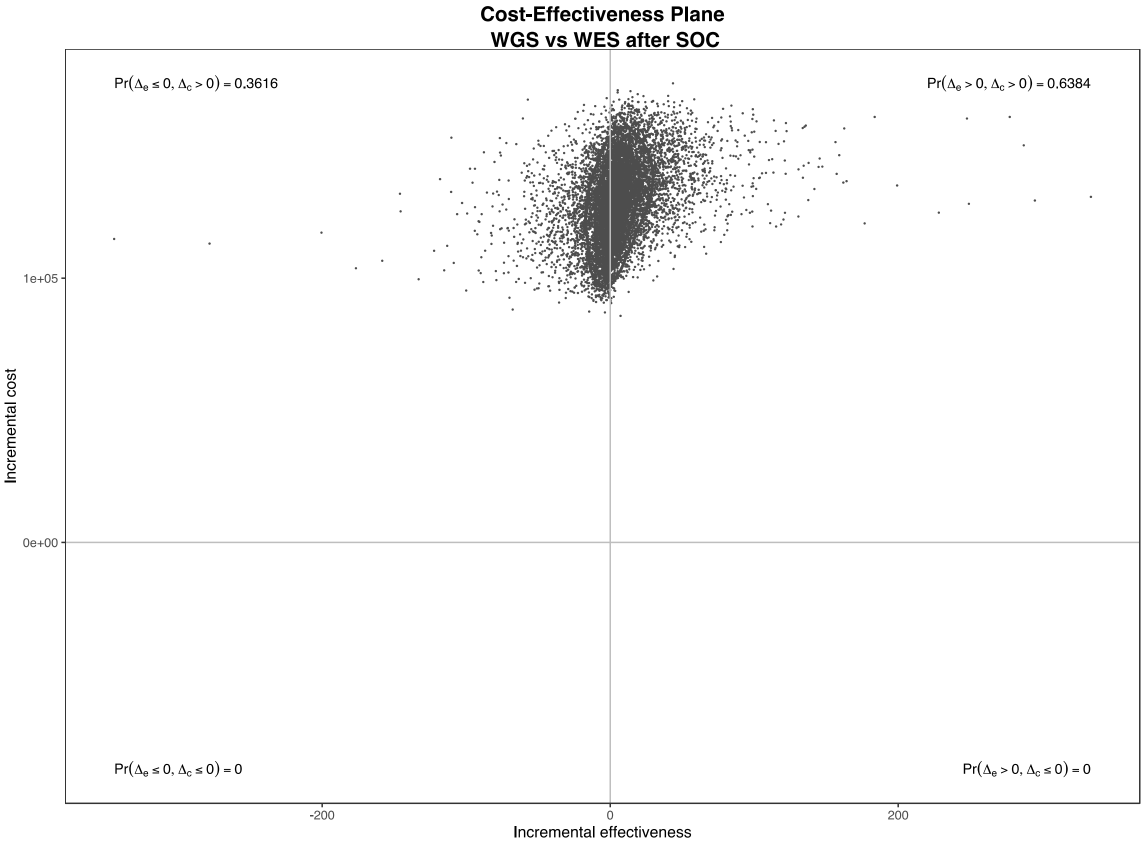

© 2024 Nurchis MC et al. *JAMA Network Open*.

eFigure 4. Contour Plot for WGS vs Second-Line WGS

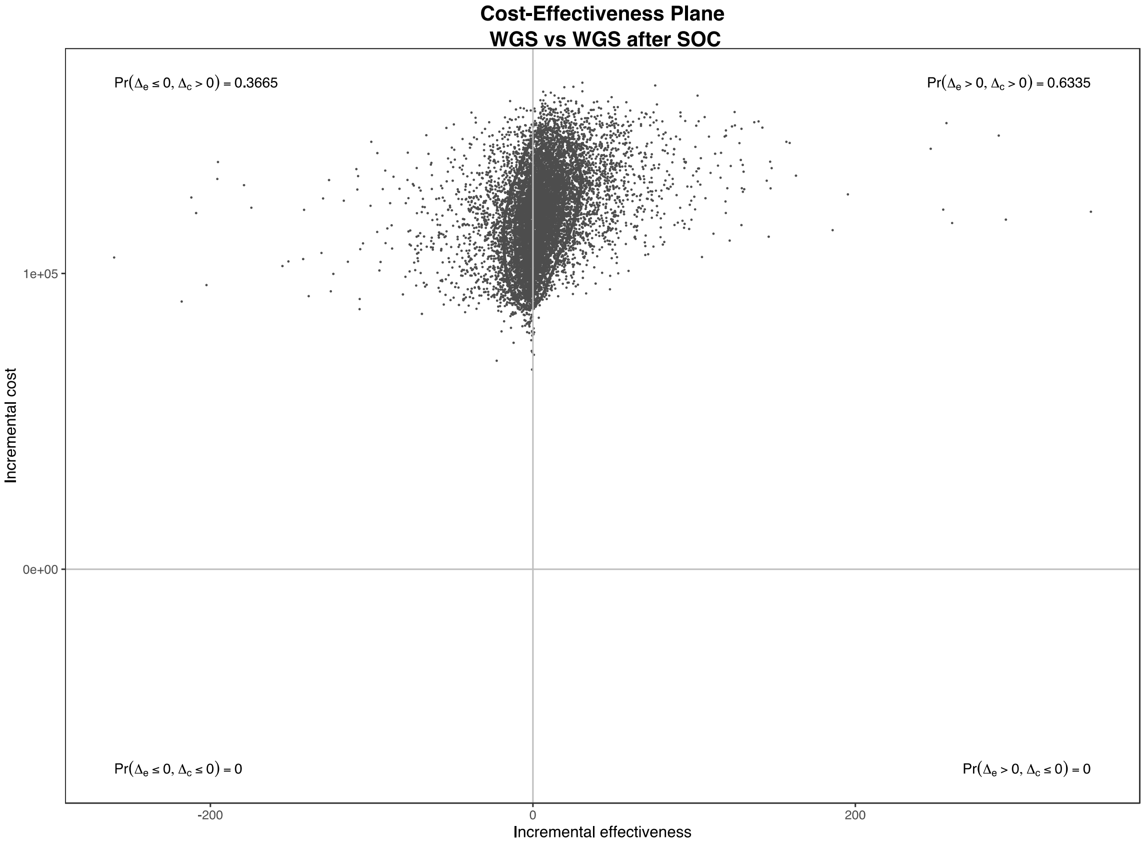

eFigure 5. Cost-Effectiveness Acceptability Curve (CEAC)

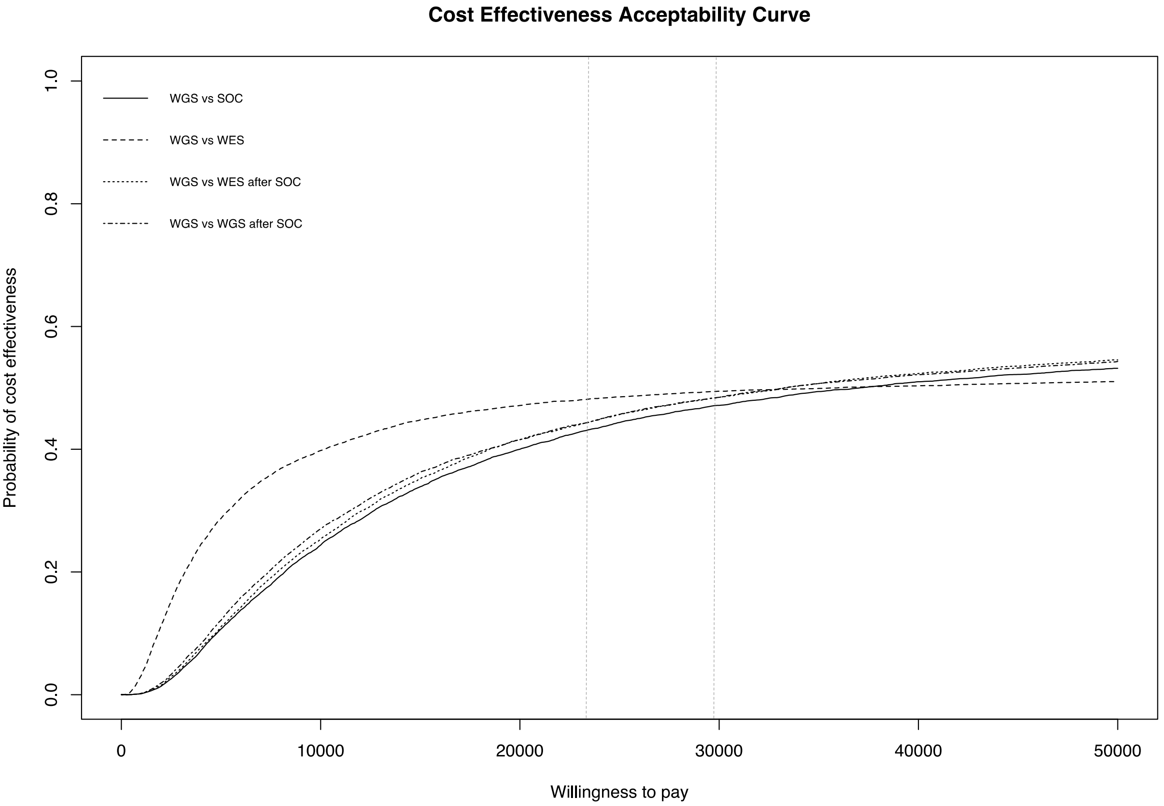

© 2024 Nurchis MC et al. *JAMA Network Open*.

eFigure 6. Cost-Effectiveness Acceptability Frontier Curve (CEAF)

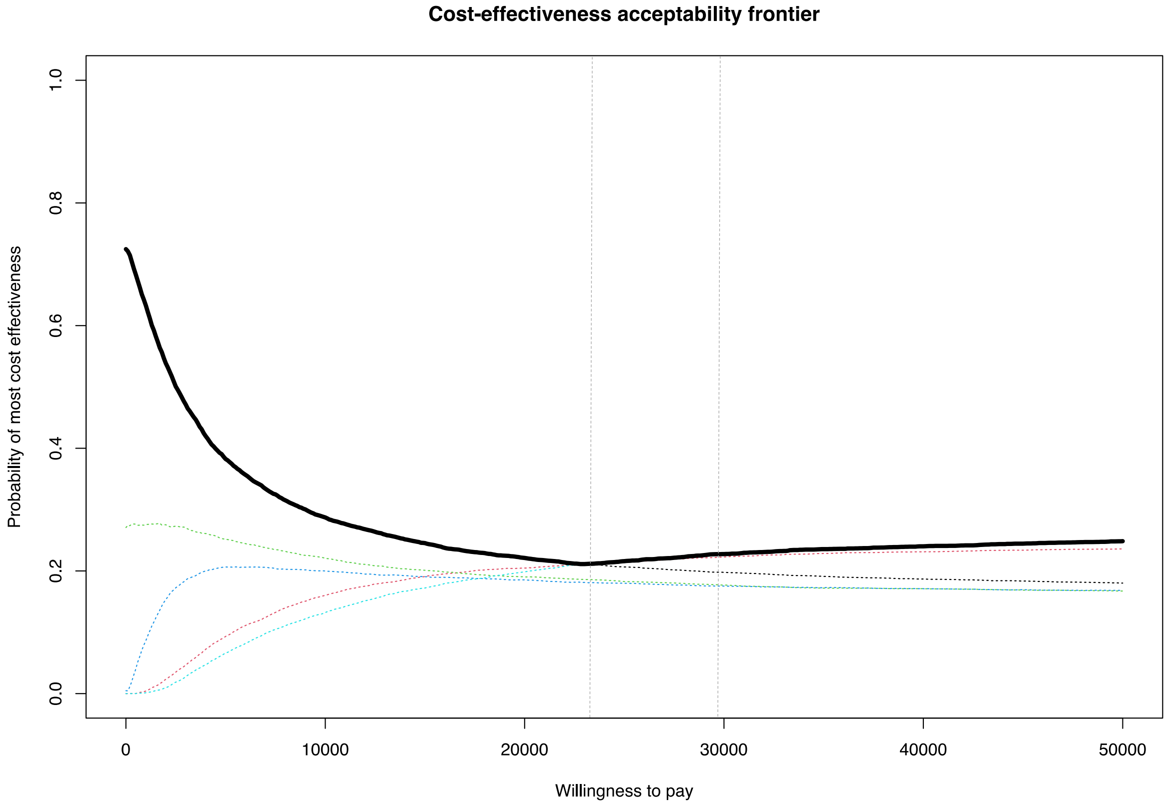

eFigure 7. Expected Incremental Benefit (EIB) for Each Testing Strategy

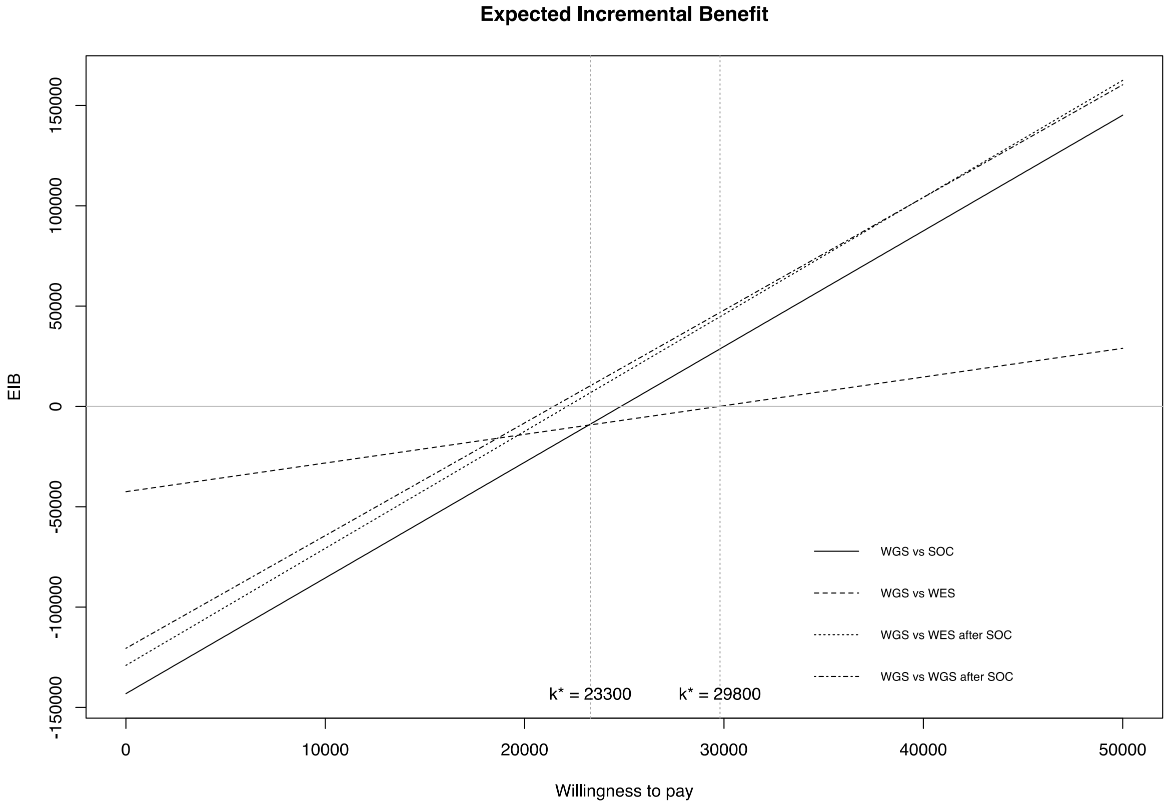

© 2024 Nurchis MC et al. *JAMA Network Open*.

eFigure 8. Population Expected Value of Partially Perfect Information (EVPPI) Curve for Transition Probabilities

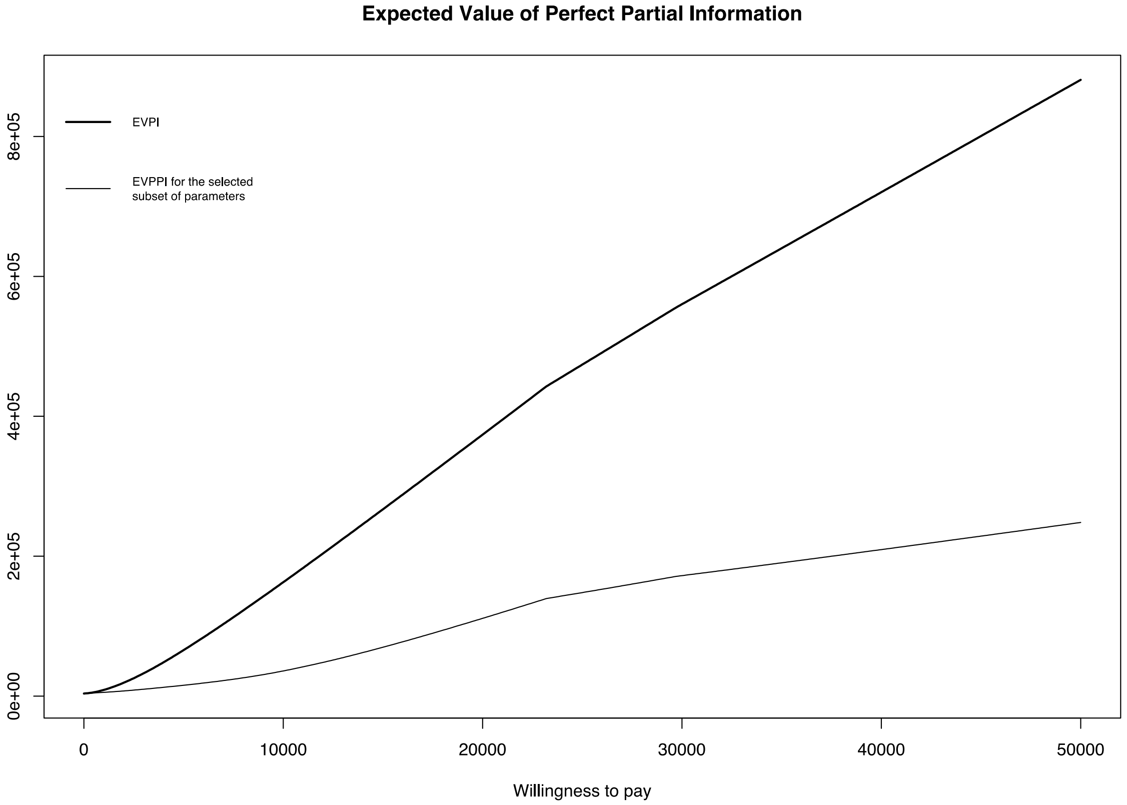

eFigure 9. Population Expected Value of Partially Perfect Information (EVPPI) Curve for Costs

© 2024 Nurchis MC et al. *JAMA Network Open*.

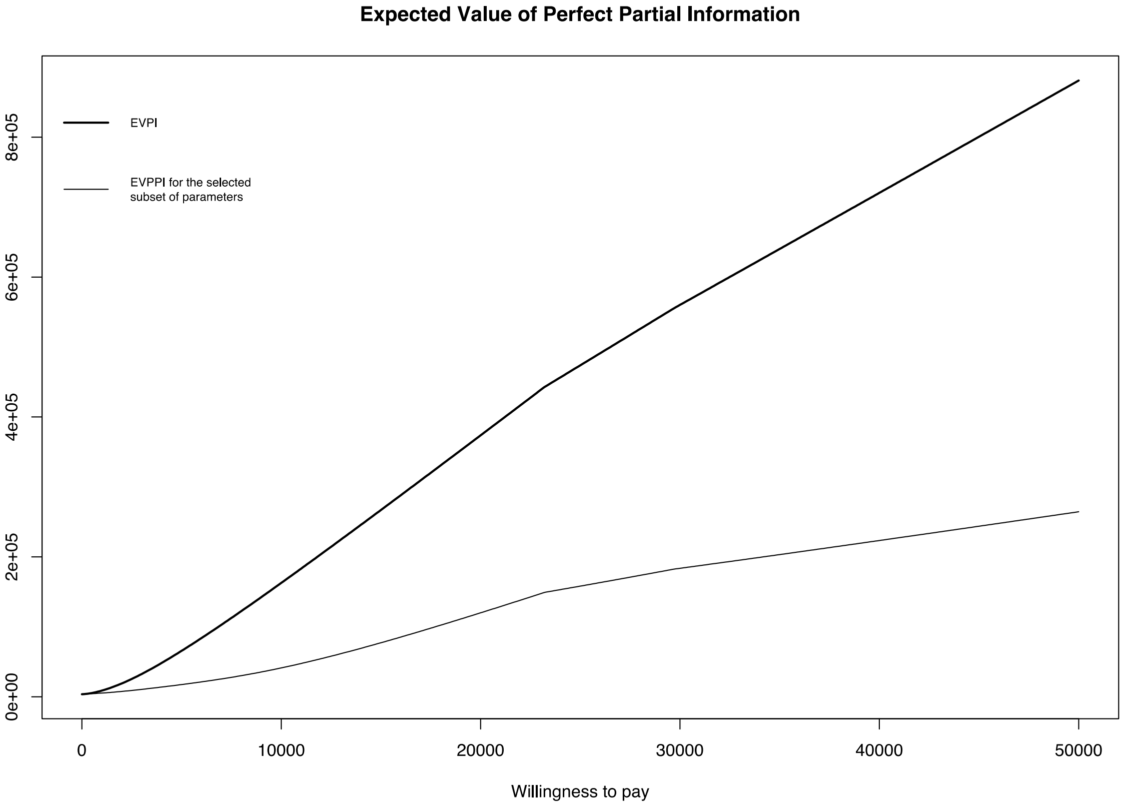

eFigure 10. Population Expected Value of Partially Perfect Information (EVPPI) Curve for Effectiveness Measure (ie, Diagnostic Yield)

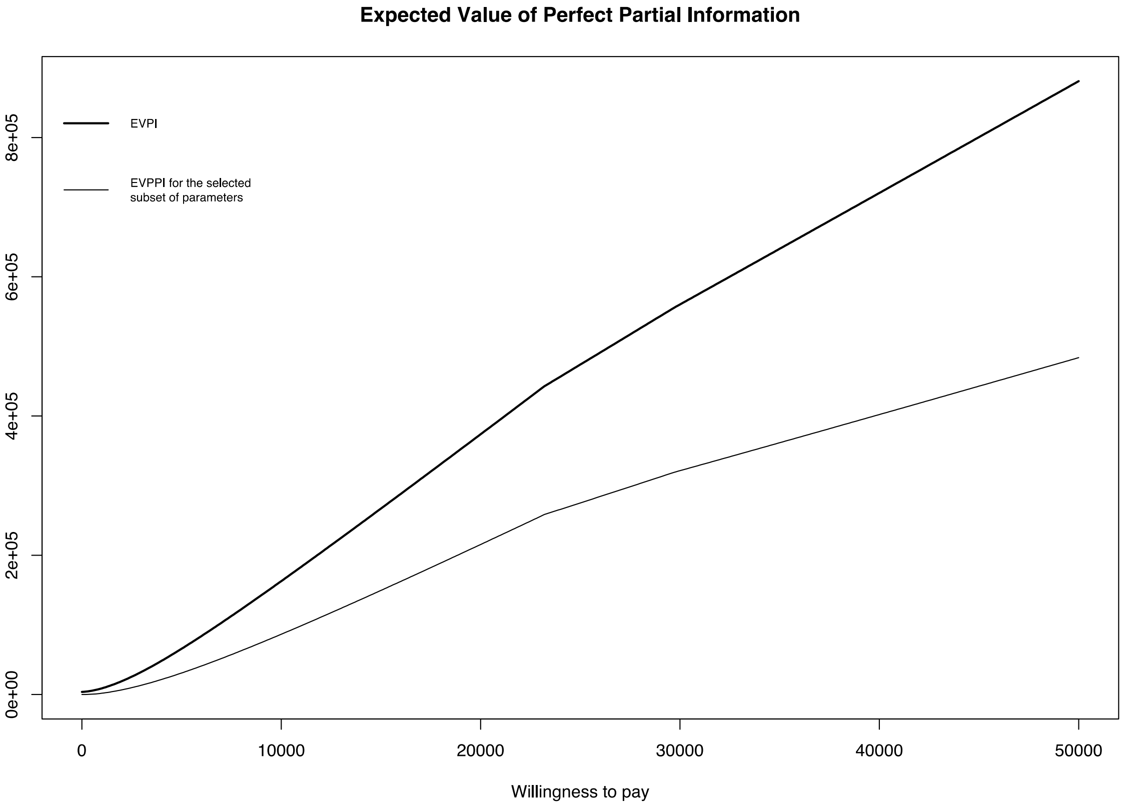

© 2024 Nurchis MC et al. *JAMA Network Open*.

eFigure 11. Information-Rank Plot Reporting a Ranking of the Model Parameters in Terms of Their Impact on the Expected Value of Information

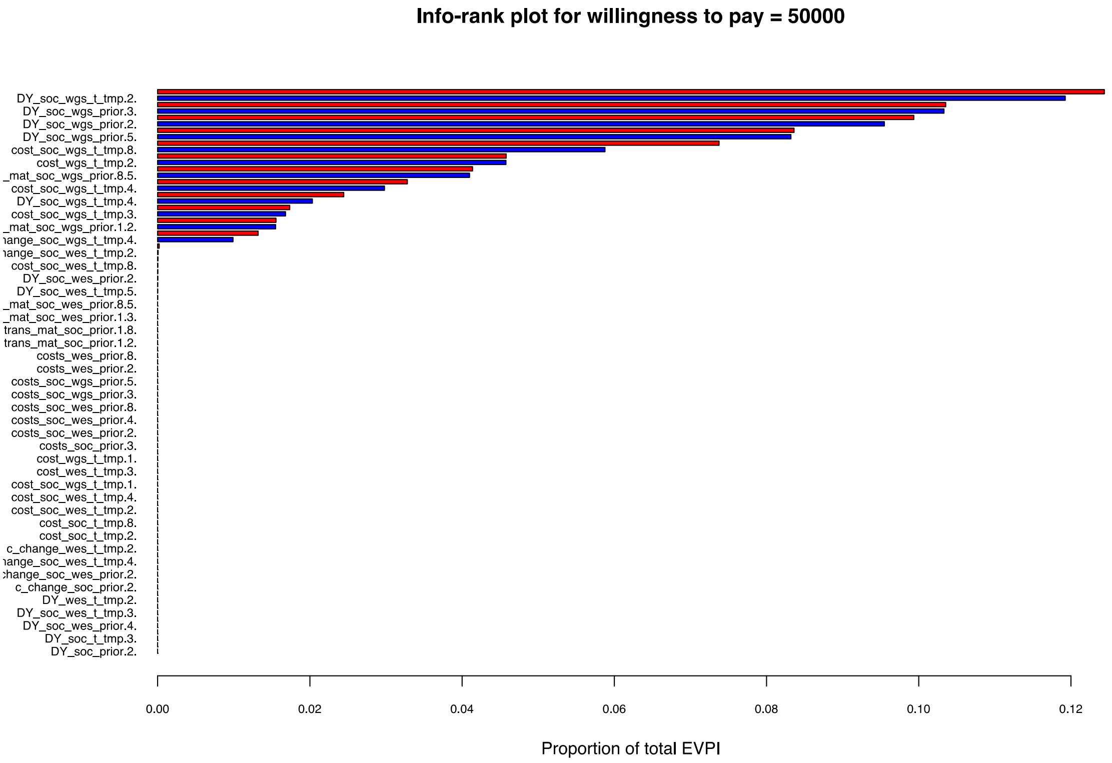

© 2024 Nurchis MC et al. *JAMA Network Open*.

eFigure 12. Convergence Diagnostics for the Output of JAGS Related to the Cost Parameter of the SOC Strategy in the First Health State

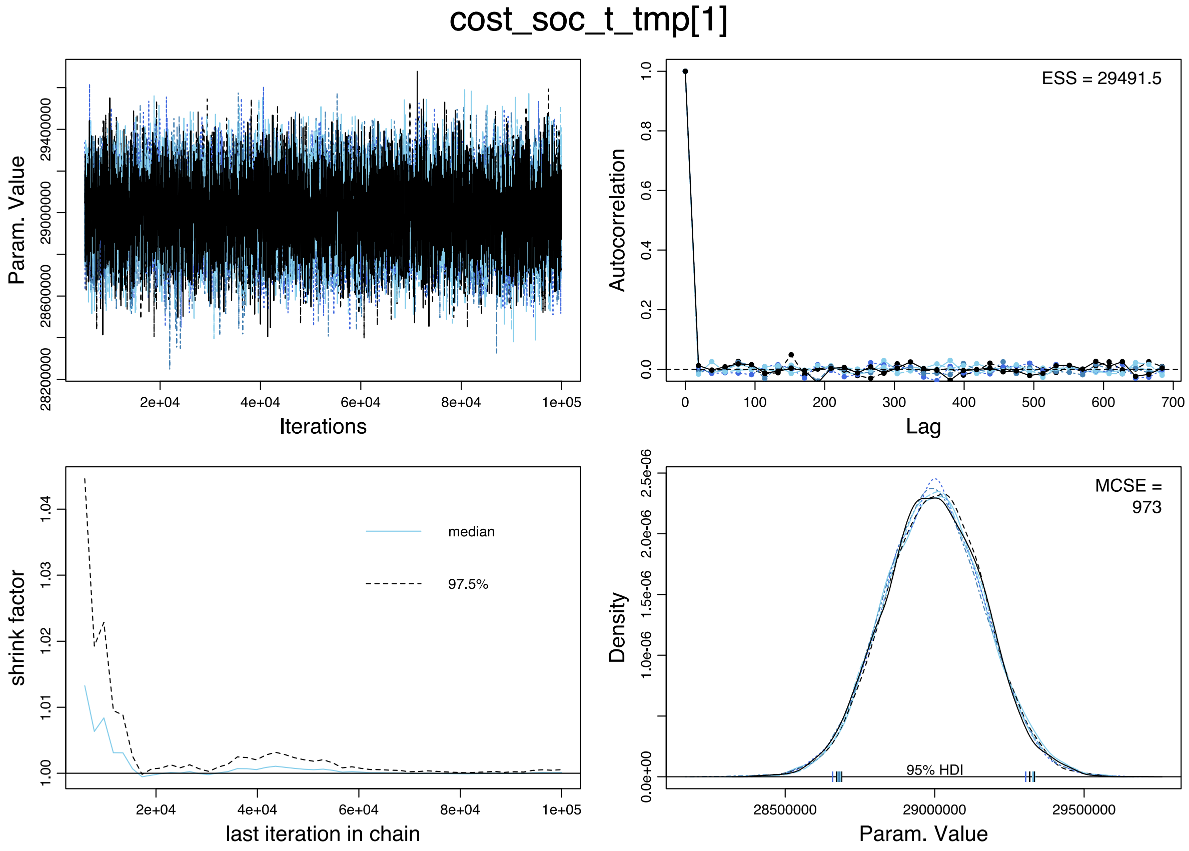

© 2024 Nurchis MC et al. *JAMA Network Open*.

eFigure 13. Convergence Diagnostics for the Output of JAGS Related to the Cost Parameter of the SOC Strategy in the Second Health State

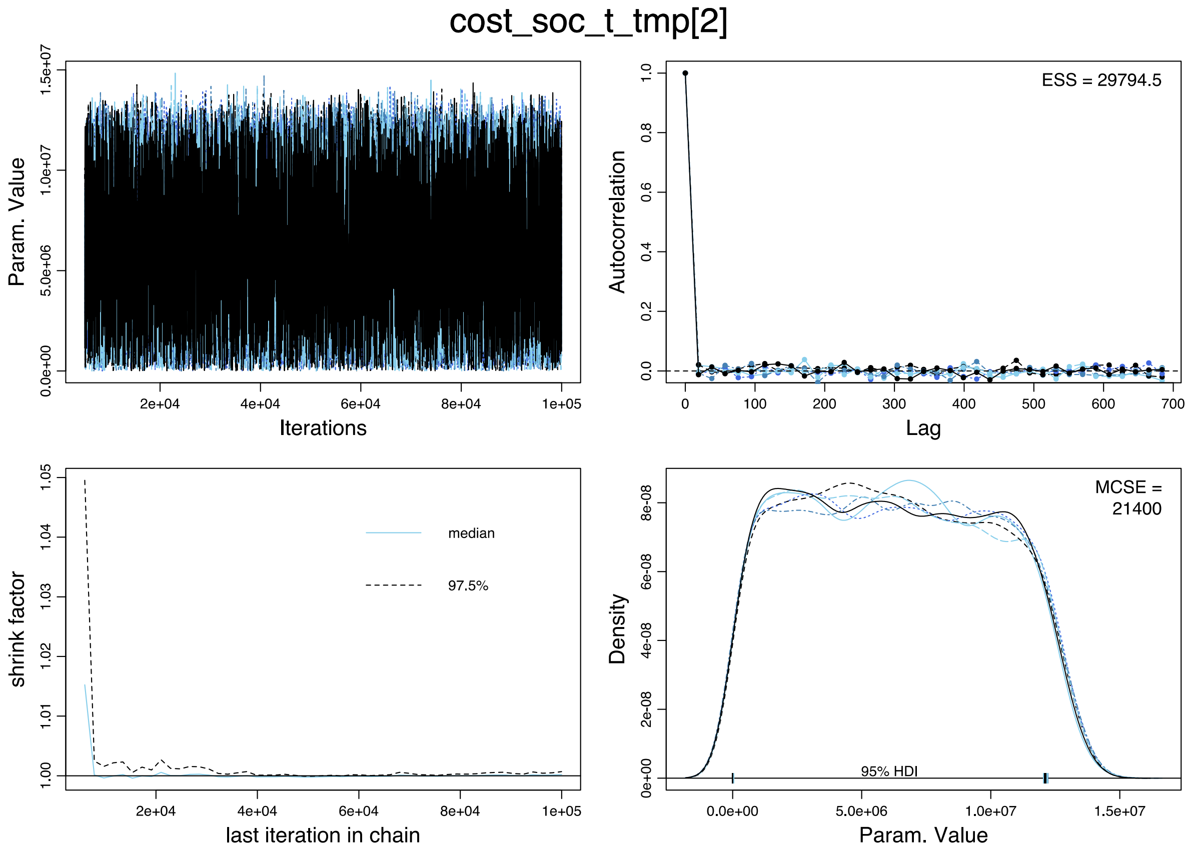

© 2024 Nurchis MC et al. *JAMA Network Open*.

eFigure 14. Convergence Diagnostics for the Output of JAGS Related to the Cost Parameter of the SOC Strategy in the Third Health State

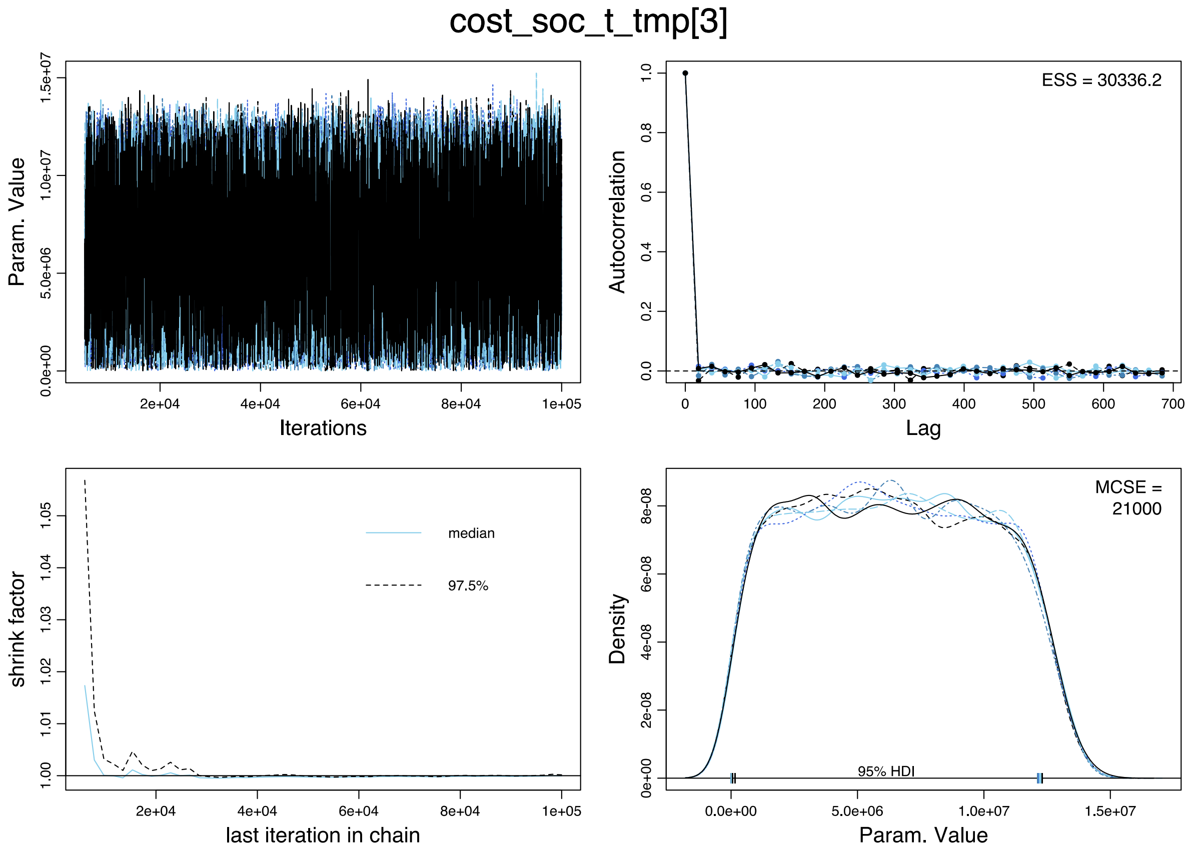

© 2024 Nurchis MC et al. *JAMA Network Open*.

eFigure 15. Convergence Diagnostics for the Output of JAGS Related to the Cost Parameter of the SOC Strategy in the Eighth Health State

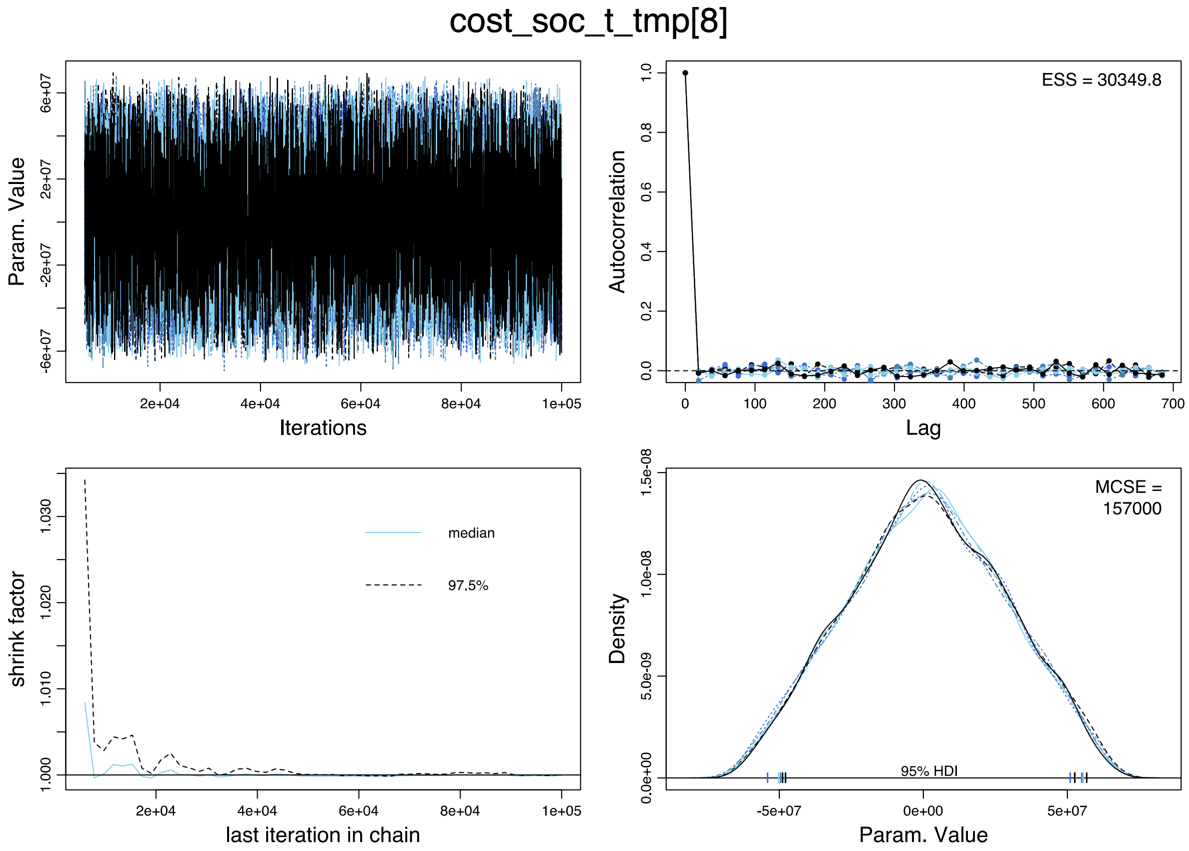

© 2024 Nurchis MC et al. *JAMA Network Open*.

eFigure 16. Convergence Diagnostics for the Output of JAGS Related to the Cost Parameter of the Second-Line WES Strategy in the First Health State

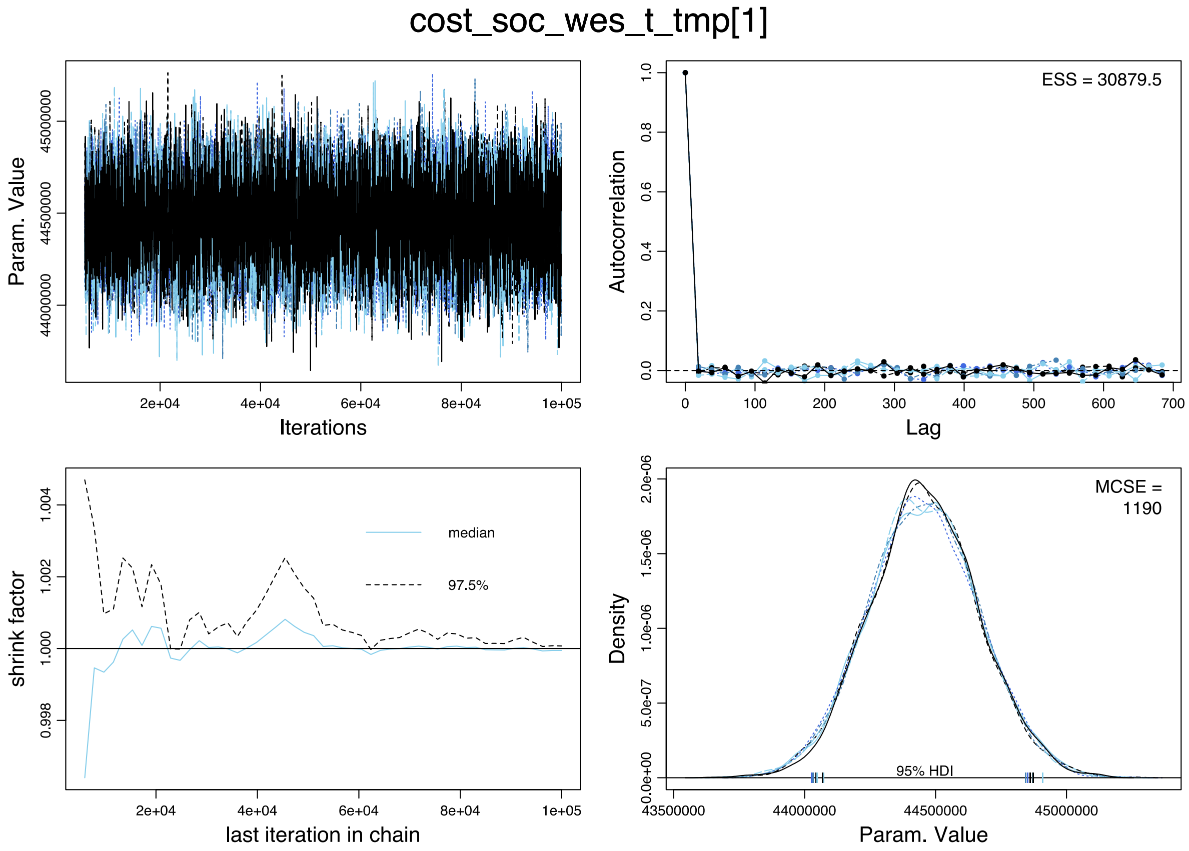

© 2024 Nurchis MC et al. *JAMA Network Open*.

eFigure 17. Convergence Diagnostics for the Output of JAGS Related to the Cost Parameter of the Second-Line WES Strategy in the Second Health State

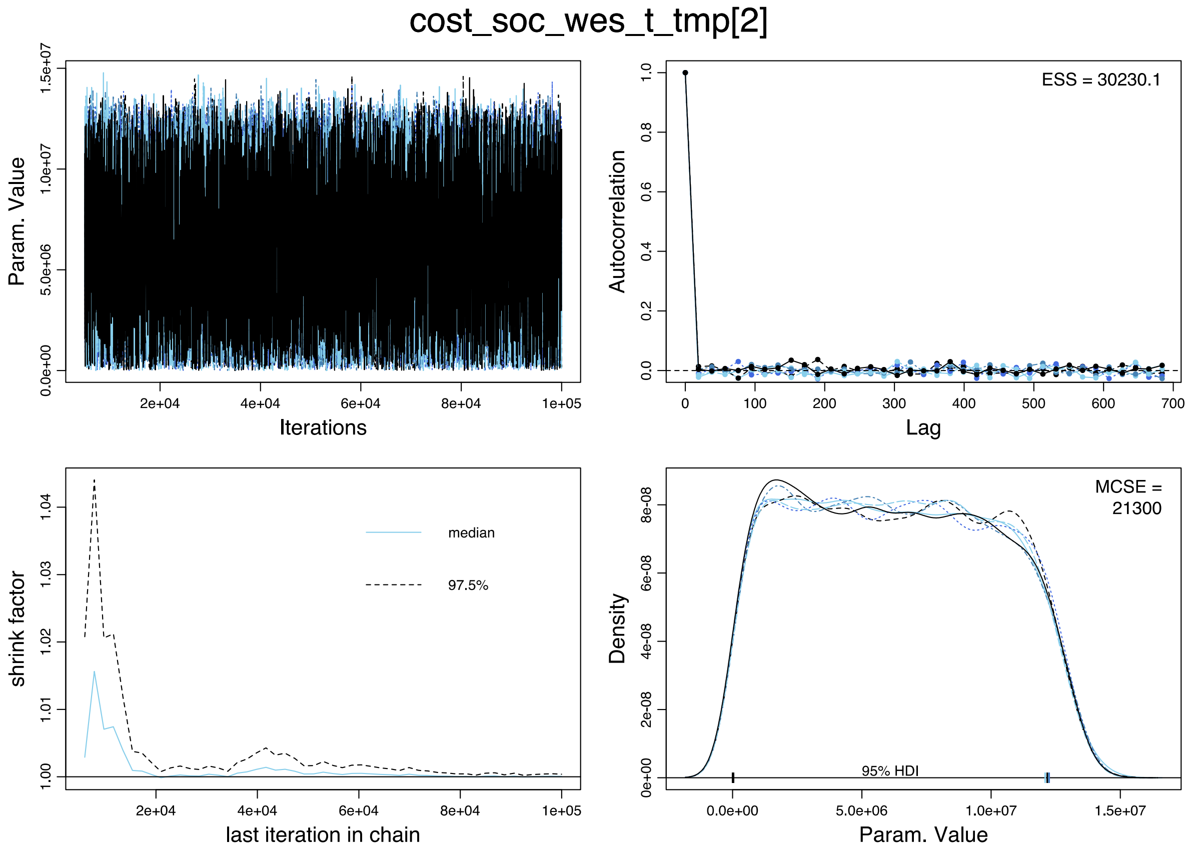

© 2024 Nurchis MC et al. *JAMA Network Open*.

eFigure 18. Convergence Diagnostics for the Output of JAGS Related to the Cost Parameter of the Second-Line WES Strategy in the Third Health State

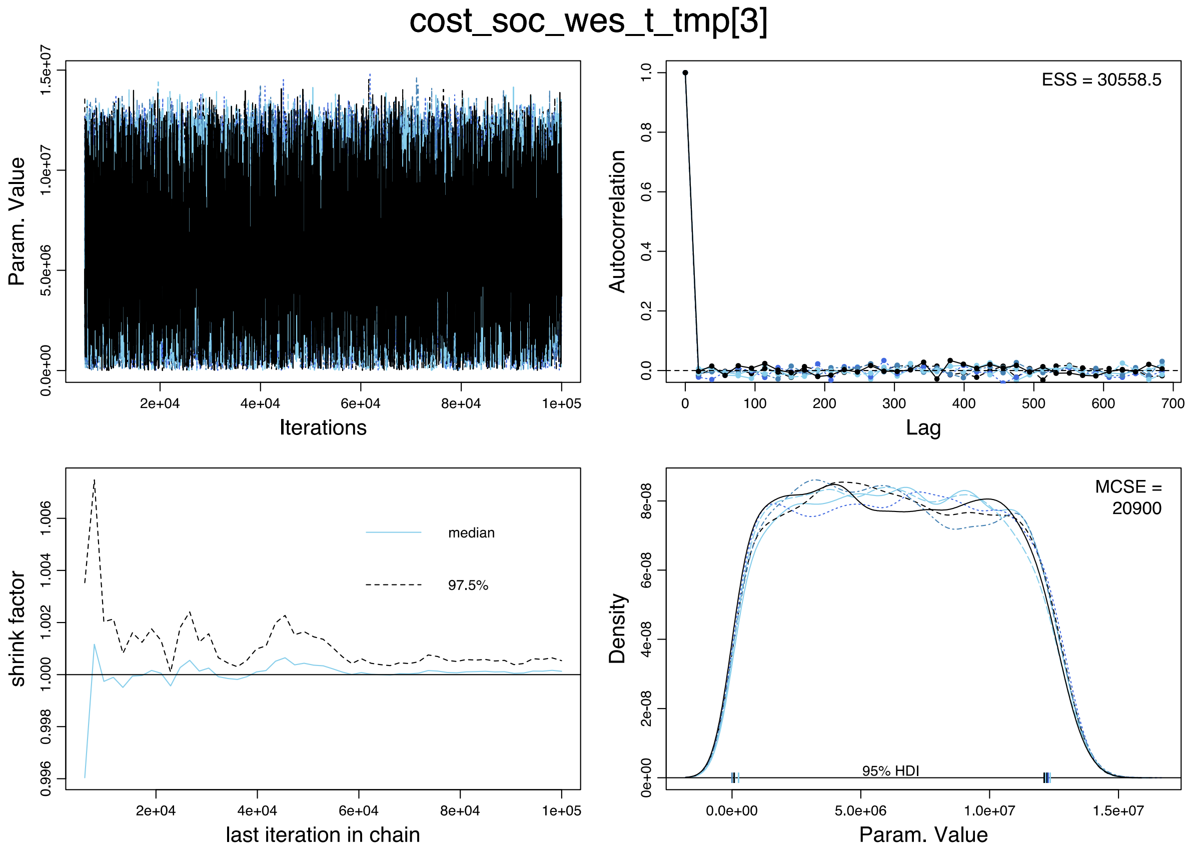

© 2024 Nurchis MC et al. *JAMA Network Open*.

eFigure 19. Convergence Diagnostics for the Output of JAGS Related to the Cost Parameter of the Second-Line WES Strategy in the Fourth Health State

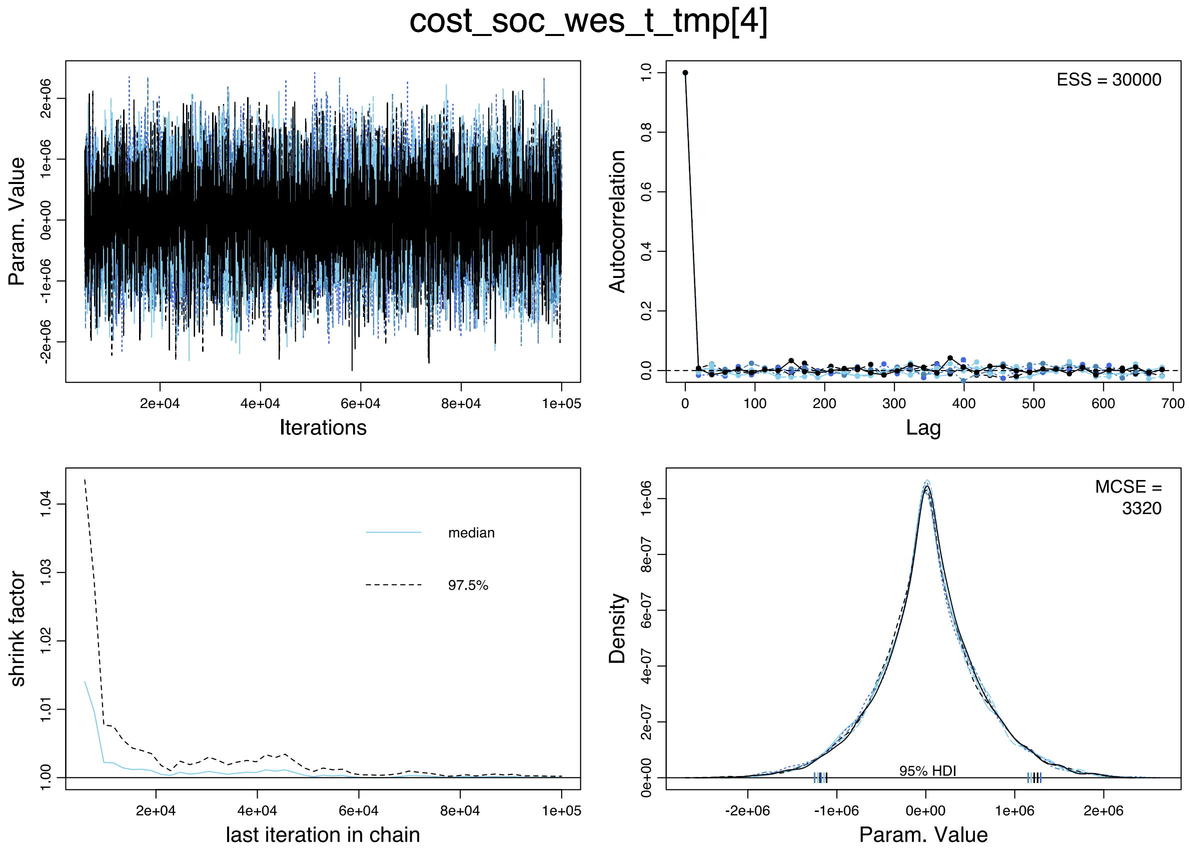

© 2024 Nurchis MC et al. *JAMA Network Open*.

eFigure 20. Convergence Diagnostics for the Output of JAGS Related to the Cost Parameter of the Second-Line WES Strategy in the Fifth Health State

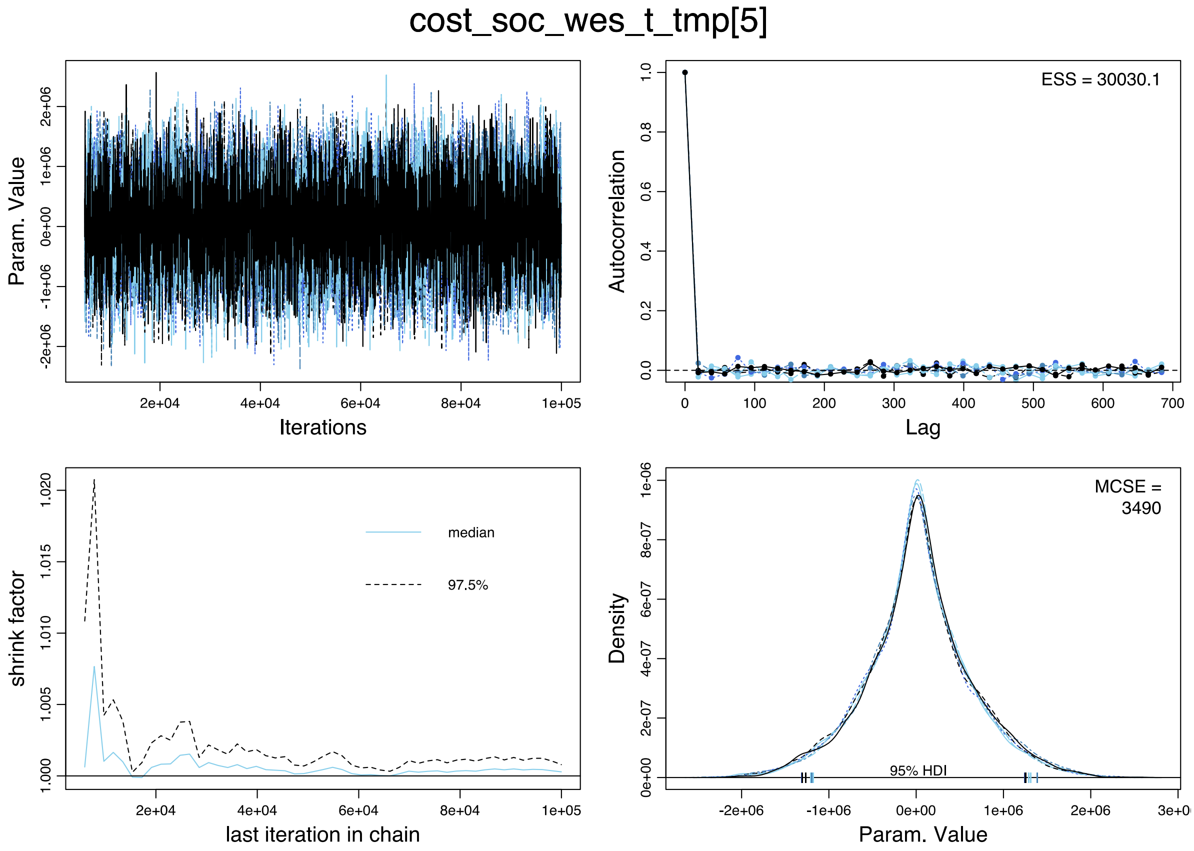

© 2024 Nurchis MC et al. *JAMA Network Open*.

eFigure 21. Convergence Diagnostics for the Output of JAGS Related to the Cost Parameter of the Second-Line WES Strategy in the Eighth Health State

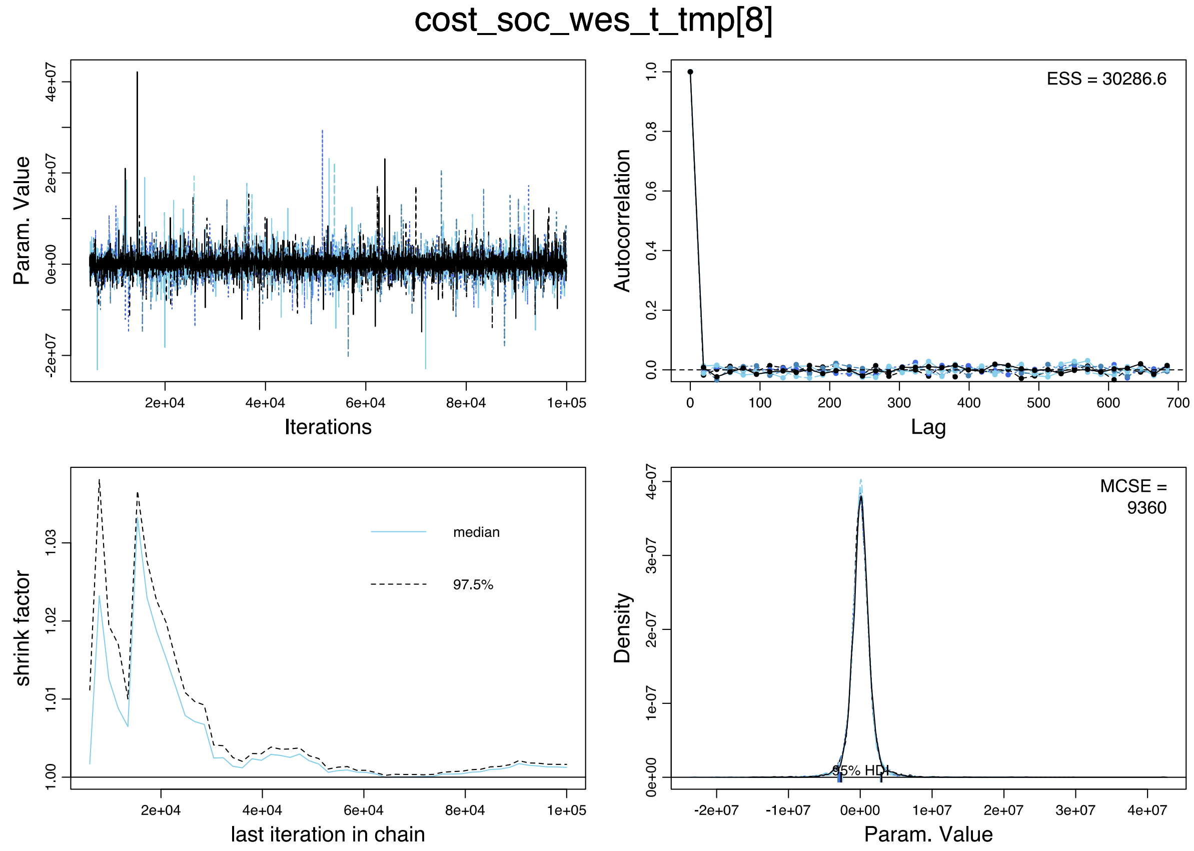

© 2024 Nurchis MC et al. *JAMA Network Open*.

eFigure 22. Convergence Diagnostics for the Output of JAGS Related to the Cost Parameter of the Second-Line WGS Strategy in the First Health State

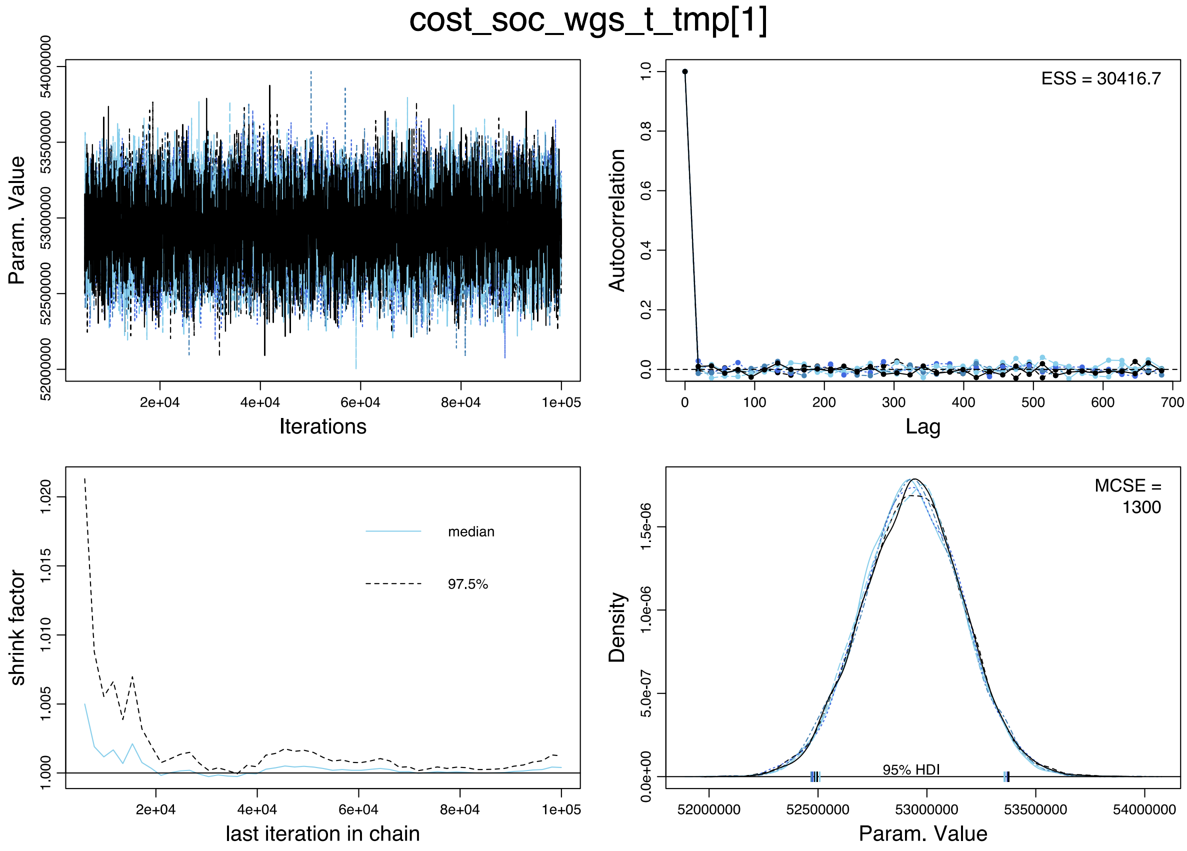

© 2024 Nurchis MC et al. *JAMA Network Open*.

eFigure 23. Convergence Diagnostics for the Output of JAGS Related to the Cost Parameter of the Second-Line WGS Strategy in the Second Health State

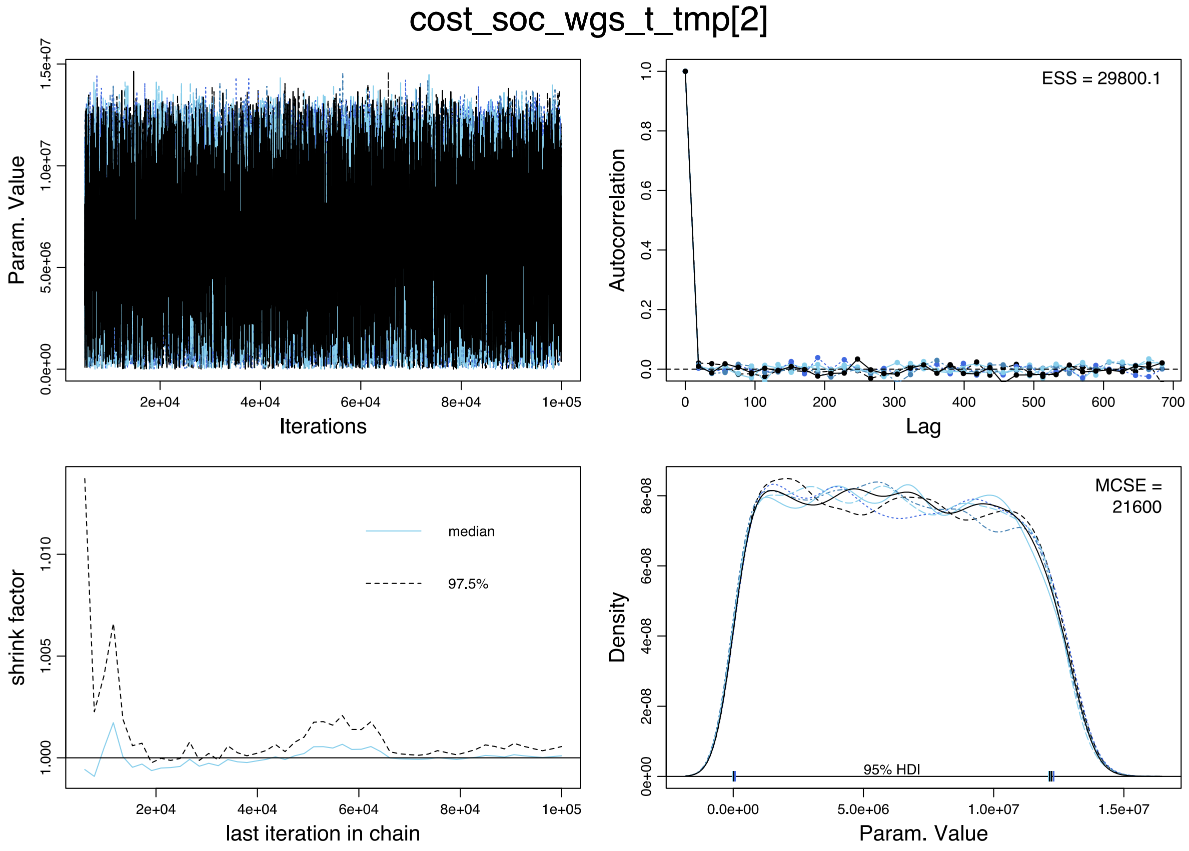

© 2024 Nurchis MC et al. *JAMA Network Open*.

eFigure 24. Convergence Diagnostics for the Output of JAGS Related to the Cost Parameter of the Second-Line WGS Strategy in the Third Health State

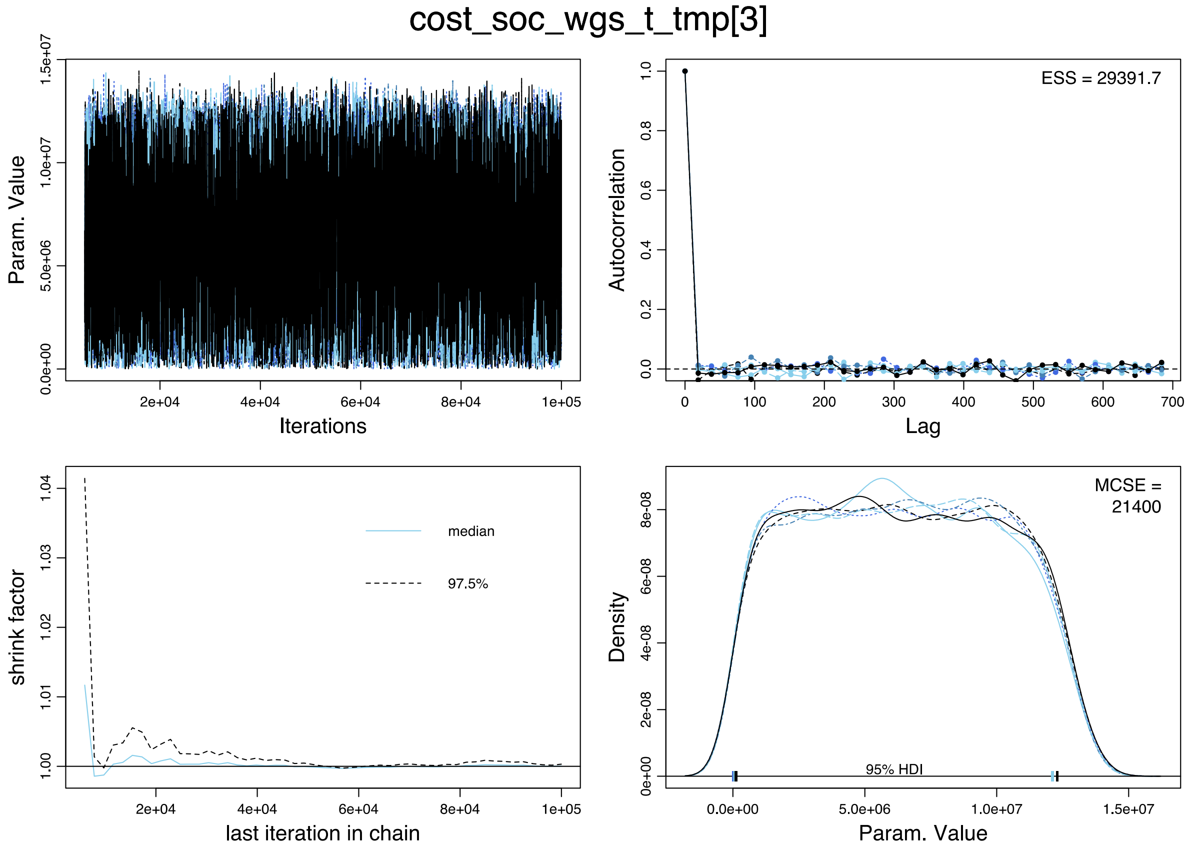

© 2024 Nurchis MC et al. *JAMA Network Open*.

eFigure 25. Convergence Diagnostics for the Output of JAGS Related to the Cost Parameter of the Second-Line WGS Strategy in the Fourth Health State

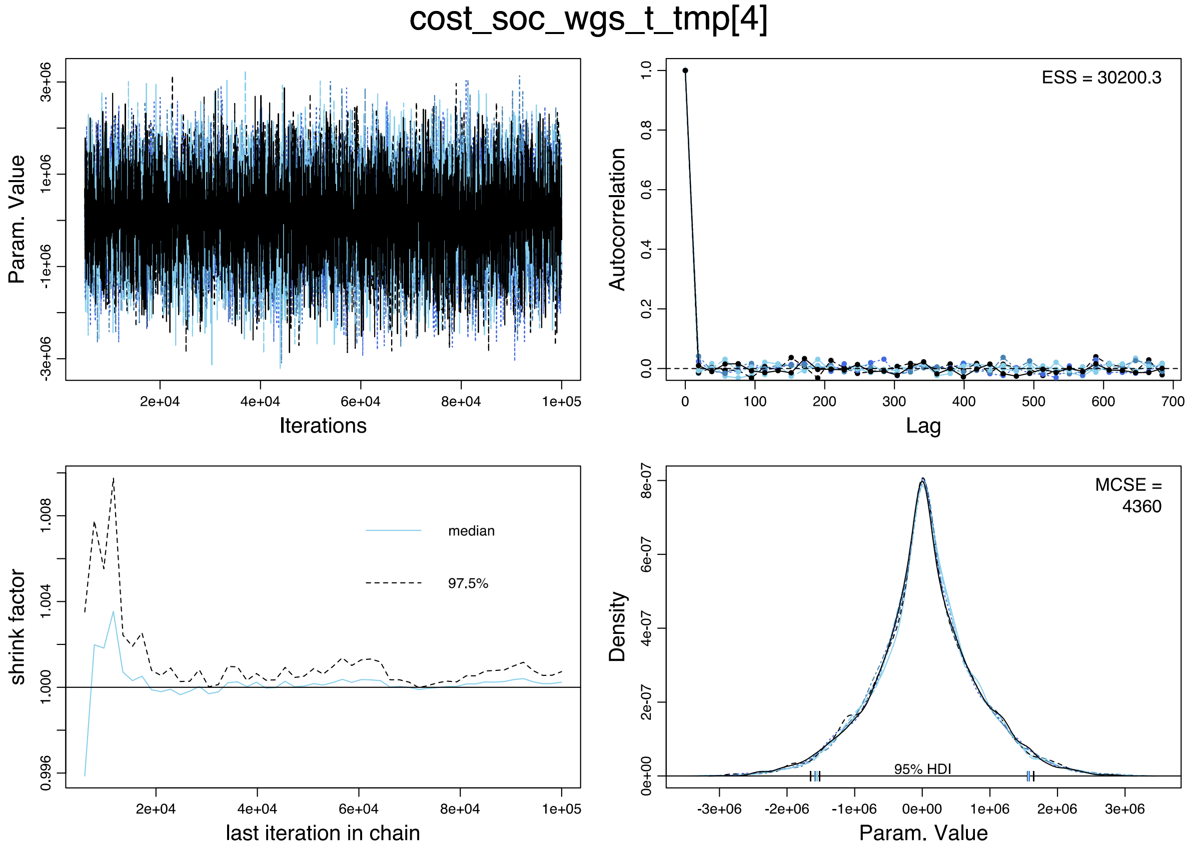

© 2024 Nurchis MC et al. *JAMA Network Open*.

eFigure 26. Convergence Diagnostics for the Output of JAGS Related to the Cost Parameter of the Second-Line WGS Strategy in the Fifth Health State

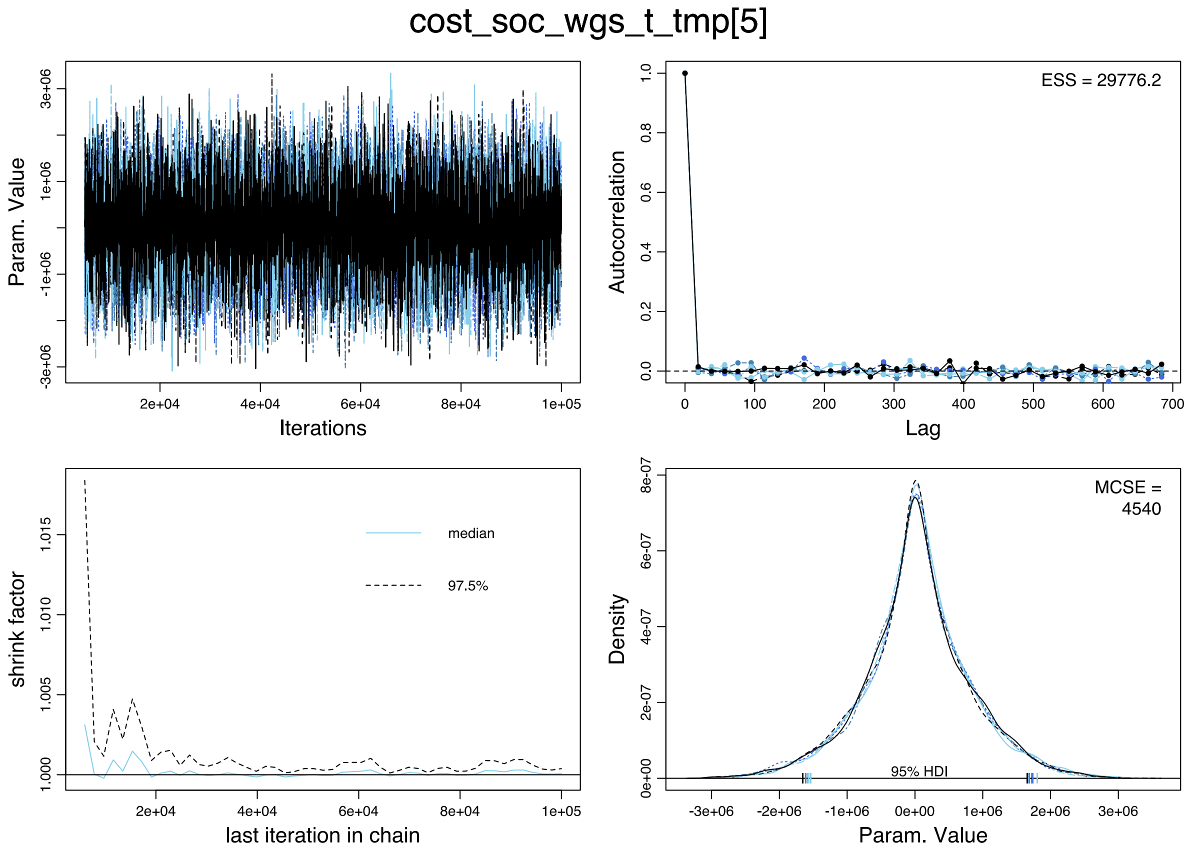

© 2024 Nurchis MC et al. *JAMA Network Open*.

eFigure 27. Convergence Diagnostics for the Output of JAGS Related to the Cost Parameter of the Second-Line WGS Strategy in the Eighth Health State

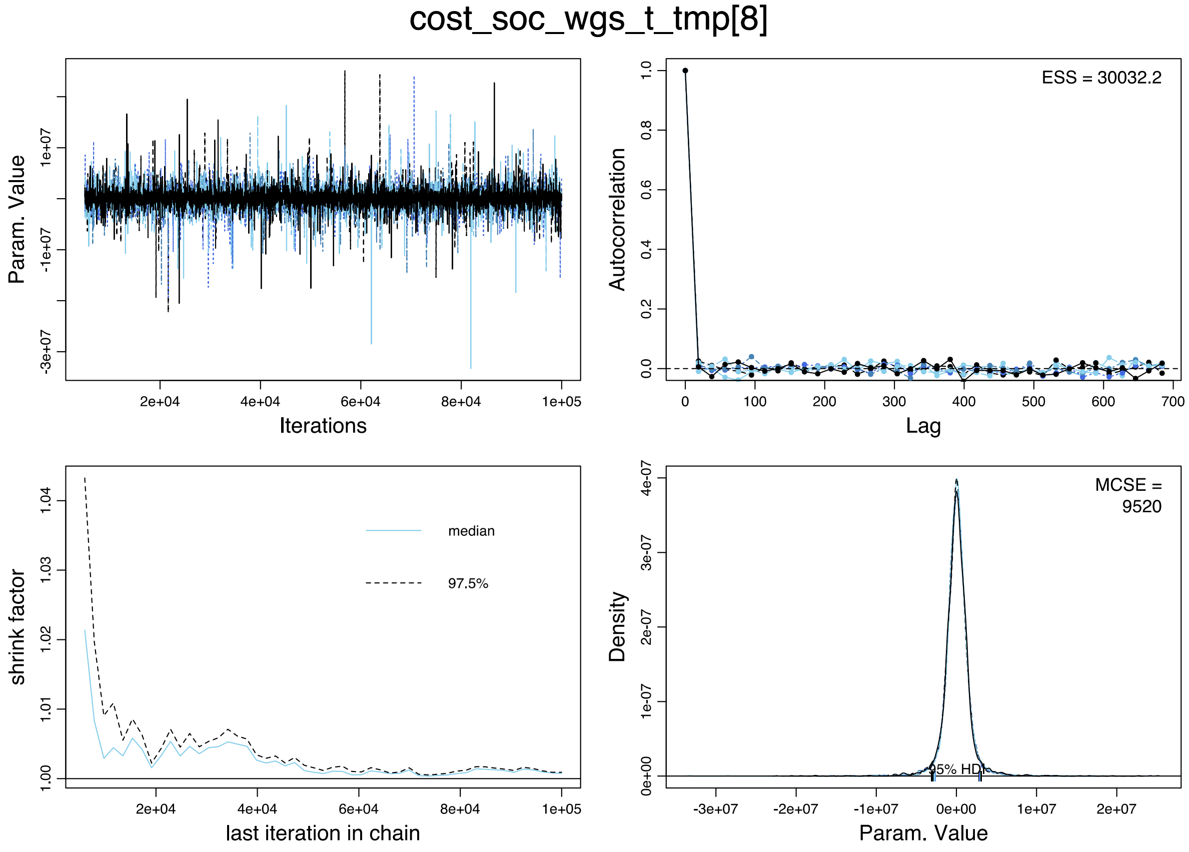

© 2024 Nurchis MC et al. *JAMA Network Open*.

eFigure 28. Convergence Diagnostics for the Output of JAGS Related to the Cost Parameter of the WES Strategy in the First Health State

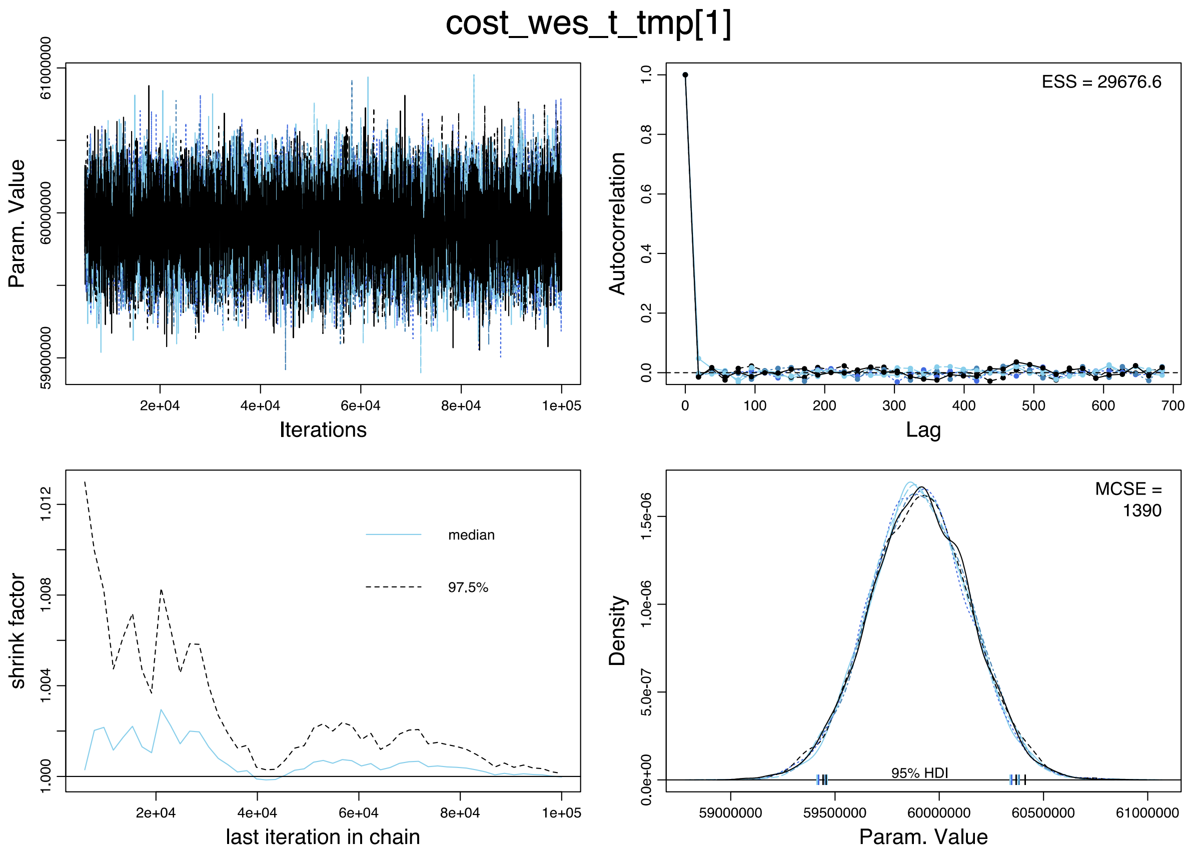

© 2024 Nurchis MC et al. *JAMA Network Open*.

eFigure 29. Convergence Diagnostics for the Output of JAGS Related to the Cost Parameter of the WES Strategy in the Second Health State

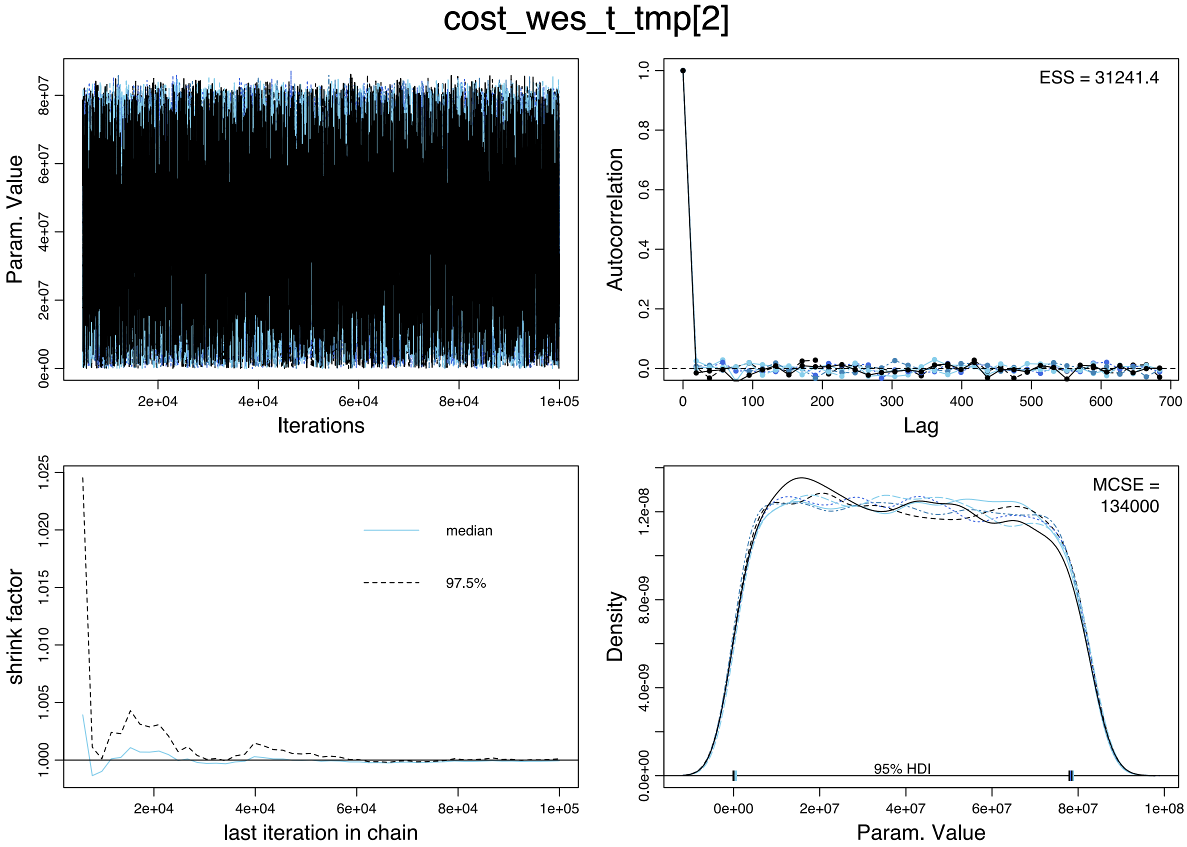

© 2024 Nurchis MC et al. *JAMA Network Open*.

eFigure 30. Convergence Diagnostics for the Output of JAGS Related to the Cost Parameter of the WES Strategy in the Third Health State

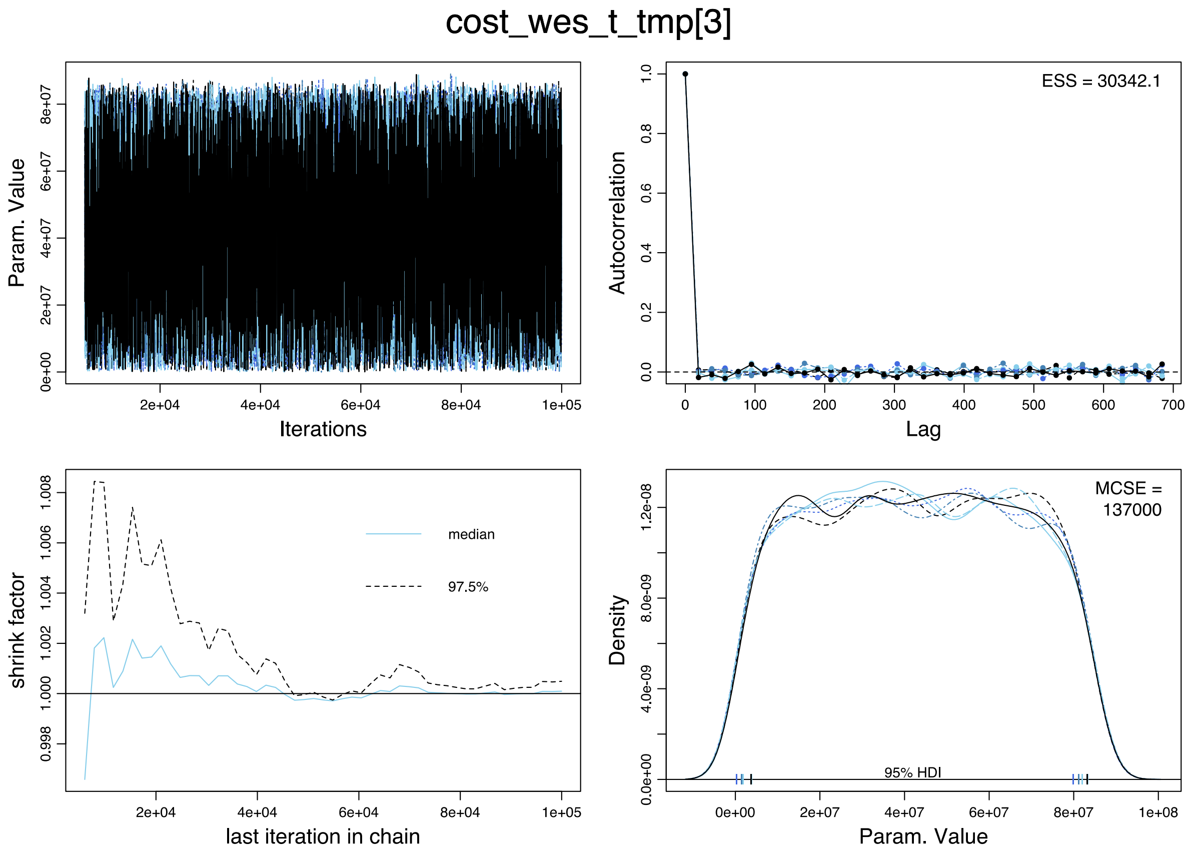

© 2024 Nurchis MC et al. *JAMA Network Open*.

eFigure 31. Convergence Diagnostics for the Output of JAGS Related to the Cost Parameter of the WES Strategy in the Eighth Health State

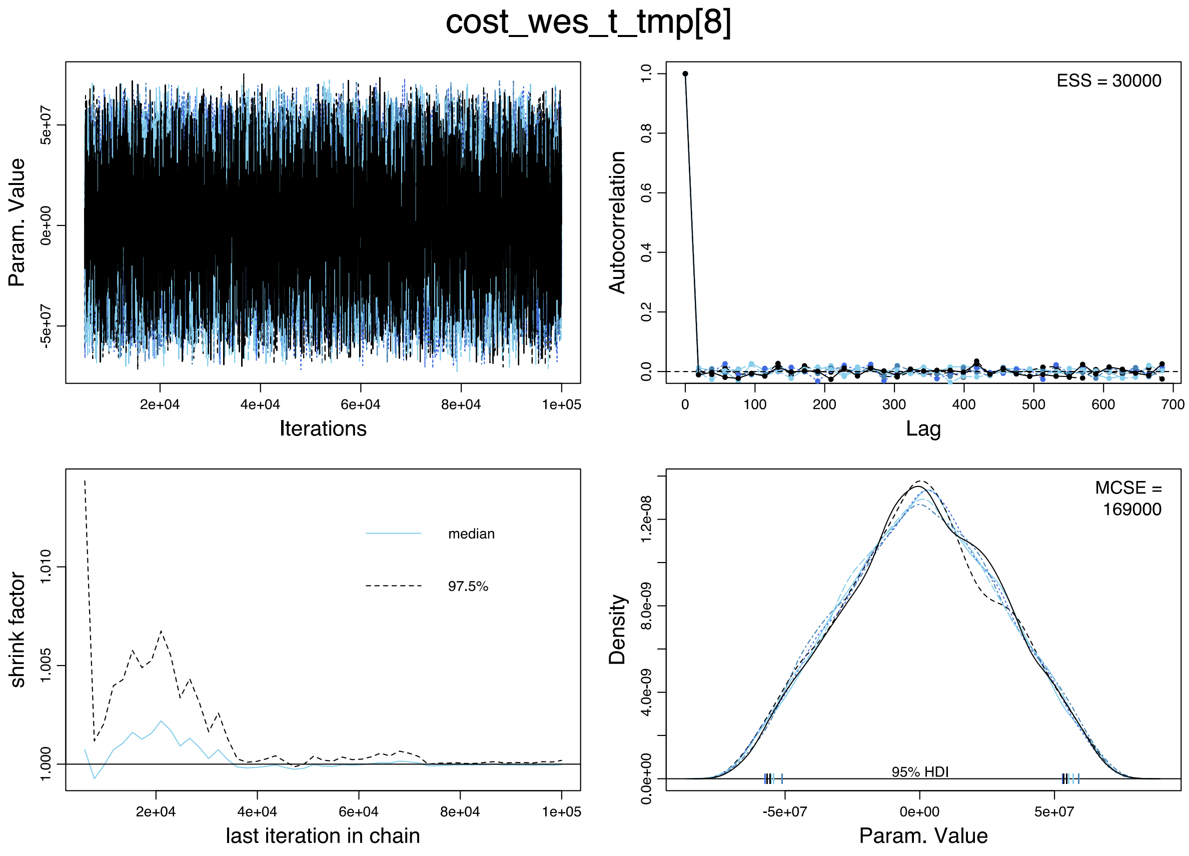

© 2024 Nurchis MC et al. *JAMA Network Open*.

eFigure 32. Convergence Diagnostics for the Output of JAGS Related to the Cost Parameter of the WGS Strategy in the First Health State

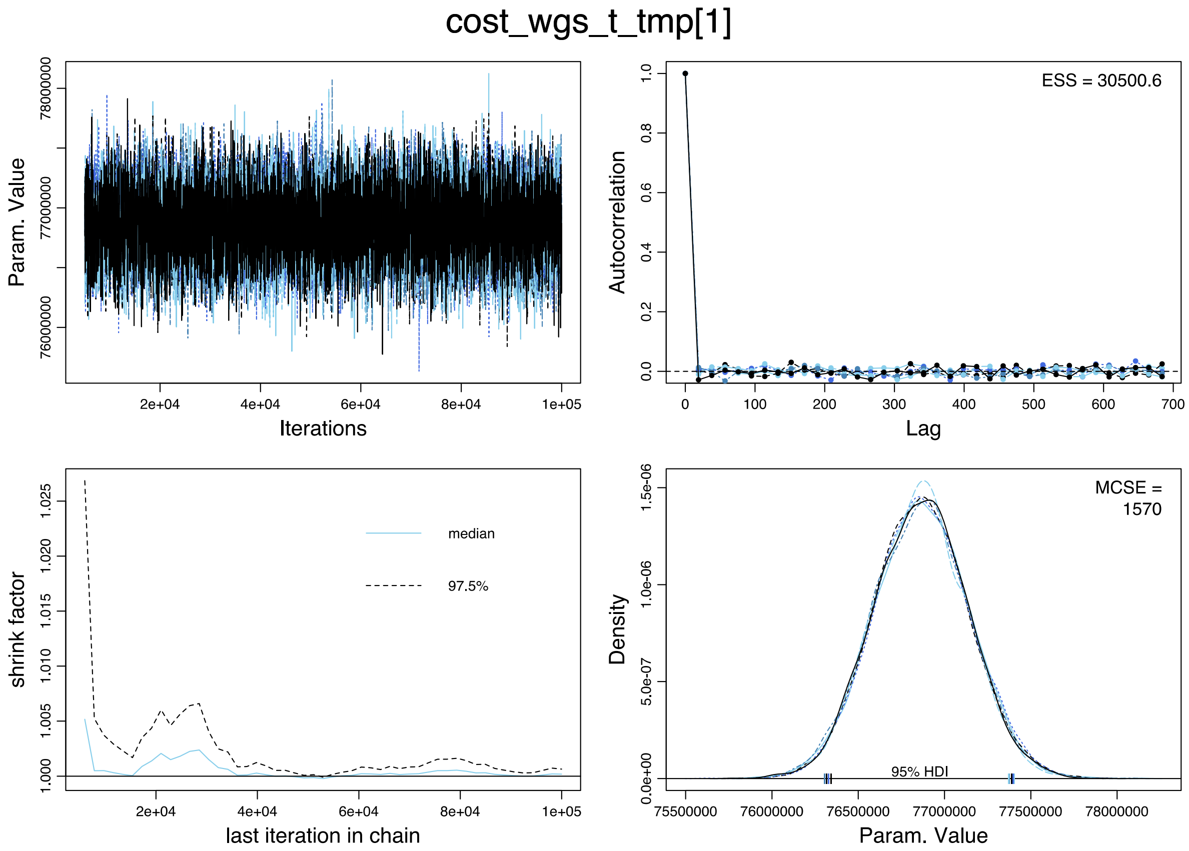

© 2024 Nurchis MC et al. *JAMA Network Open*.

eFigure 33. Convergence Diagnostics for the Output of JAGS Related to the Cost Parameter of the WGS Strategy in the Second Health State

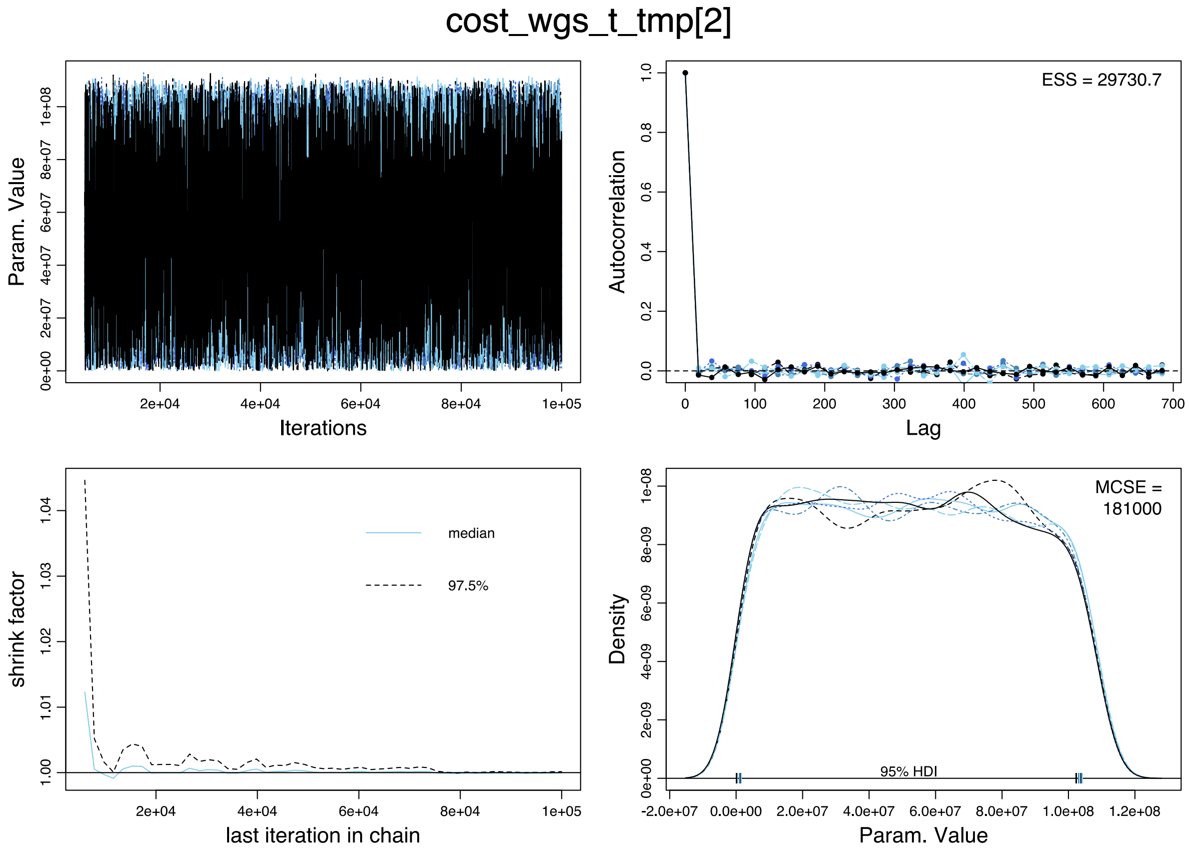

© 2024 Nurchis MC et al. *JAMA Network Open*.

eFigure 34. Convergence Diagnostics for the Output of JAGS Related to the Cost Parameter of the WGS Strategy in the Third Health State

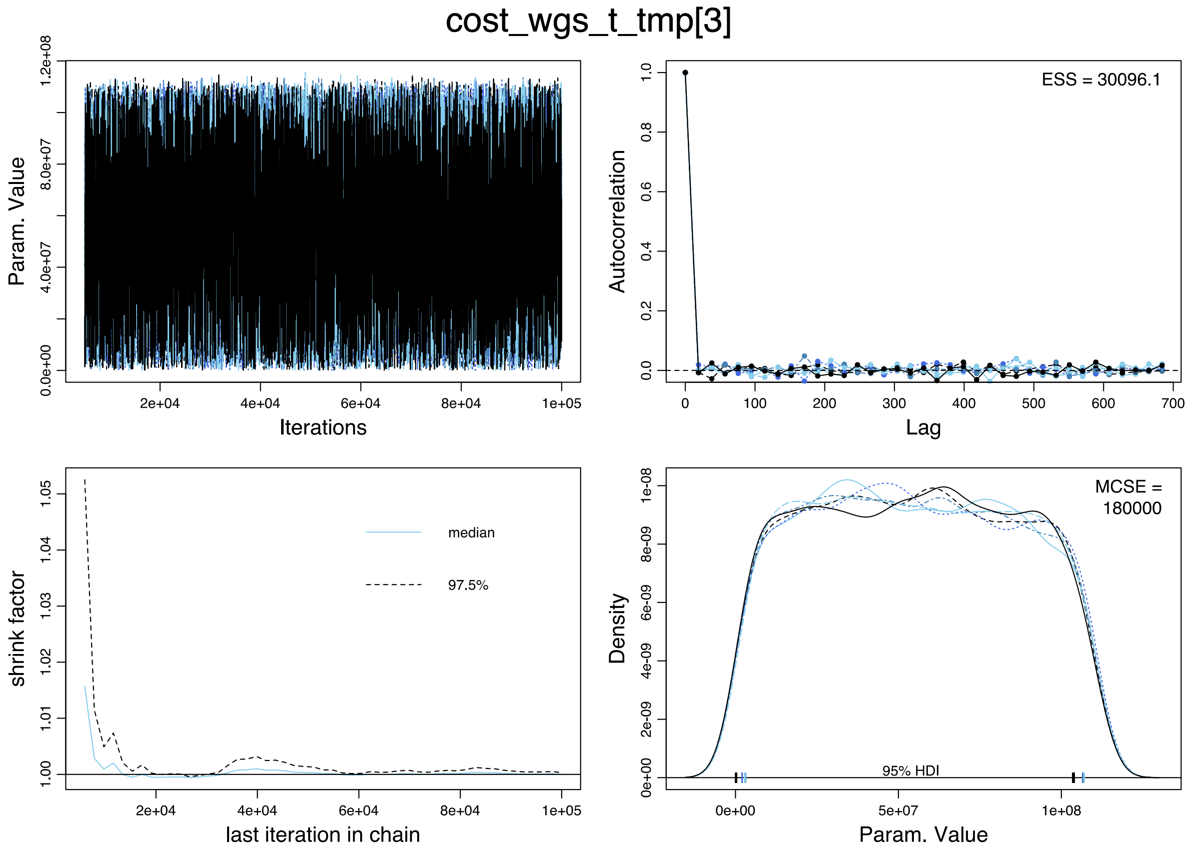

© 2024 Nurchis MC et al. *JAMA Network Open*.

eFigure 35. Convergence Diagnostics for the Output of JAGS Related to the Cost Parameter of the WGS Strategy in the Eighth Health State

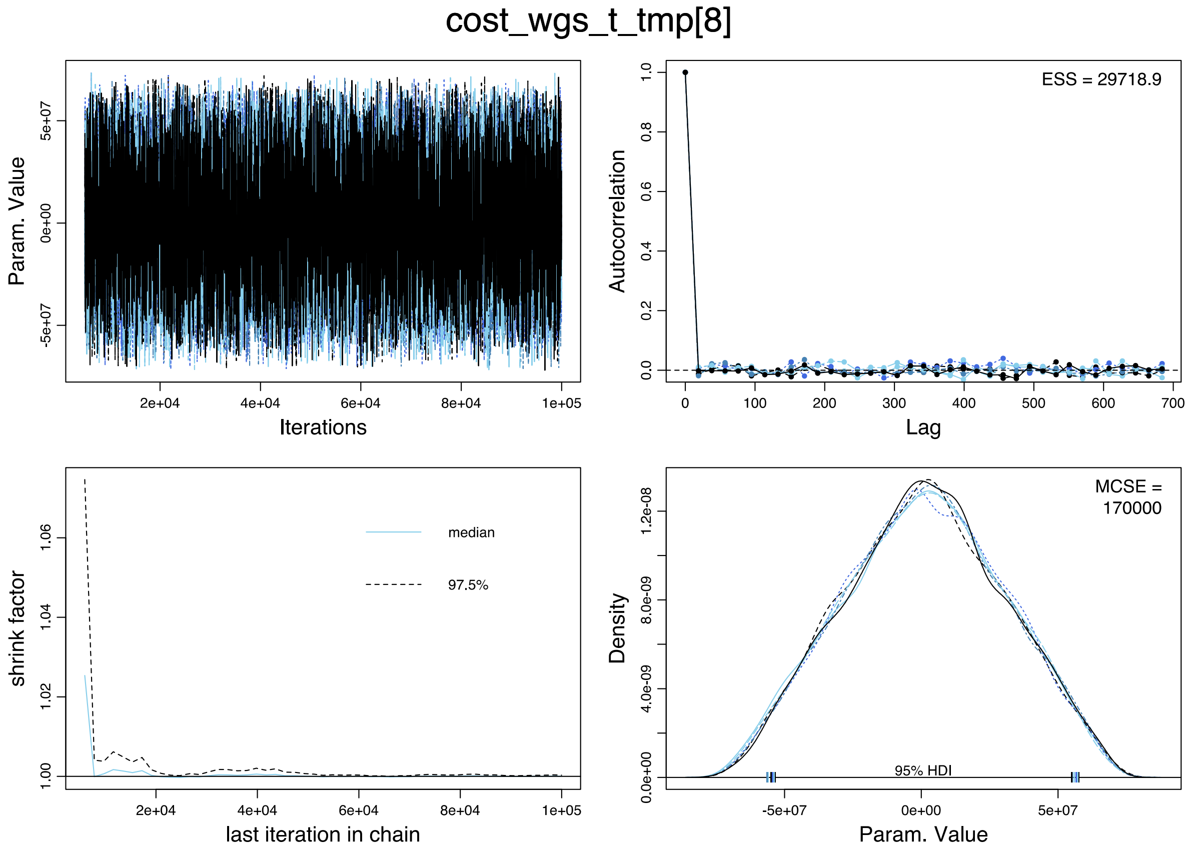

© 2024 Nurchis MC et al. *JAMA Network Open*.

eFigure 36. Convergence Diagnostics for the Output of JAGS Related to the Cost Parameter of the SOC Strategy in the First Health State (Prior)

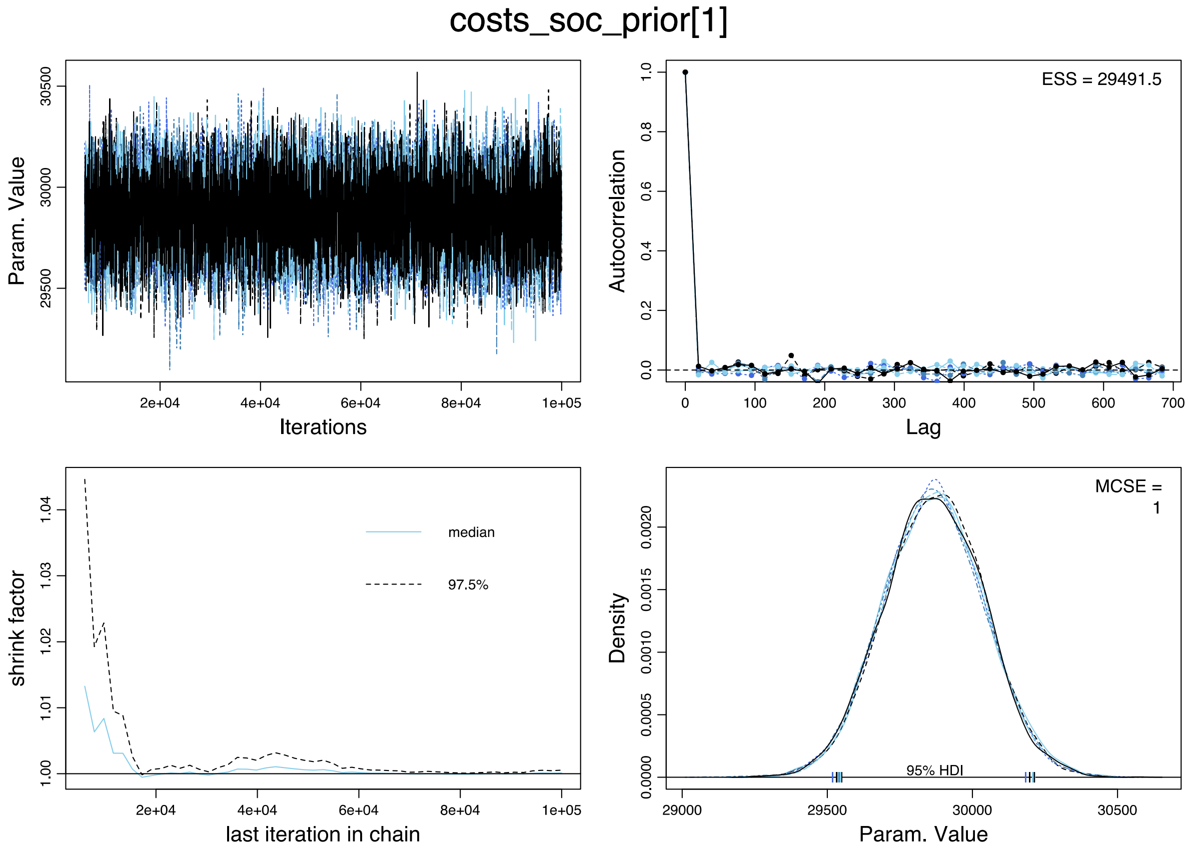

© 2024 Nurchis MC et al. *JAMA Network Open*.

eFigure 37. Convergence Diagnostics for the Output of JAGS Related to the Cost Parameter of the SOC Strategy in the Second Health State (Prior)

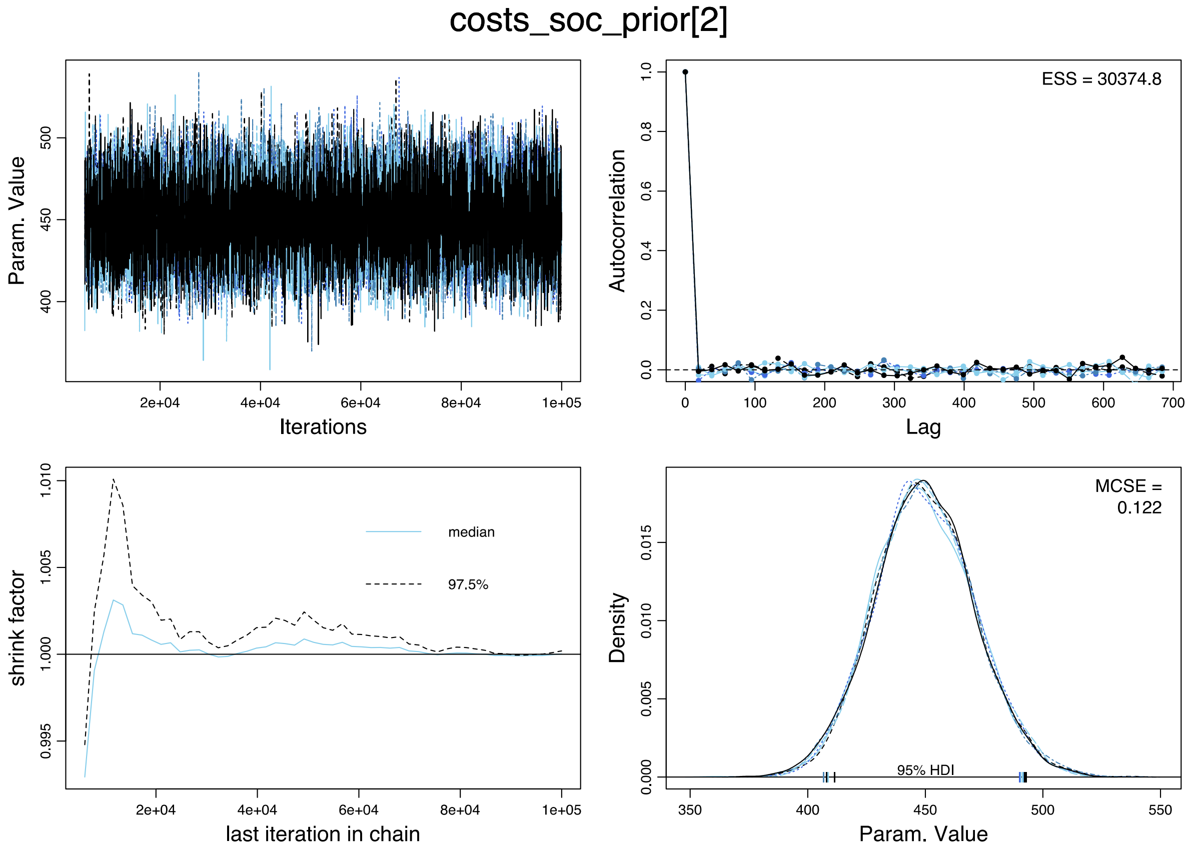

© 2024 Nurchis MC et al. *JAMA Network Open*.

eFigure 38. Convergence Diagnostics for the Output of JAGS Related to the Cost Parameter of the SOC Strategy in the Third Health State (Prior)

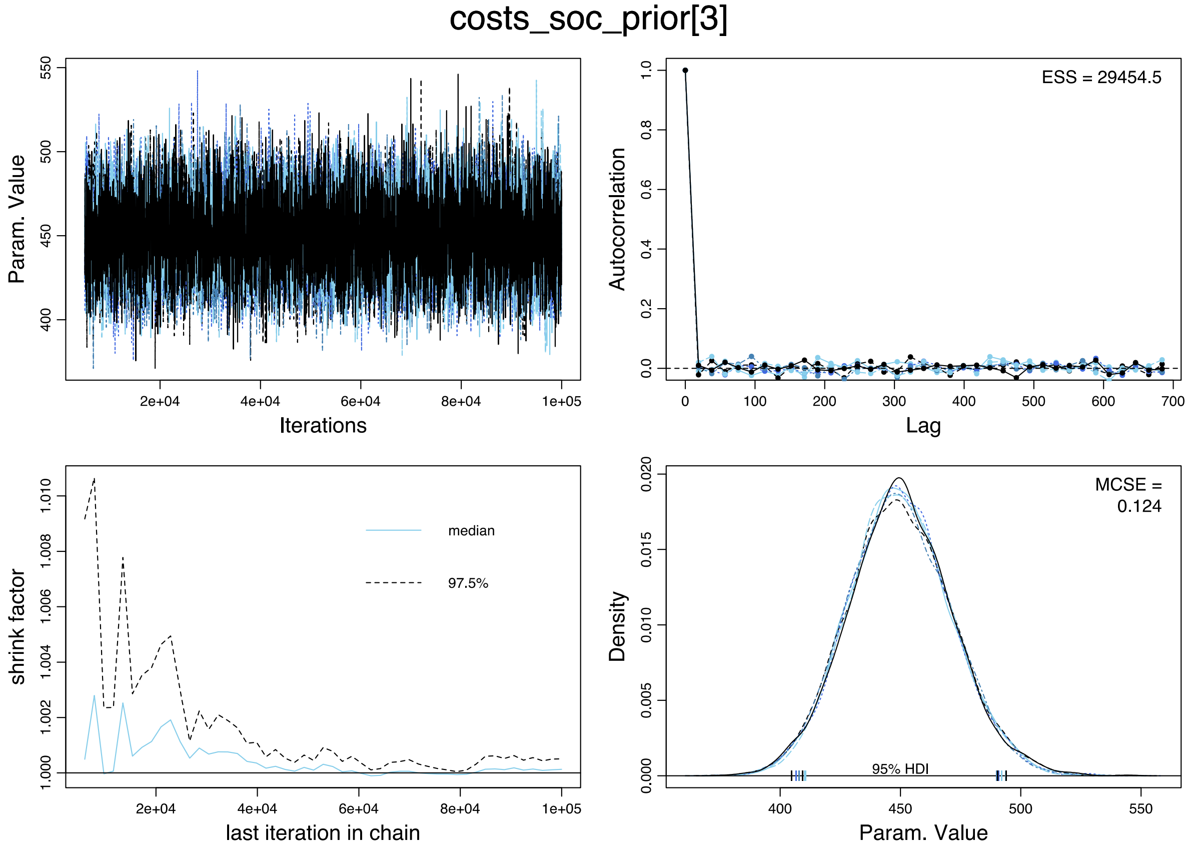

© 2024 Nurchis MC et al. *JAMA Network Open*.

eFigure 39. Convergence Diagnostics for the Output of JAGS Related to the Cost Parameter of the SOC Strategy in the Eighth Health State (Prior)

© 2024 Nurchis MC et al. *JAMA Network Open*.

costs_soc_prior[8]

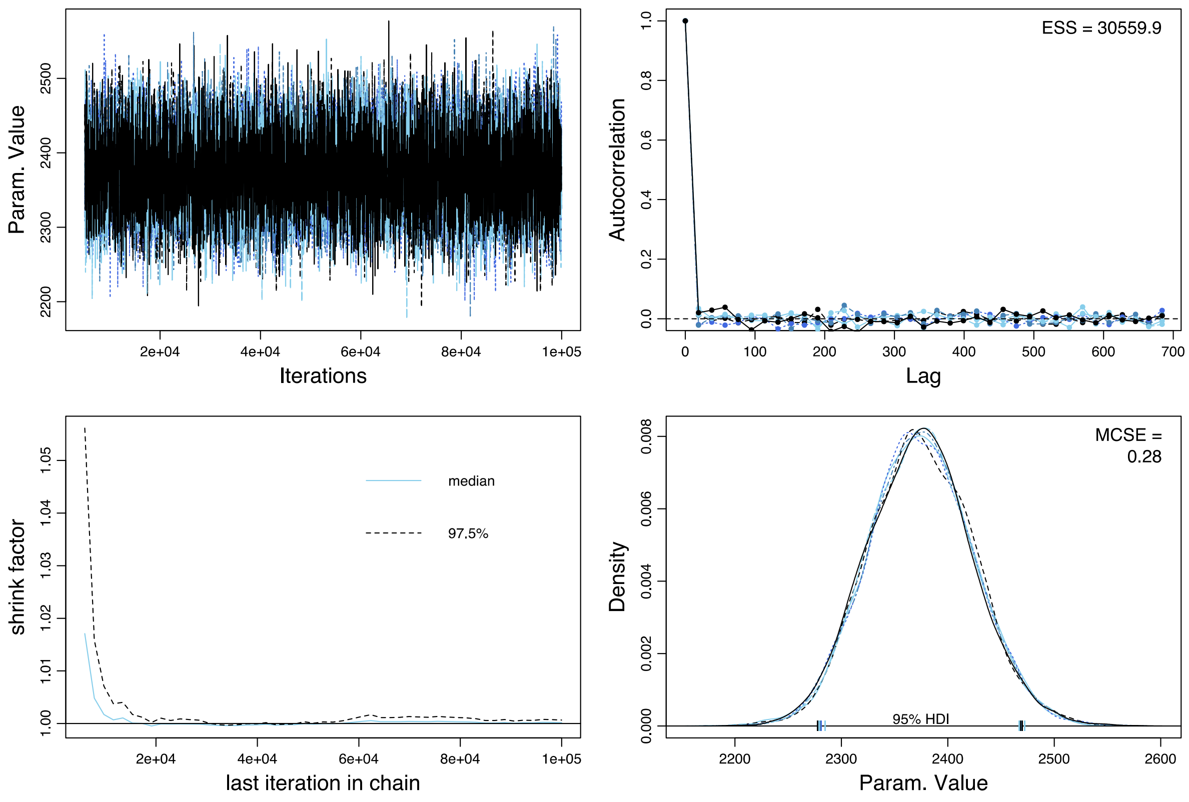

© 2024 Nurchis MC et al. *JAMA Network Open*.

eFigure 40. Convergence Diagnostics for the Output of JAGS Related to the Cost Parameter of the Second-Line WES Strategy in the First Health State (Prior)

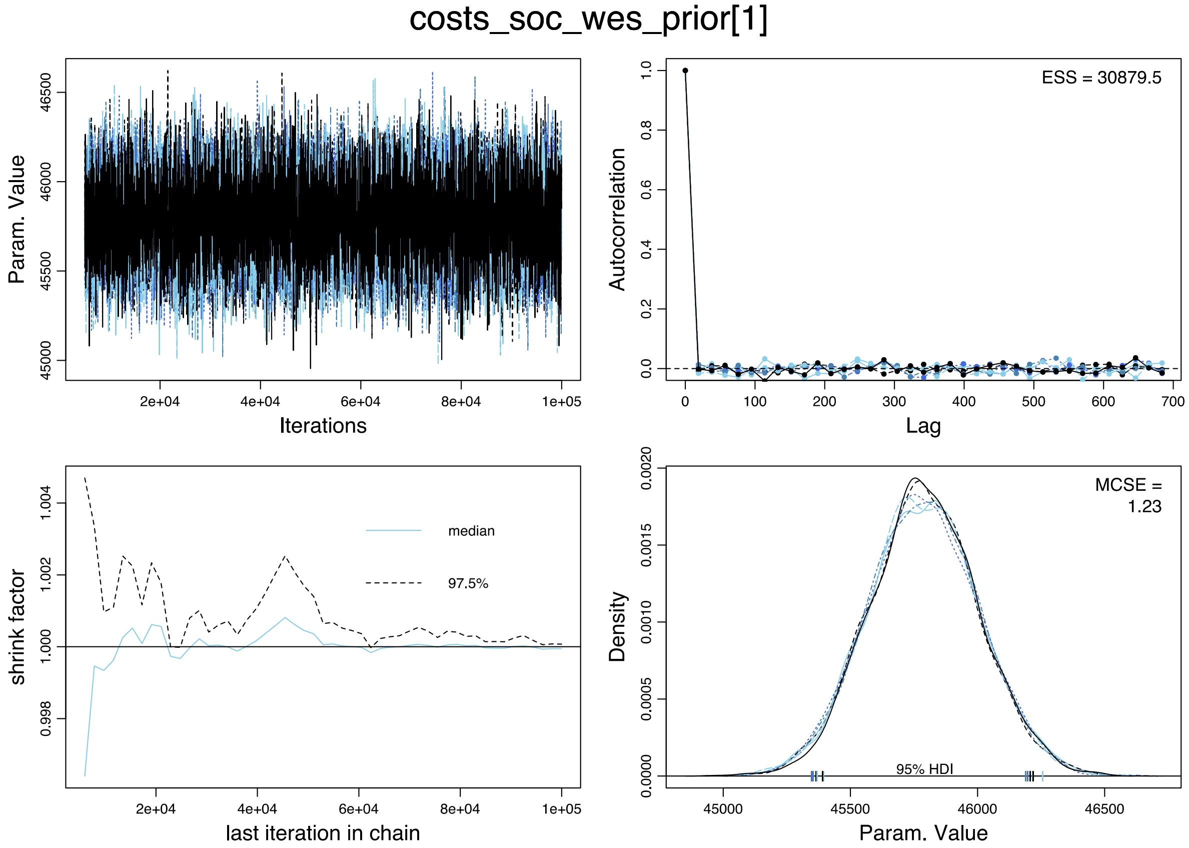

© 2024 Nurchis MC et al. *JAMA Network Open*.

eFigure 41. Convergence Diagnostics for the Output of JAGS Related to the Cost Parameter of the Second-Line WES Strategy in the Second Health State (Prior)

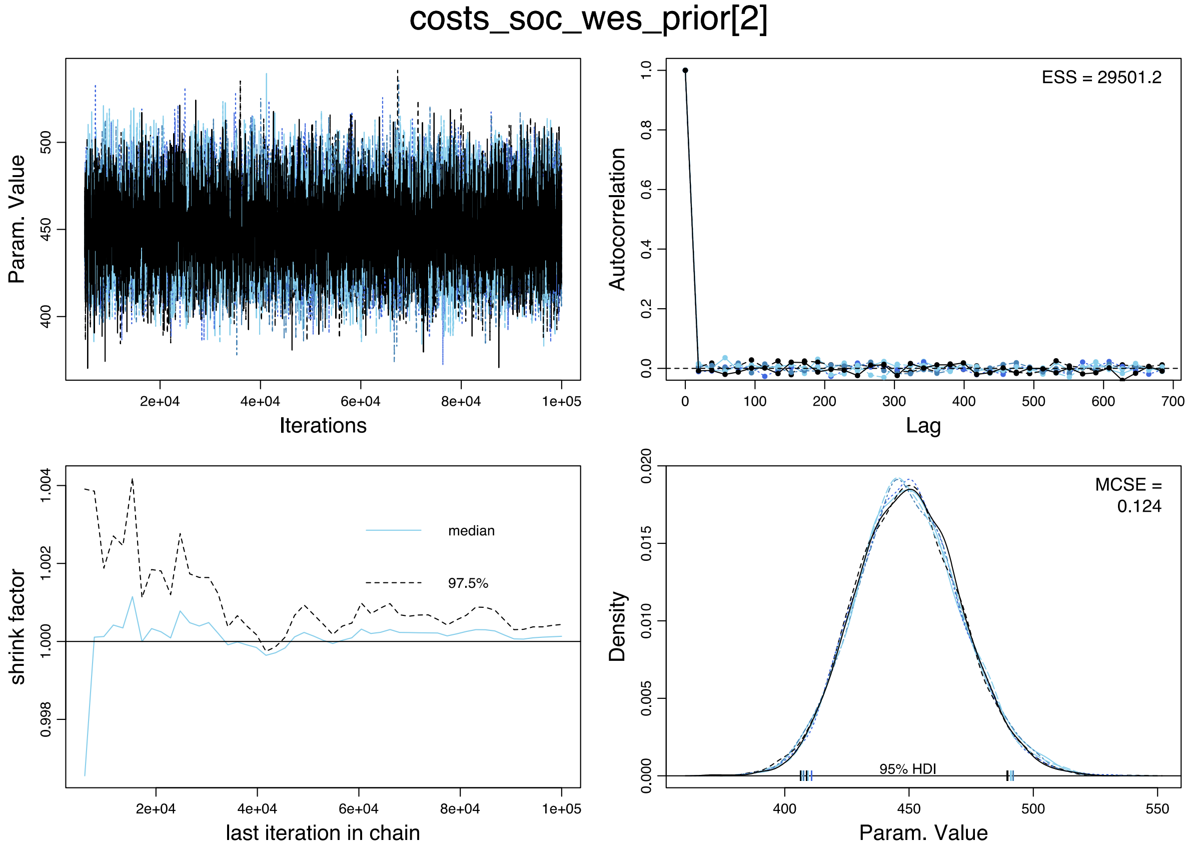

© 2024 Nurchis MC et al. *JAMA Network Open*.

eFigure 42. Convergence Diagnostics for the Output of JAGS Related to the Cost Parameter of the Second-Line WES Strategy in the Third Health State (Prior)

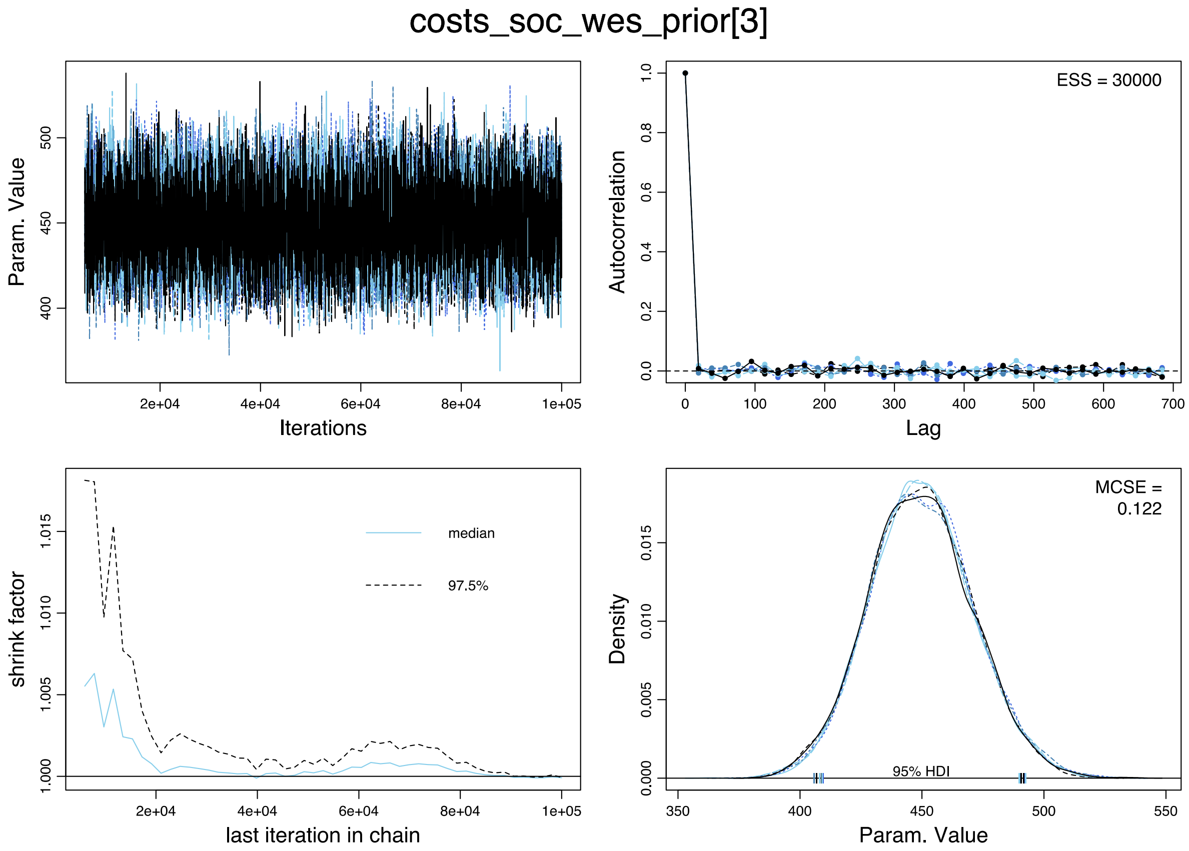

© 2024 Nurchis MC et al. *JAMA Network Open*.

eFigure 43. Convergence Diagnostics for the Output of JAGS Related to the Cost Parameter of the Second-Line WES Strategy in the Fourth Health State (Prior)

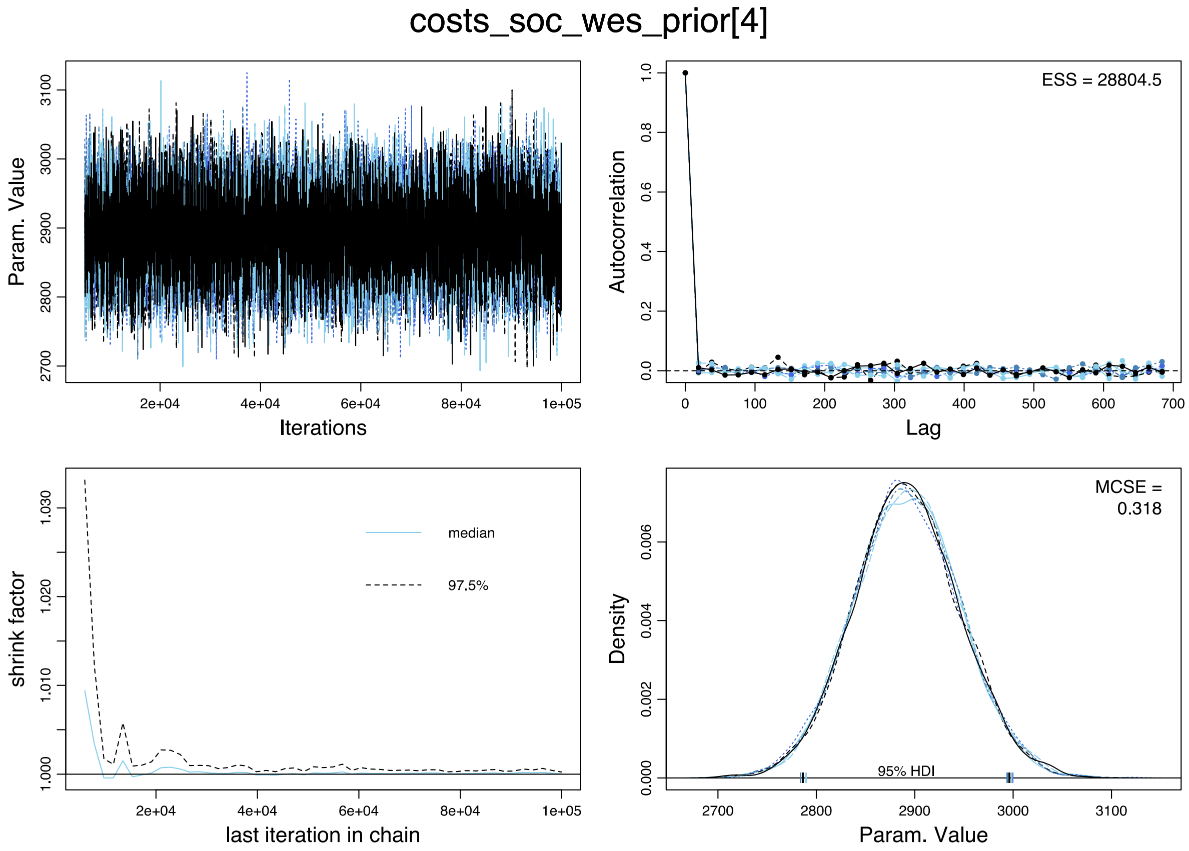

© 2024 Nurchis MC et al. *JAMA Network Open*.

eFigure 44. Convergence Diagnostics for the Output of JAGS Related to the Cost Parameter of the Second-Line WES Strategy in the Fifth Health State (Prior)

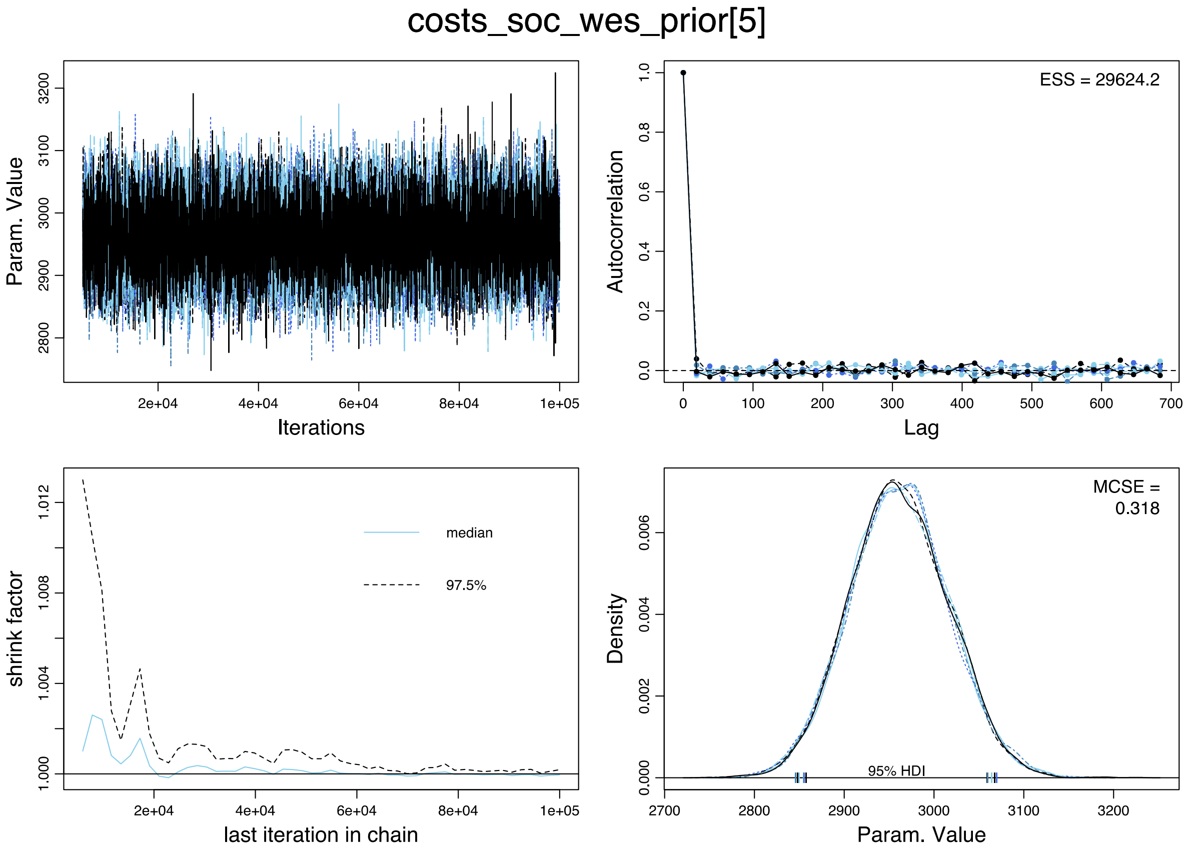

© 2024 Nurchis MC et al. *JAMA Network Open*.

eFigure 45. Convergence Diagnostics for the Output of JAGS Related to the Cost Parameter of the Second-Line WES Strategy in the Eighth Health State (Prior)

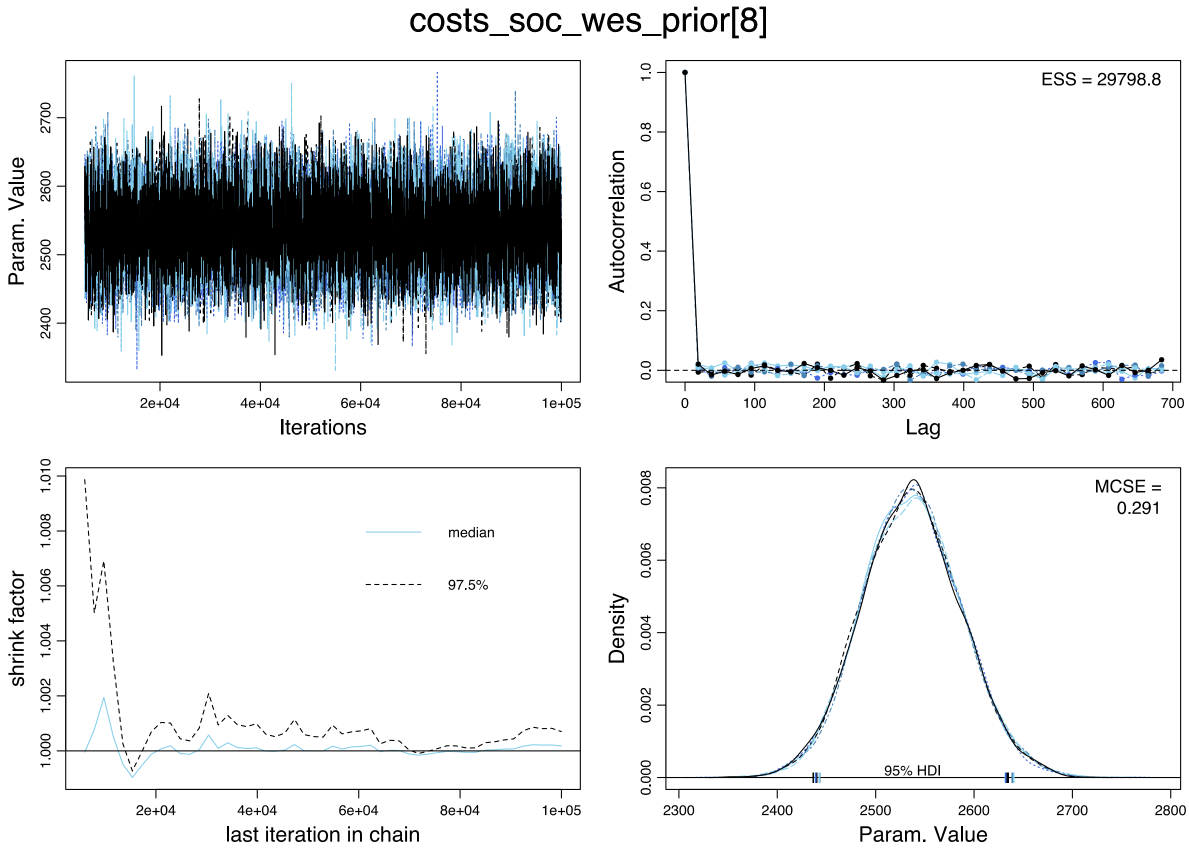

© 2024 Nurchis MC et al. *JAMA Network Open*.

eFigure 46. Convergence Diagnostics for the Output of JAGS Related to the Cost Parameter of the Second-Line WGS Strategy in the First Health State (Prior)

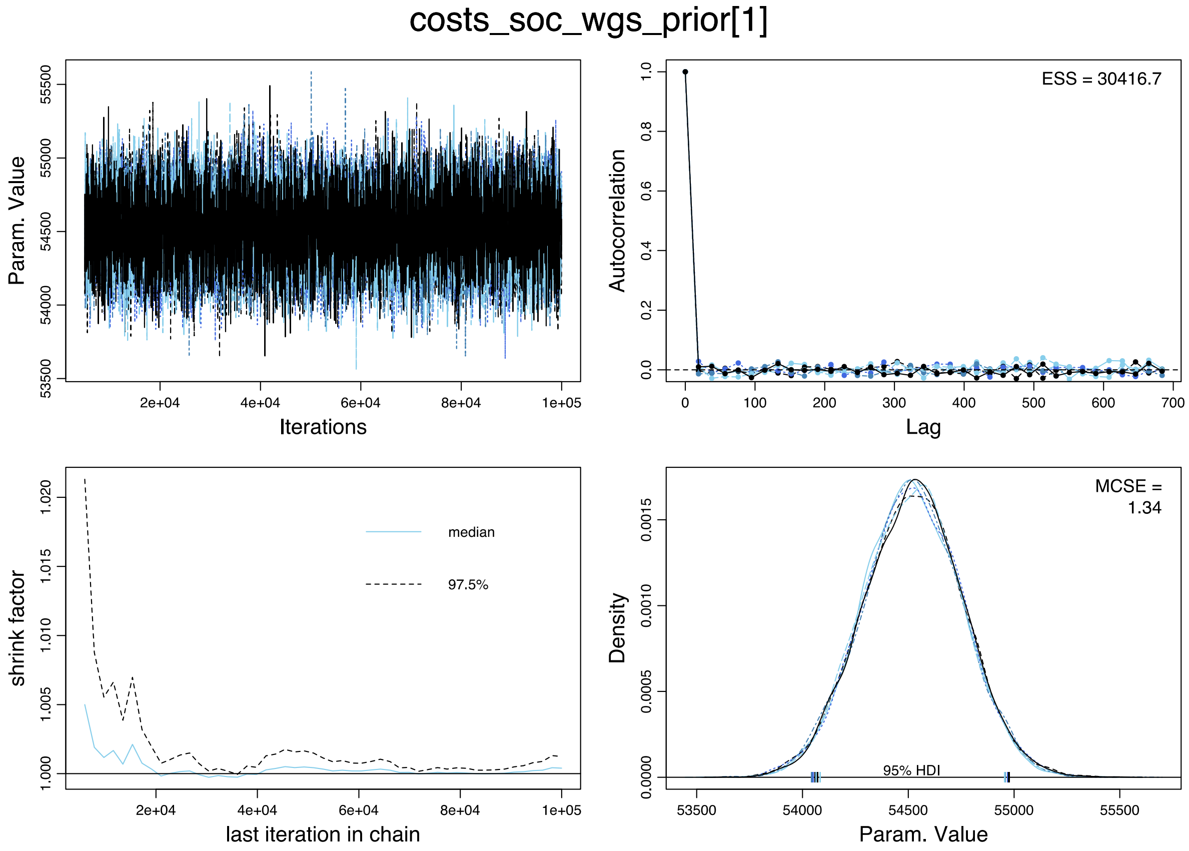

© 2024 Nurchis MC et al. *JAMA Network Open*.

eFigure 47. Convergence Diagnostics for the Output of JAGS Related to the Cost Parameter of the Second-Line WGS Strategy in the Second Health State (Prior)

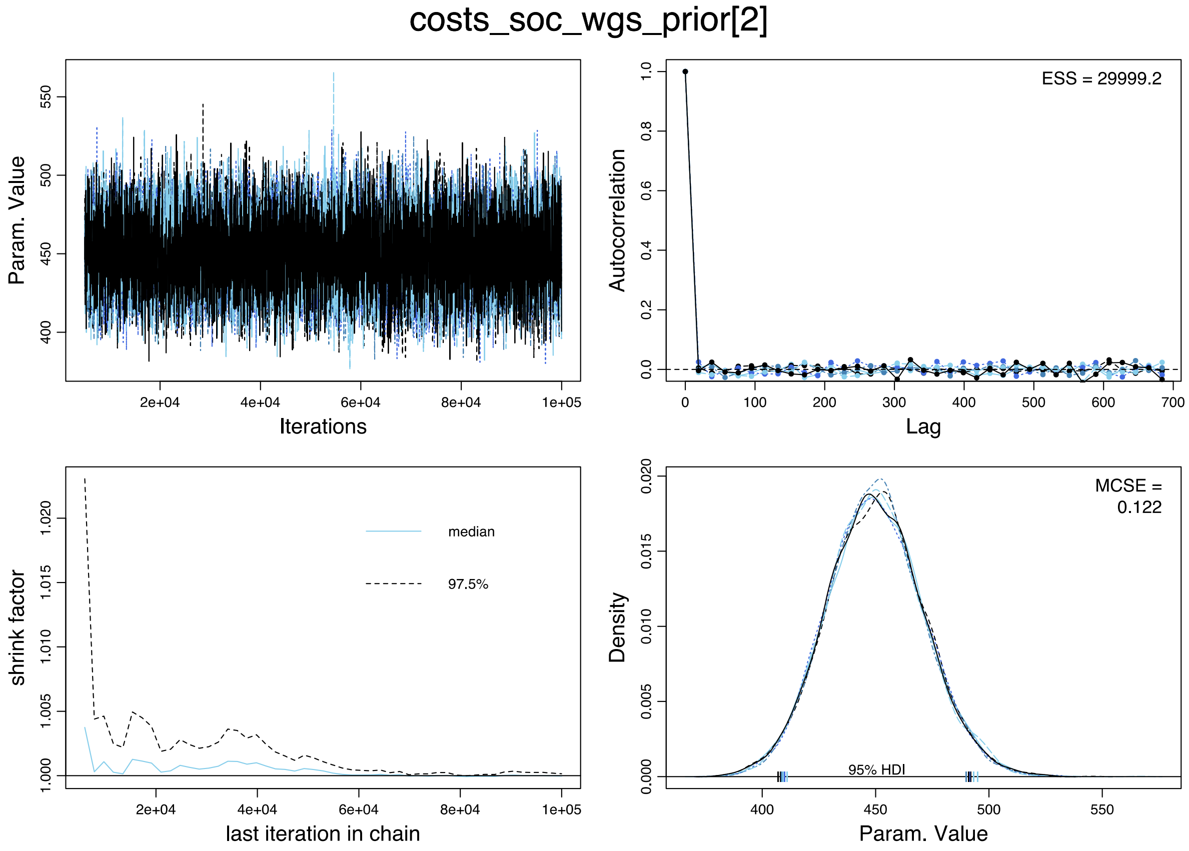

© 2024 Nurchis MC et al. *JAMA Network Open*.

eFigure 48. Convergence Diagnostics for the Output of JAGS Related to the Cost Parameter of the Second-Line WGS Strategy in the Third Health State (Prior)

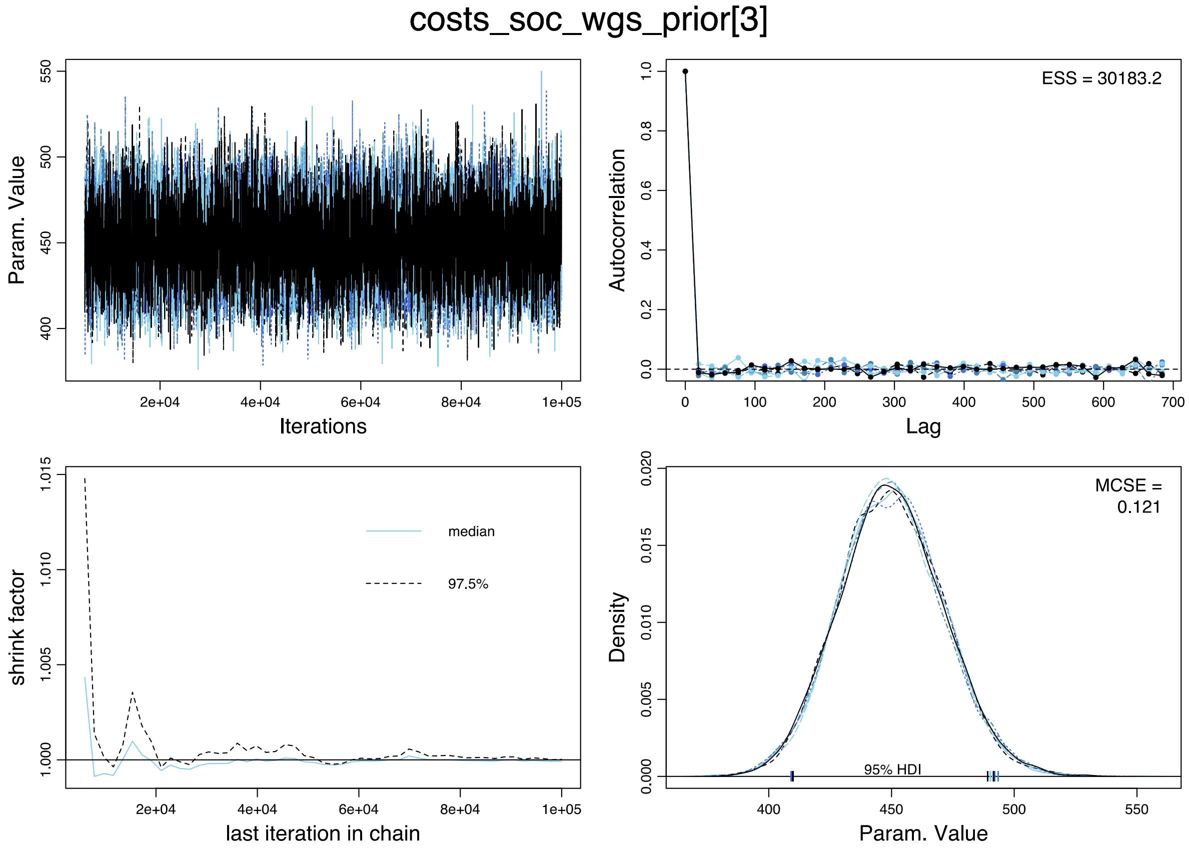

© 2024 Nurchis MC et al. *JAMA Network Open*.

eFigure 49. Convergence Diagnostics for the Output of JAGS Related to the Cost Parameter of the Second-Line WGS Strategy in the Fourth Health State (Prior)

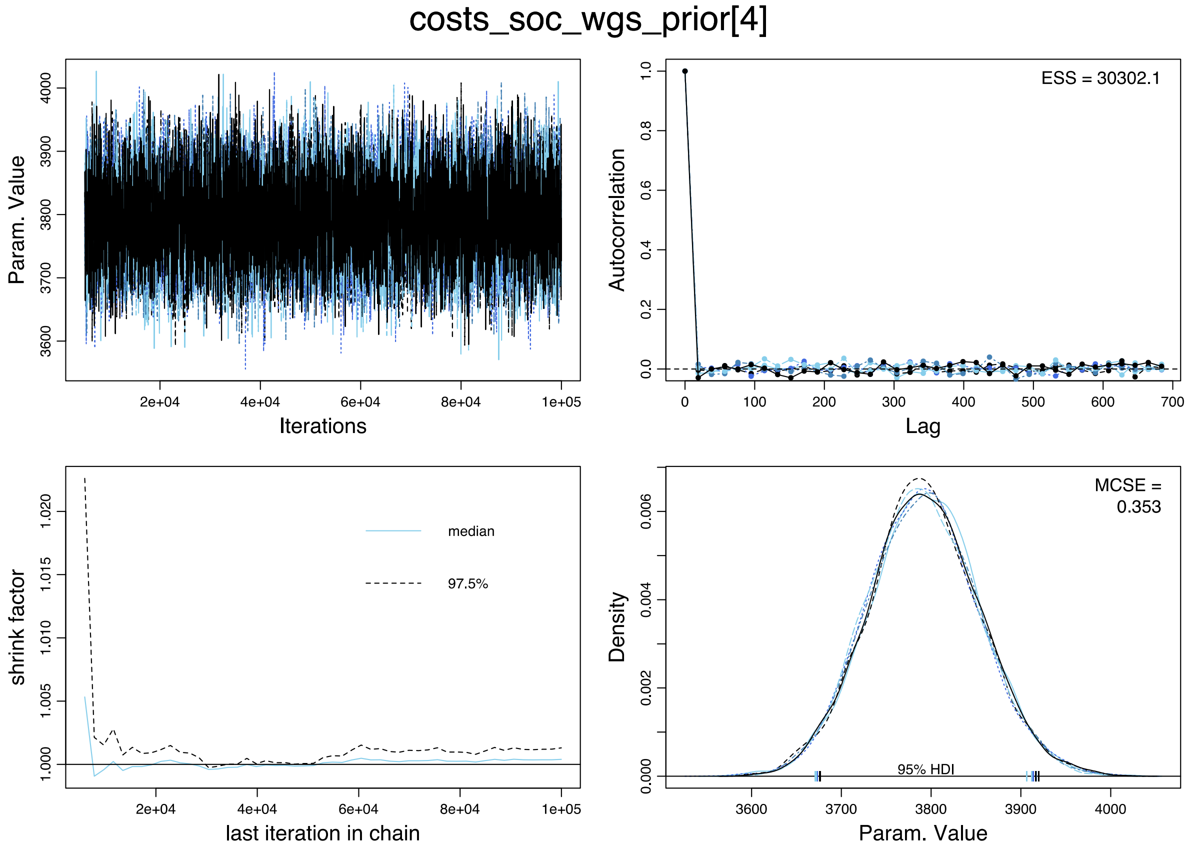

© 2024 Nurchis MC et al. *JAMA Network Open*.

eFigure 50. Convergence Diagnostics for the Output of JAGS Related to the Cost Parameter of the Second-Line WGS Strategy in the Fifth Health State (Prior)

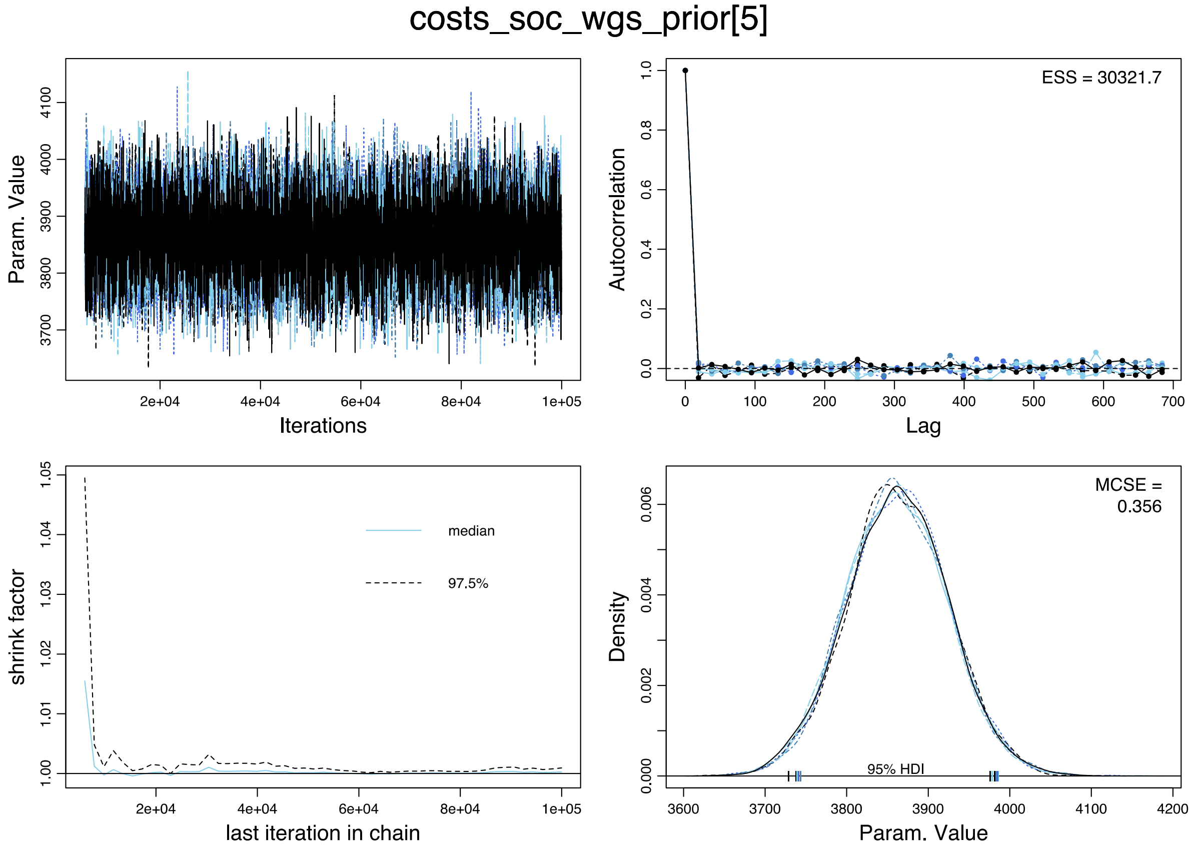

© 2024 Nurchis MC et al. *JAMA Network Open*.

eFigure 51. Convergence Diagnostics for the Output of JAGS Related to the Cost Parameter of the Second-Line WGS Strategy in the Eighth Health State (Prior)

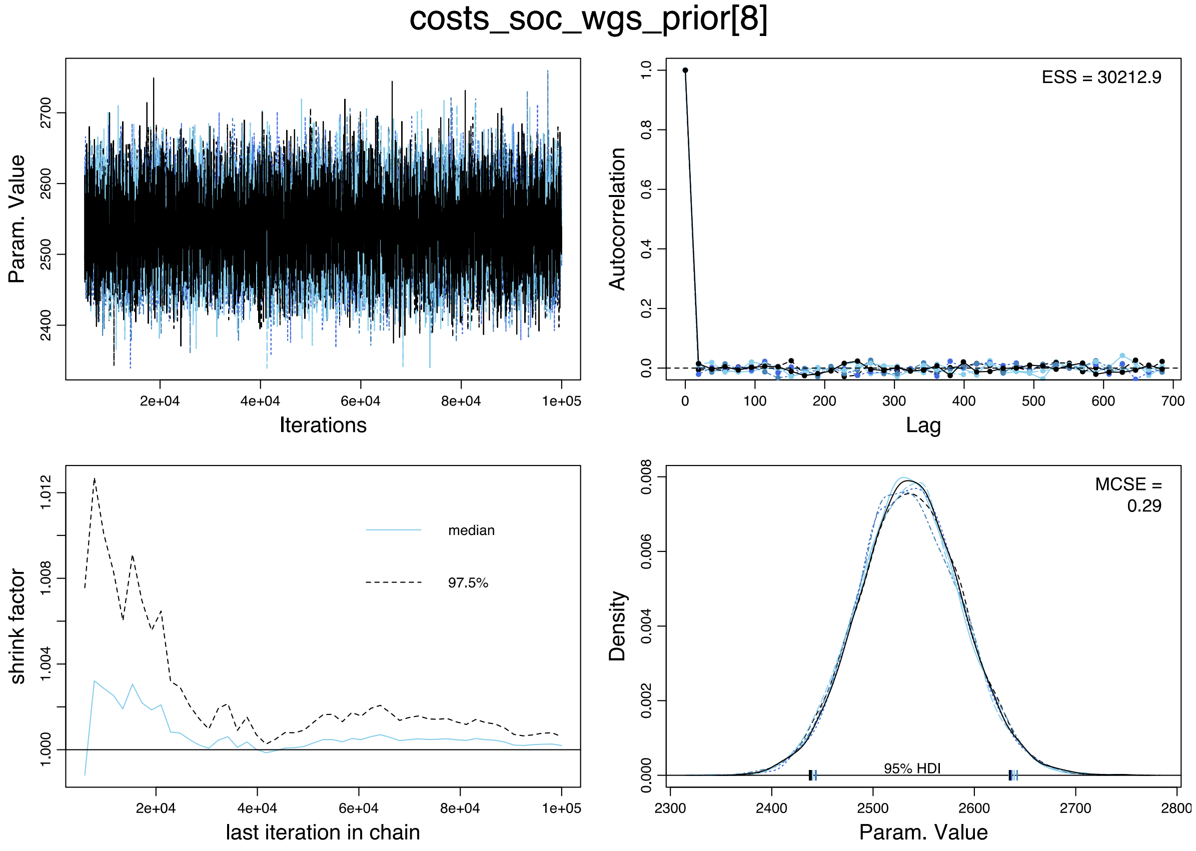

© 2024 Nurchis MC et al. *JAMA Network Open*.

eFigure 52. Convergence Diagnostics for the Output of JAGS Related to the Cost Parameter of the WES Strategy in the First Health State (Prior)

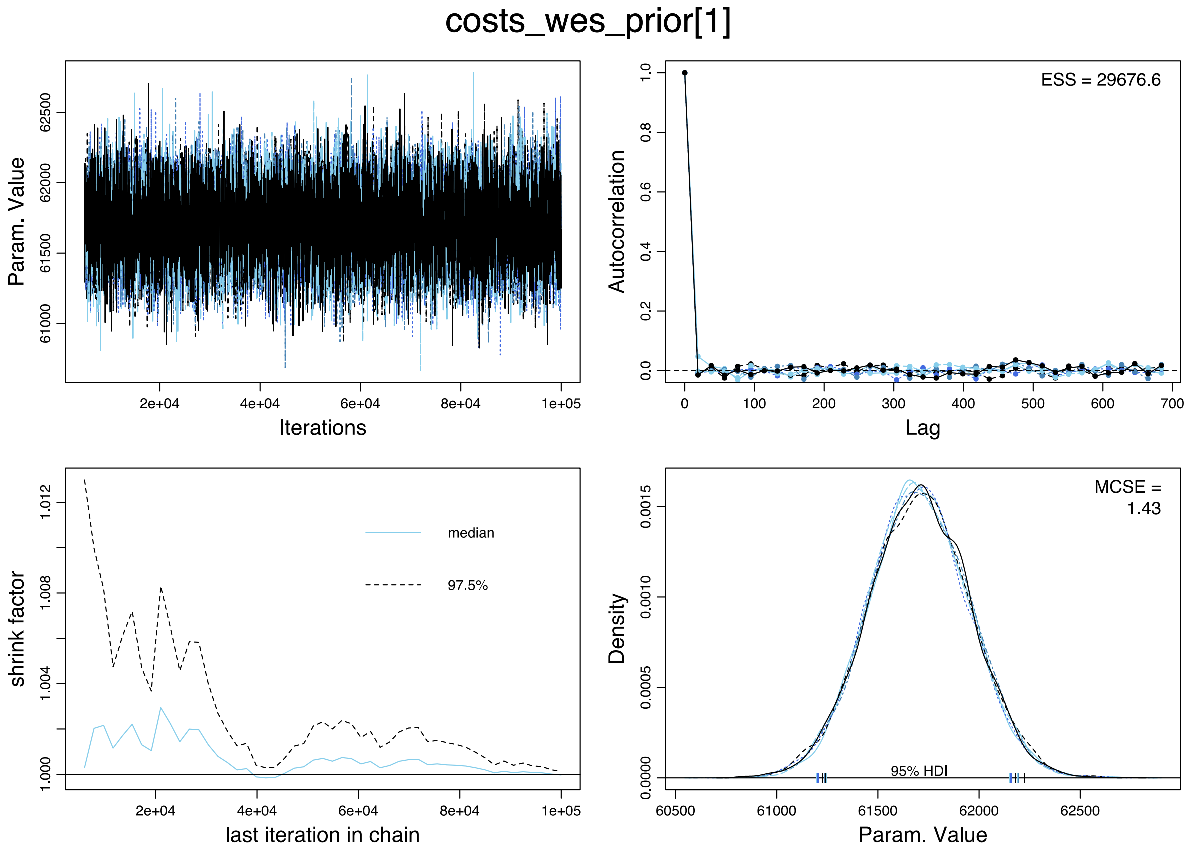

© 2024 Nurchis MC et al. *JAMA Network Open*.

eFigure 53. Convergence Diagnostics for the Output of JAGS Related to the Cost Parameter of the WES Strategy in the Second Health State (Prior)

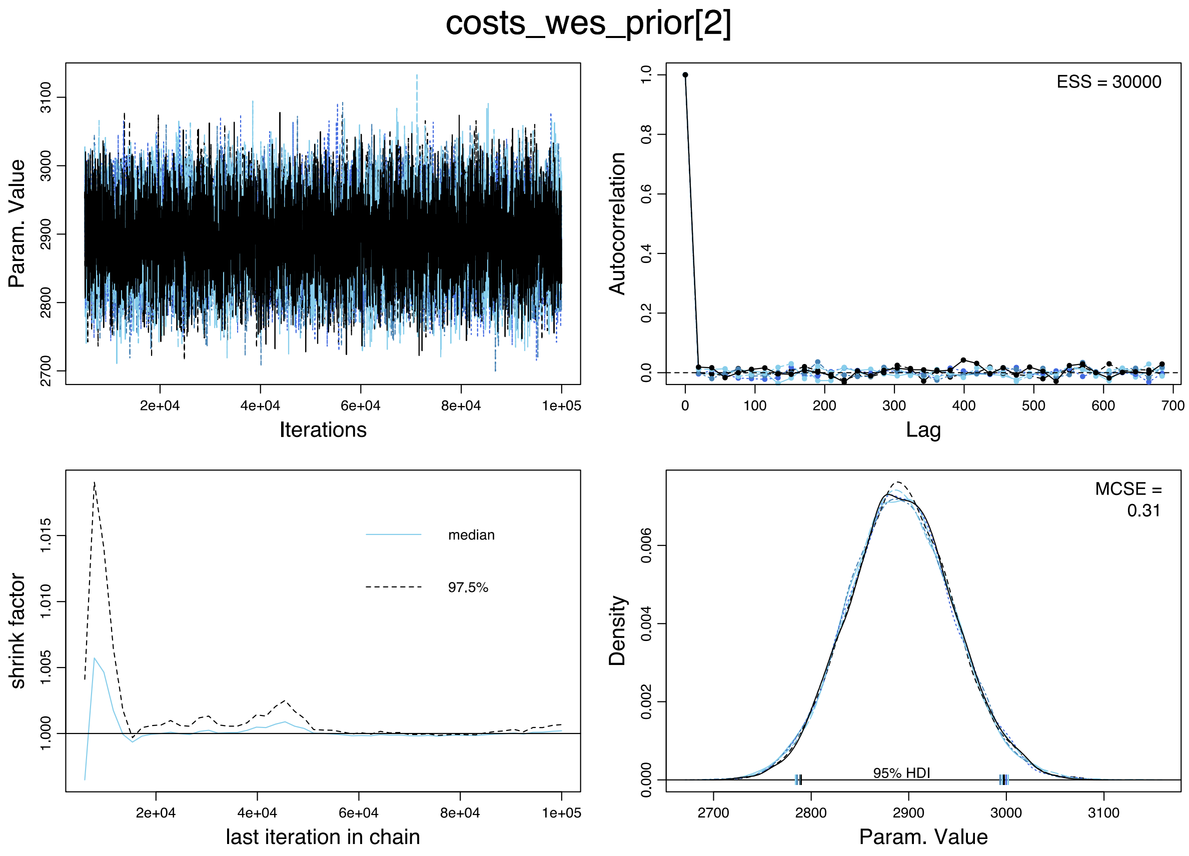

© 2024 Nurchis MC et al. *JAMA Network Open*.

eFigure 54. Convergence Diagnostics for the Output of JAGS Related to the Cost Parameter of the WES Strategy in the Third Health State (Prior)

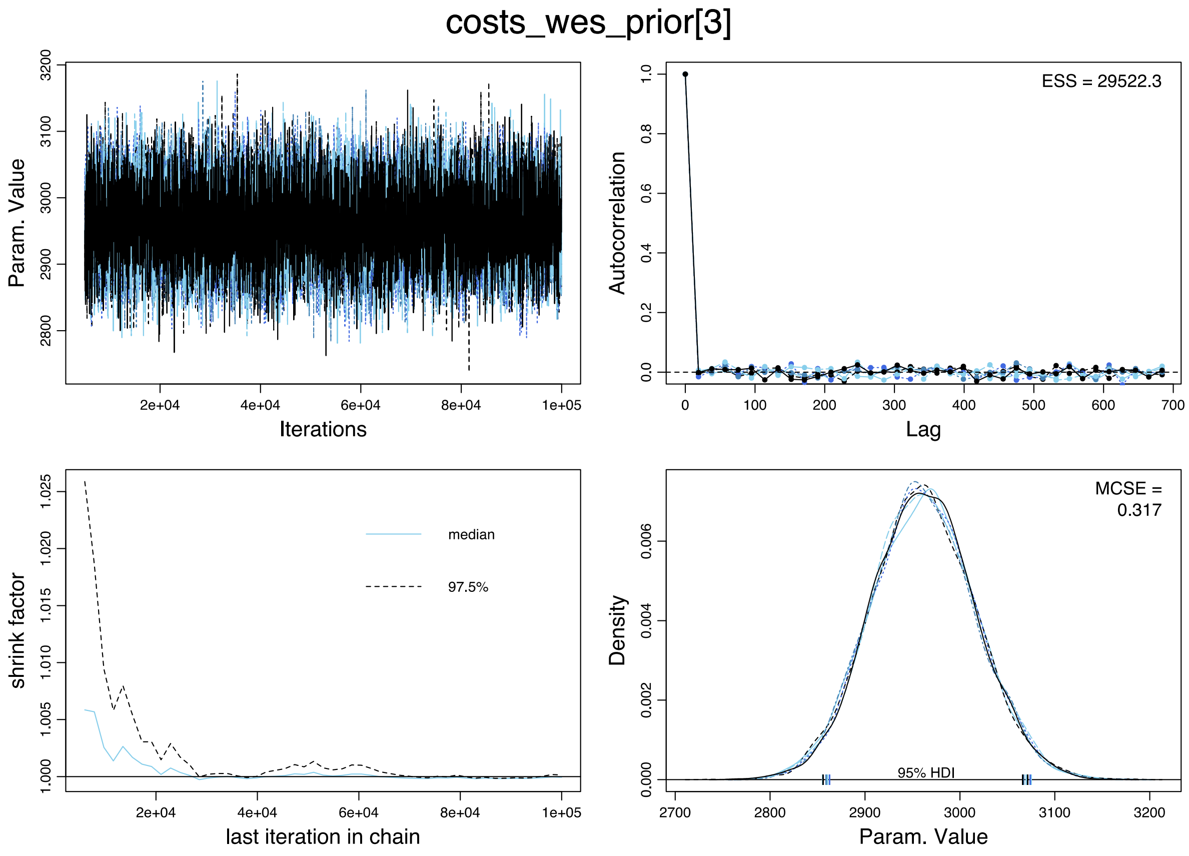

© 2024 Nurchis MC et al. *JAMA Network Open*.

eFigure 55. Convergence Diagnostics for the Output of JAGS Related to the Cost Parameter of the WES Strategy in the Eighth Health State (Prior)

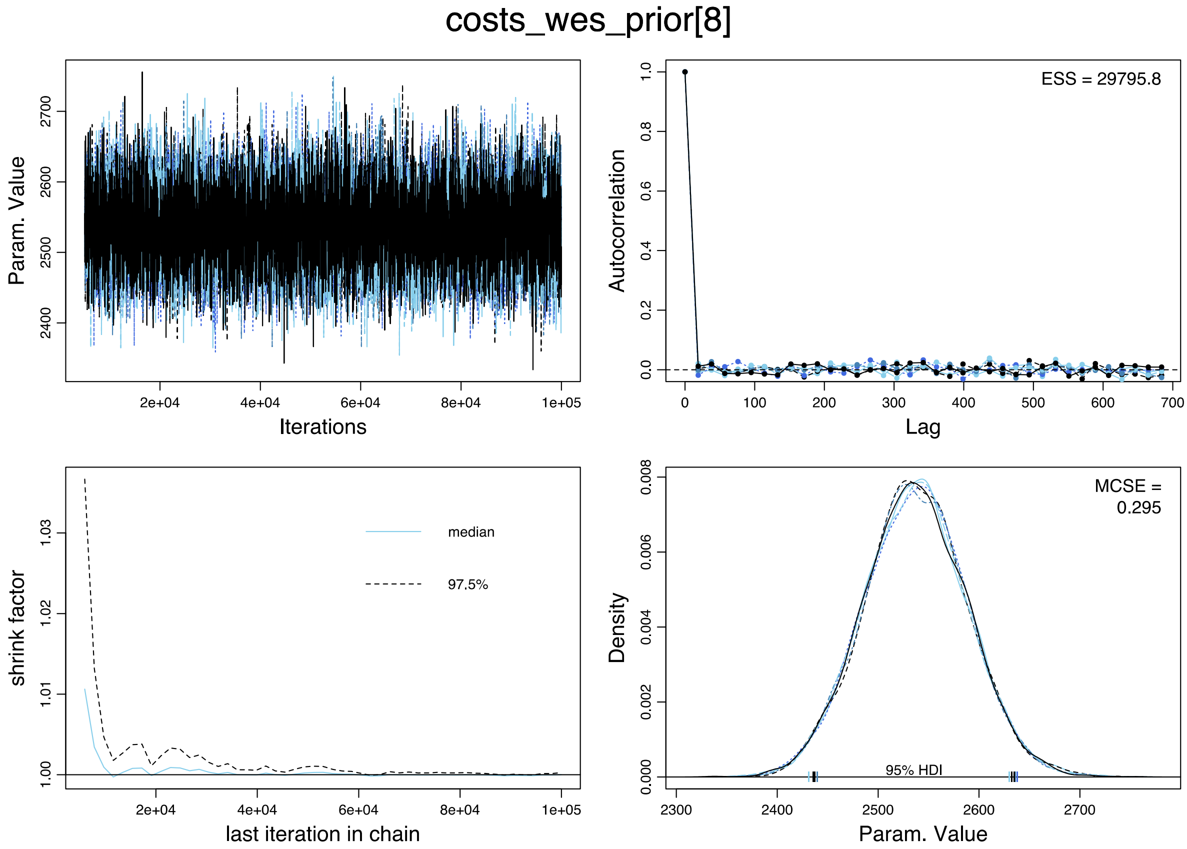

© 2024 Nurchis MC et al. *JAMA Network Open*.

eFigure 56. Convergence Diagnostics for the Output of JAGS Related to the Cost Parameter of the WGS Strategy in the First Health State (Prior)

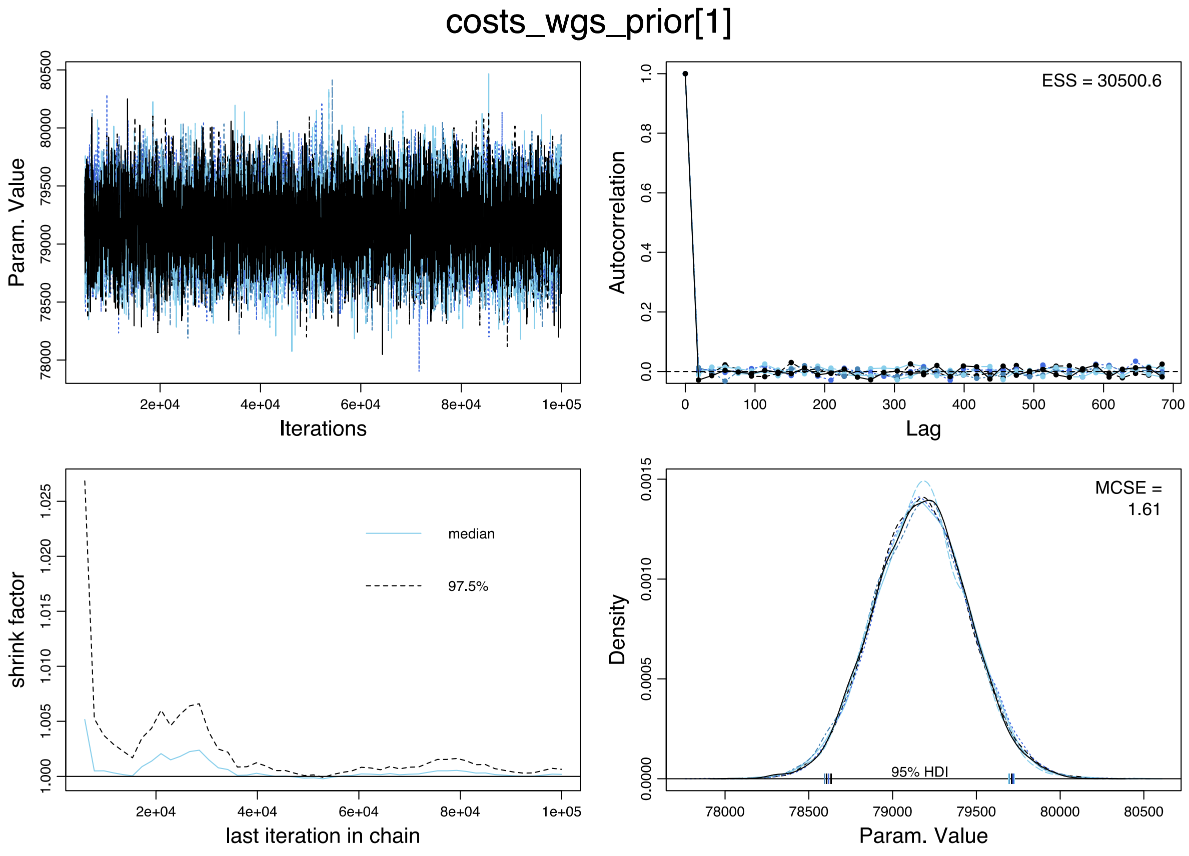

© 2024 Nurchis MC et al. *JAMA Network Open*.

eFigure 57. Convergence Diagnostics for the Output of JAGS Related to the Cost Parameter of the WGS Strategy in the Second Health State (Prior)

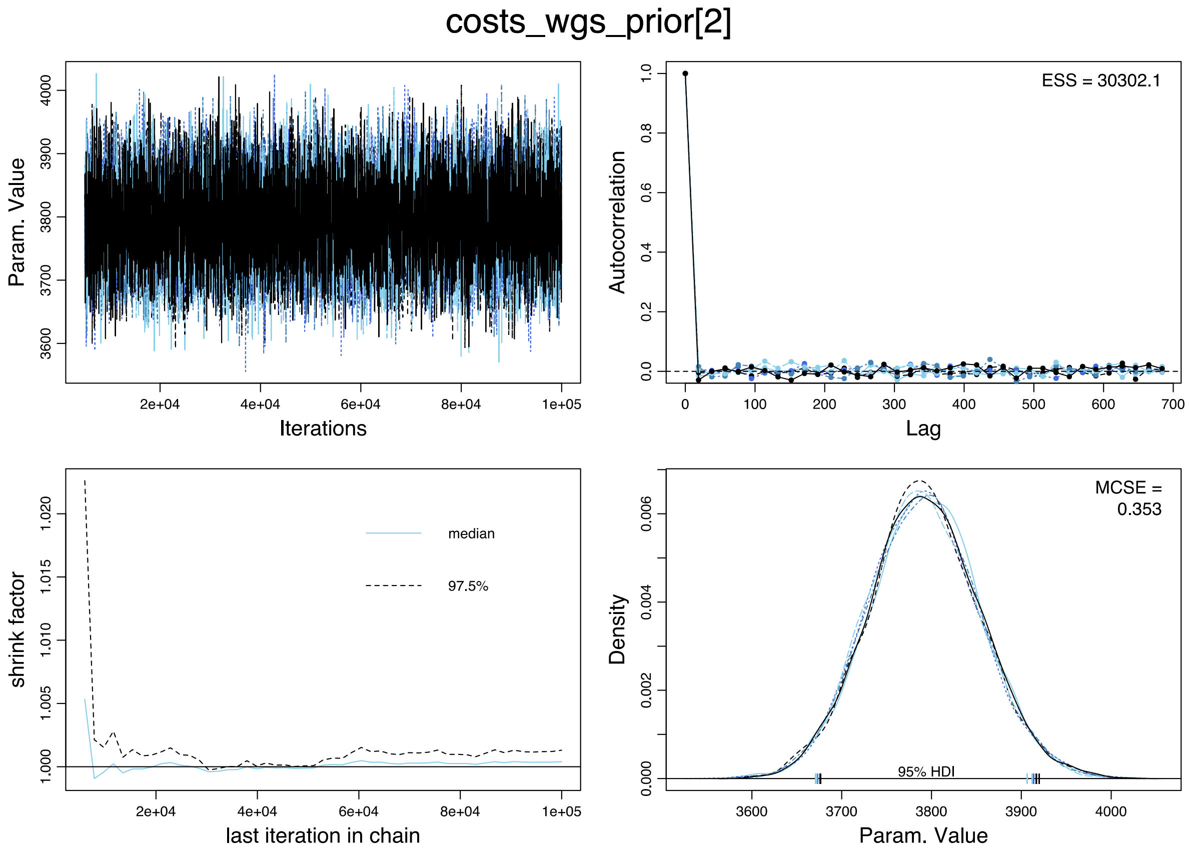

© 2024 Nurchis MC et al. *JAMA Network Open*.

eFigure 58. Convergence Diagnostics for the Output of JAGS Related to the Cost Parameter of the WGS Strategy in the Eighth Health State (Prior)

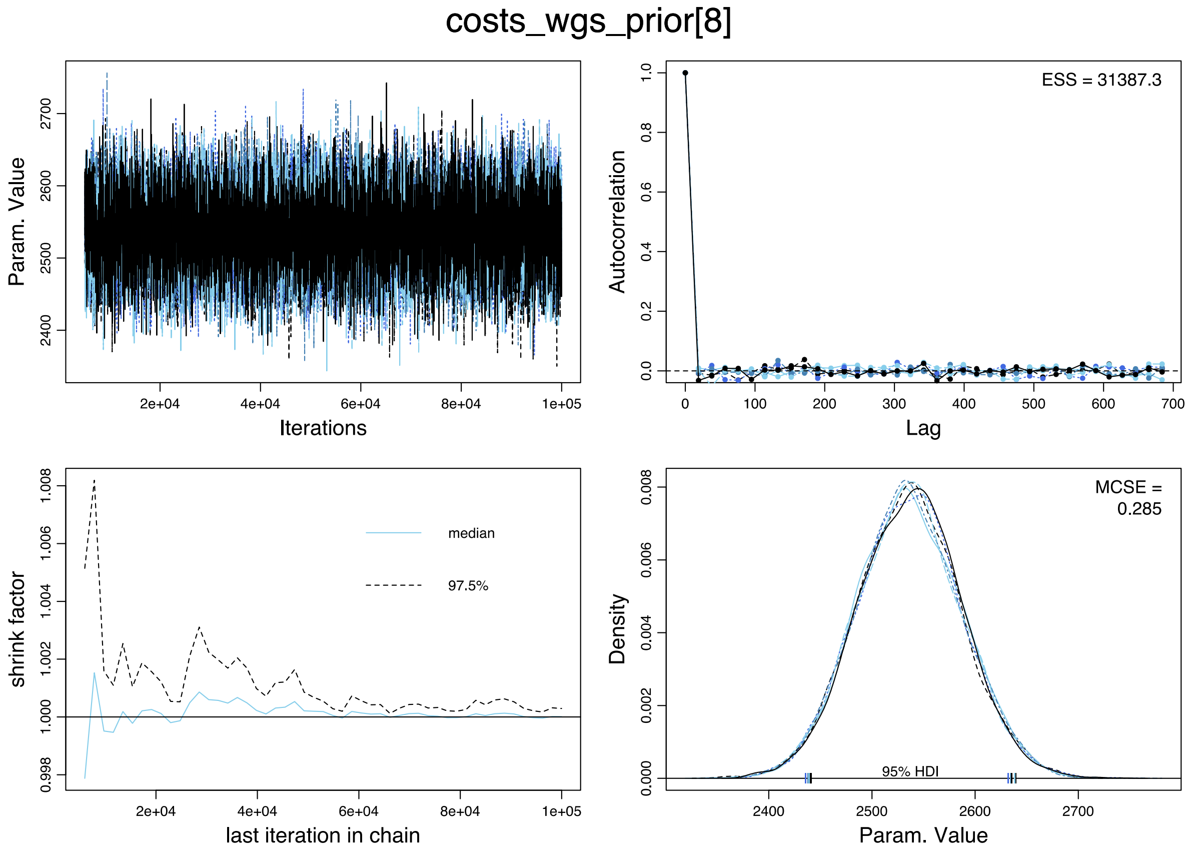

© 2024 Nurchis MC et al. *JAMA Network Open*.

eTable 1. Robustness Analysis Results Over Lifetime Horizon

Strategy	Costs (€)<br>Costs (US$)<br>(95% CI €)	Δ costs (€)<br>Δ costs (US$)<br>(95% CI €)	Eff.<br>(95% CI)<br>(n. of diagnosis)	Δ Eff.<br>(n. of diagnosis)<br>(95% CI)	ICER (€)<br>ICER (US$)<br>(95% CI €)	NMB (€)<br>NMB (US$)
WGS	€11,691,195,772<br>$12,714,175,402<br>(11,681,194,099–11,711,197,445)	–	533,568<br>(519,289–541,848)	–	Ref.	–
SOC	€5,057,770,232<br>$5,500,325,127<br>(5,032,769,308–5,084,771,156)	€6,633,425,540<br>$7,213,850,275<br>(6,623,423,625–6,648,427,455)	362,295<br>(358,015–366,575)	171,273<br>(168,994–175,553)	€38,730<br>$42,119<br>(36,025–39,490)	€1,495,235,540<br>$1,626,068,650
WES	€10,399,942,295<br>$11,309,937,246<br>(10,369,940,704–10,415,943,887)	€1,291,253,476<br>$1,404,238,155<br>(1,295,253,558–1,311,253,395)	496,249<br>(489,969–505,529)	37,319<br>(29,040–39,599)	€34,600<br>$37,628<br>(32,373–44,045)	€171,683,476<br>$186,705,780
SOC+WES	€5,477,123,780<br>$5,956,372,111<br>(5,431,122,728–5,499,124,832)	€6,214,071,992<br>$6,757,803,291<br>(6,214,070,018–6,250,073,965)	363,193<br>(340,926–375,473)	170,375<br>(166,096–178,655)	€36,473<br>$39,664<br>(34,172–37,893)	€1,102,821,992<br>$1,199,318,916
SOC+WGS	€8,027,895,037<br>$8,730,335,853<br>(8,002,893,496–8,065,896,578)	€3,663,300,735<br>$3,983,839,549<br>(3,645,298,455–3,678,303,014)	365,034<br>(338,755–392,314)	172,537<br>(167,257–176,816)	€21,232<br>$23,090<br>(20,652–23,845)	€1,512,809,265<br>$1,645,180,076

Notes: ICERs were computed as the difference between the costs of the intervention (i.e., WGS) and the comparators (i.e., SOC, WES, second-line WES, and second-line WGS, respectively) over the difference between the number of diagnoses of the intervention and the comparators. NMBs were computed considering the lower bound (i.e., €30,000) of the Eurozone threshold.
Abbreviations: CI, confidence intervals; eff., effectiveness; ICER, Incremental Cost-effectiveness Ratio; NMB, Net Monetary Benefit; Ref., reference.

© 2024 Nurchis MC et al. *JAMA Network Open*.

eTable 2. Summary Statistics of the Marginal Posterior Distribution for Each of the Model Parameters

**Parameters**	**Mean**	**SD**	**Lower bound (2.5%)**	**Upper bound (97.5%)**	**R-hat**	**N.eff**
DY_soc_prior[2]	0.42	0.66	0.03	1.96	1.001	30000
DY_soc_prior[3]	0.43	0.65	0.03	2.05	1.001	30000
DY_soc_t_tmp[2]	5924.51	11442.67	84.04	32103.42	1.001	30000
DY_soc_t_tmp[3]	6113.27	10885.84	108.23	32763.70	1.001	30000
DY_soc_wes_prior[2]	0.43	0.66	0.03	2.02	1.001	30000
DY_soc_wes_prior[3]	0.43	0.63	0.03	2.05	1.001	30000
DY_soc_wes_prior[4]	0.58	0.77	0.05	2.51	1.001	30000
DY_soc_wes_prior[5]	0.59	0.80	0.05	2.56	1.001	15000
DY_soc_wes_t_tmp[2]	5947.63	10864.90	87.96	32453.59	1.001	30000
DY_soc_wes_t_tmp[3]	6066.45	10703.75	103.10	32108.80	1.001	18000
DY_soc_wes_t_tmp[4]	5.56	190.80	-315.35	340.33	1.001	25000
DY_soc_wes_t_tmp[5]	6.94	206.03	-321.18	368.77	1.002	20000

© 2024 Nurchis MC et al. *JAMA Network Open*.

DY_soc_wgs_prior[2]	0.44	0.67	0.03	2.06	1.001	30000
DY_soc_wgs_prior[3]	0.43	0.70	0.03	2.03	1.001	30000
DY_soc_wgs_prior[4]	0.63	0.81	0.06	2.65	1.001	30000
DY_soc_wgs_prior[5]	0.62	0.80	0.06	2.62	1.001	15000
DY_soc_wgs_t_tmp[2]	6040.80	11002.46	84.82	32142.98	1.001	30000
DY_soc_wgs_t_tmp[3]	6062.46	11400.88	96.09	32048.48	1.001	30000
DY_soc_wgs_t_tmp[4]	6.40	205.66	-336.90	374.10	1.002	30000
DY_soc_wgs_t_tmp[5]	6.33	201.46	-333.00	370.74	1.002	30000
DY_wes_prior[2]	0.58	0.77	0.05	2.51	1.001	30000
DY_wes_prior[3]	0.59	0.80	0.05	2.56	1.001	15000
DY_wes_t_tmp[2]	8167.51	12971.33	149.41	41250.77	1.001	30000
DY_wes_t_tmp[3]	8409.09	13892.56	179.51	41717.08	1.001	21000
DY_wgs_prior[2]	0.63	0.81	0.06	2.65	1.001	30000
DY_wgs_prior[3]	0.62	0.80	0.06	2.62	1.001	15000
DY_wgs_t_tmp[2]	8829.44	13701.97	168.33	43984.89	1.001	30000
DY_wgs_t_tmp[3]	8768.94	13744.78	196.19	43108.41	1.001	30000
cost_soc_t_tmp[1]	28998148.80	167037.78	28674190.90	29325026.02	1.001	18000
cost_soc_t_tmp[2]	6270425.94	3697816.83	305895.10	12615324.60	1.001	30000
cost_soc_t_tmp[3]	6399314.12	3664659.66	367255.05	12680526.53	1.001	30000
cost_soc_t_tmp[8]	1347700.37	27338709.43	-51086475.66	53124042.14	1.001	30000

© 2024 Nurchis MC et al. *JAMA Network Open*.

cost_soc_wes_t_tmp[1]	44455386.10	209739.10	44048047.32	44871945.57	1.001	30000
cost_soc_wes_t_tmp[2]	6271519.76	3711417.63	310571.53	12675385.50	1.001	30000
cost_soc_wes_t_tmp[3]	6368465.00	3653774.48	380922.17	12620621.97	1.001	12000
cost_soc_wes_t_tmp[4]	29074.24	574507.73	-1168830.73	1248829.73	1.001	30000
cost_soc_wes_t_tmp[5]	29331.81	604026.79	-1236067.77	1307994.39	1.001	13000
cost_soc_wes_t_tmp[8]	76610.61	1628165.34	-2750393.09	2977525.45	1.001	13000
cost_soc_wgs_t_tmp[1]	52932827.08	226632.21	52490587.65	53372926.92	1.001	27000
cost_soc_wgs_t_tmp[2]	6269875.13	3720640.61	295430.77	12653897.46	1.001	30000
cost_soc_wgs_t_tmp[3]	6358316.10	3664721.93	352184.96	12637366.40	1.001	30000
cost_soc_wgs_t_tmp[4]	41423.42	757095.32	-1517831.84	1646679.62	1.001	30000
cost_soc_wgs_t_tmp[5]	39533.65	783538.73	-1636108.25	1688646.58	1.001	30000
cost_soc_wgs_t_tmp[8]	63772.22	1650178.96	-2880006.81	3007410.20	1.001	30000
cost_wes_t_tmp[1]	59906961.09	239224.47	59441240.84	60376775.51	1.001	19000
cost_wes_t_tmp[2]	40608233.76	23709246.95	1994546.96	80500667.72	1.001	30000
cost_wes_t_tmp[3]	42472091.56	23888409.89	2670184.97	82609690.80	1.001	30000
cost_wes_t_tmp[8]	885353.33	29194563.58	-54770235.33	56263964.85	1.001	30000
cost_wgs_t_tmp[1]	76864927.41	273667.14	76335137.98	77399165.67	1.001	15000

© 2024 Nurchis MC et al. *JAMA Network Open*.

cost_wgs_t_tmp[2]	53462823.85	31131882.54	2666989.06	105761389.11	1.001	30000
cost_wgs_t_tmp[3]	54813702.30	31233098.18	3313357.08	107650388.60	1.001	30000
cost_wgs_t_tmp[8]	1122103.19	29342585.95	-54836470.58	56376883.09	1.001	30000
costs_soc_prior[1]	29868.09	172.05	29534.42	30204.78	1.001	18000
costs_soc_prior[2]	449.82	21.20	409.45	492.49	1.001	30000
costs_soc_prior[3]	450.00	21.31	409.17	493.29	1.001	30000
costs_soc_prior[8]	2374.73	48.89	2281.61	2471.80	1.001	30000
costs_soc_wes_prior[1]	45789.05	216.03	45369.49	46218.10	1.001	30000
costs_soc_wes_prior[2]	449.88	21.22	409.96	493.20	1.001	30000
costs_soc_wes_prior[3]	449.82	21.18	409.13	492.70	1.001	25000
costs_soc_wes_prior[4]	2892.15	53.89	2787.34	2999.18	1.001	30000
costs_soc_wes_prior[5]	2961.46	54.72	2855.62	3069.58	1.001	30000
costs_soc_wes_prior[8]	2536.93	50.23	2440.84	2637.37	1.001	30000
costs_soc_wgs_prior[1]	54520.81	233.43	54065.31	54974.11	1.001	27000
costs_soc_wgs_prior[2]	450.04	21.18	409.51	492.92	1.001	30000
costs_soc_wgs_prior[3]	450.02	21.02	409.93	491.99	1.001	30000
costs_soc_wgs_prior[4]	3792.18	61.38	3673.69	3915.44	1.001	14000
costs_soc_wgs_prior[5]	3862.29	62.02	3740.79	3984.78	1.001	16000
costs_soc_wgs_prior[8]	2536.73	50.45	2439.81	2637.10	1.001	25000
costs_wes_prior[1]	61704.17	246.40	61224.48	62188.08	1.001	19000

© 2024 Nurchis MC et al. *JAMA Network Open*.

costs_wes_prior[2]	2892.07	53.69	2788.94	2999.99	1.001	28000
costs_wes_prior[3]	2961.77	54.41	2856.35	3069.55	1.001	30000
costs_wes_prior[8]	2537.26	50.88	2438.28	2637.01	1.001	30000
costs_wgs_prior[1]	79170.88	281.88	78625.19	79721.14	1.001	15000
costs_wgs_prior[2]	3792.18	61.38	3673.69	3915.44	1.001	14000
costs_wgs_prior[3]	3862.29	62.02	3740.79	3984.78	1.001	16000
costs_wgs_prior[8]	2536.40	50.44	2439.12	2636.61	1.001	30000
trans_mat_soc_prior[1,2]	0.49	0.29	0.02	0.97	1.001	30000
trans_mat_soc_prior[1,3]	0.49	0.28	0.03	0.97	1.001	30000
trans_mat_soc_prior[1,8]	0.02	0.40	-0.75	0.78	1.001	30000
trans_mat_soc_wes_prior[1,2]	0.49	0.29	0.02	0.97	1.001	30000
trans_mat_soc_wes_prior[1,3]	0.49	0.28	0.03	0.97	1.001	13000
trans_mat_soc_wes_prior[8,4]	0.49	0.29	0.02	0.97	1.001	30000
trans_mat_soc_wes_prior[8,5]	0.50	0.28	0.03	0.97	1.001	30000
trans_mat_soc_wes_prior[1,8]	0.02	0.40	-0.75	0.78	1.001	16000
trans_mat_soc_wes_prior[8,8]	0.01	0.40	-0.75	0.77	1.001	30000
trans_mat_soc_wgs_prior[1,2]	0.49	0.29	0.02	0.97	1.001	30000
trans_mat_soc_wgs_prior[1,3]	0.49	0.28	0.03	0.97	1.001	30000
trans_mat_soc_wgs_prior[8,4]	0.49	0.29	0.02	0.97	1.001	30000
trans_mat_soc_wgs_prior[8,5]	0.49	0.28	0.03	0.97	1.001	30000
trans_mat_soc_wgs_prior[1,8]	0.02	0.40	-0.75	0.78	1.001	30000

© 2024 Nurchis MC et al. *JAMA Network Open*.

trans_mat_soc_wgs_prior[8,8]	0.02	0.40	-0.75	0.77	1.001	30000
trans_mat_wes_prior[1,2]	0.49	0.29	0.02	0.97	1.001	30000
trans_mat_wes_prior[1,3]	0.50	0.28	0.03	0.97	1.001	30000
trans_mat_wes_prior[1,8]	0.01	0.40	-0.75	0.77	1.001	30000
trans_mat_wgs_prior[1,2]	0.49	0.29	0.02	0.97	1.001	30000
trans_mat_wgs_prior[1,3]	0.49	0.28	0.03	0.97	1.001	30000
trans_mat_wgs_prior[1,8]	0.02	0.40	-0.75	0.77	1.001	30000

The interval encompassing the 2.5% and 97.5% quantiles represents the 95% credible range for each parameter, which is the Bayesian counterpart to a confidence interval. The convergence diagnostics are presented in the final two columns of the table.

© 2024 Nurchis MC et al. *JAMA Network Open*.
