# Supplementary material for: Cost-Effectiveness of Whole-Genome vs Whole-Exome Sequencing Among Children With Suspected Genetic Disorders
Source: JAMA Netw Open. 2024 Jan 26;7(1):e2353514. doi: 10.1001/jamanetworkopen.2023.53514 (PMC10818217; doi:10.1001/jamanetworkopen.2023.53514)
Supplement: Supplement 2. — Data Sharing Statement [file jamanetwopen-e2353514-s002.pdf]

Data Sharing Statement

Nurchis. Cost-Effectiveness of Whole-Genome vs Whole-Exome Sequencing Among Children With Suspected Genetic Disorders. *JAMA Netw Open*. Published January 26, 2024. doi:10.1001/jamanetworkopen.2023.53514

Data
Data available: No

Additional Information
Explanation for why data not available: Only aggregated data were employed for this study. Of note, we can share the R scripts with the codes and the aggregated input parameters used in the model.
